# Development and Validation of the Cheers Attitudes towards Non-drinkers Scale (CANS)

**DOI:** 10.1177/13591053231220519

**Published:** 2024-01-29

**Authors:** Christopher Cheers, Amy Pennay, Xochitl de la Piedad Garcia, Sarah Callinan

**Affiliations:** 1 La Trobe University, Australia; 2 Australian Catholic University, Australia

**Keywords:** alcohol, attitudes, non-drinker, scale development, stigma

## Abstract

Non-drinkers report experiencing stigma, which can act as a barrier to non-drinking. Two studies were undertaken to develop and test a new scale to measure attitudes towards non-drinkers. In Study 1, 29 items were presented to 426 Australian drinkers. In Study 2, the refined 12-item Cheers Attitudes to Non-drinkers Scale (CANS) was presented to 389 drinkers. Alcohol consumption, Harm and the Regan Attitudes towards Non-drinkers Scale (RANDS) were presented for scale validation. Exploratory factor analysis revealed three factors representing the Threats to Fun, Connection and Self that drinkers perceive non-drinkers to pose. Confirmatory factor analysis showed that the scale meets the required fit indices and had good reliability (α = 0.842). Evidence of validity was shown through significant correlations with Alcohol Consumption, Harm and the RANDS. These studies show the CANS to be a reliable and valid measure that could be utilised to understand and modify the stigma experienced by non-drinkers.

Non-drinking in Australia is becoming a more common practice. The proportion of Australians (14+ years) who abstain (i.e. have never drunk alcohol or not had a drink in the previous 12 months) has increased in recent years, with abstention rates rising from 17% in 2007 to 23% in 2019 (Australian Institute of Health and Welfare [AIHW], 2020). This increase has been driven primarily by young people, with 56% of 14–19-year-olds abstaining from alcohol in 2019, compared to 30% in 2007 (AIHW, 2020). This trend is also evident in other high-income countries, with the number of young adults in the United States (aged 18–22 years) who abstained from alcohol increasing from 24% in 2018 to 30% in 2022 (McCabe et al., 2021). However, despite more people choosing not to drink, research continues to show that the choice not to drink is often met with stigma and negative evaluation (Bartram et al., 2017; Cherrier and Gurrieri, 2013).

Research consistently shows that non-drinkers are rated less favourably than drinkers (Davies et al., 2016; Litt and Lewis, 2015; van Lettow et al., 2013). This judgement appears predominantly based on a view that non-drinkers are less sociable than drinkers (Conroy and de Visser, 2013, 2016; Davies et al., 2016; Rivis et al., 2006; Young et al., 2016; Zimmermann and Sieverding, 2011). For example, student populations are shown to be broadly permissive of heavy drinking (Conroy and de Visser, 2014) and often evaluate non-drinkers as ‘uncool’ and ‘unsociable’ (Rivis et al., 2006), and non-drinking as something ‘strange requiring explanation’ (Conroy and de Visser, 2013). Conroy and de Visser (2016) examined the perceived sociability of non-drinker prototypes in 543 English university students and found that regular drinkers were rated as significantly more sociable than non-drinkers, with 90.8% of participants viewing non-drinkers as relatively ‘unsociable’. This can be linked to research in which heavy drinking is perceived to be an important mechanism for peer bonding and developing a sense of belonging among young adults (Seaman and Ikegwuonu, 2010). Further, the judgement from drinkers is reported as a deterrent to maintaining abstention and reduction in alcohol use (Conroy and de Visser, 2014; Robertson and Tustin, 2018), with the stigmatising reactions towards non-drinking encouraging the consumption of alcohol to avoid social judgement or damage to peer relationships (Bartram et al., 2017).

In response to the growing need to better understand the stigma experienced by non-drinkers, Regan and Morrison (2011) developed the Regan Attitudes towards Non-Drinkers Scale (RANDS). The RANDS is a single-factor scale, with 11 items measuring the ‘social costs of non-drinking’. In samples of Irish university students (Regan and Morrison, 2011, 2017) and Irish secondary school students (Regan and Morrison, 2013), this scale has demonstrated promising psychometric properties with good scale reliability (with Cronbach’s alpha values range of 0.82–0.89 across diverse samples) and test re-test reliability (intraclass correlation coefficient 0.86).

Our previous research (Cheers et al., 2021) extended the work of Regan and Morrison (2011), finding that in addition to the perceived ‘social costs of non-drinking’, the stigma towards non-drinkers is also driven by a need to maintain ego. Specifically, through interviews with Australian drinkers and non-drinkers, the study found attitudes towards non-drinkers are primarily based on three ‘threats’ that non-drinkers were perceived to pose to drinkers. Supporting the work of Regan and Morrison, two of the threats related clearly to sociability, with non-drinkers perceived as a (1) *threat to fun* (e.g. a judgemental ‘sober eye’ disrupting the desired hedonistic environment created by alcohol) and (2) *threat to connection* (e.g. difficult to initiate and maintain social connection with). However, through analysis of interview data, Cheers et al. (2021) identified a third threat, a *threat to self*, which reflected the consistent theme that beyond the social costs, the presence of a non-drinker encouraged an unwanted self-reflection on the problematic aspects of the drinker’s own drinking. In addition, mirroring previous research (Conroy and de Visser, 2013; Peralta, 2007), the findings of Cheers et al. (2021) highlighted how the gender of the non-drinker influenced all three factors, with drinkers describing non-drinking as less acceptable for men than women.

Through the lens of Intergroup Threat Theory (Stephan and Stephan, 2013), the *threat to self* stigma domain proposed by Cheers et al. (2021) can be understood as a process of ego-regulation (Baumeister et al., 1994), and specifically of self-affirmation (Steele, 1988). In this framework, devaluing others becomes a means for drinkers to defend against reflecting on their own (potentially unhealthy) alcohol consumption. This theory suggests those who drink at levels that risk their health will be more likely to defend their ego, leading to more negative attitudes towards non-drinkers. This model provides an explanation for the consistent finding that more negative attitudes towards non-drinkers are associated with greater alcohol consumption (Regan and Morrison, 2013, 2017; Young et al., 2016; Zimmermann and Sieverding, 2010, 2011).

The current research aims to extend the work of Regan and Morrison and their single-factor model of attitudes towards non-drinkers by developing and validating a new scale based on the *Triple Threat Theory* (Cheers et al., 2021). This validation will include an examination of criterion validity through a hypothesised significant positive correlation between the CANS and the other known measure of the same construct, the RANDS. Construct validity will also be examined based on previous research (Regan and Morrison, 2017; Young et al., 2016) the CANS is hypothesised to show a significant positive correlation to a graduated frequency measure of total Alcohol Consumption (Greenfield et al., 2009) and the Alcohol Use Disorders Identification Test (a measure of risky alcohol use and harm; Saunders et al., 1993). Through this process, this study also aims to reveal if the new threat-based conceptualisation of attitudes towards non-drinkers, until now only established in a small sample qualitative study, is maintained in two larger, more diverse samples.

It is proposed that the ability to measure the contribution of different components of the stigma towards non-drinkers will have important implications for the development of health promotion strategies that aim to create a more supportive environment for people choosing to reduce their alcohol consumption. Although the RANDS shows good psychometric properties, most of the questions measure a drinker’s predicted attitude towards themselves if they were to become a non-drinker (e.g. ‘If I were a non-drinker, I believe my friends would treat me differently’, and ‘I think being a non-drinker would negatively affect my life’). It is proposed a scale that more directly measures drinkers’ attitudes towards non-drinkers, and the known components of this stigma would make a meaningful contribution to research aiming to reduce alcohol harm in the community by shifting negative attitudes towards non-drinkers. For example, by identifying which aspects of non-drinking are negatively appraised across large samples (e.g. a Threat to Fun, Connection or Self), health promotion strategies that target these constructs are more specifically developed.

The development of the new scale is presented across two studies. Study 1 presents the development of items and a final scale utilising exploratory factor analysis, and Study 2 presents a confirmatory factor analysis of the scale within a new sample.

## Study 1

### Method

#### Participants

We recruited 426 Australian adults aged 18–50 years (*M_age_* = 37.35 years, SD = 9.37) from April to May 2021 using advertisements on Facebook and Instagram, which invited drinkers to participate with the chance to enter a draw to win one of four $50 supermarket vouchers. Eligibility was established by an initial set of questions in which participants indicated they had consumed a drink containing alcohol in the previous 12 months, were aged 18–50 years, and lived in Australia. The sample was comprised of 225 women (52.8%), 189 men (44.4%), 9 non-binary or other specified gender (2.8%), and 3 (0.7%) who preferred not to indicate gender (for further demographics, see Supplemental Table 1). This sample size was determined as adequate to perform the exploratory factor analysis as there were more than 10 participants per scale item (Terwee et al., 2007) and the total sample was above 300 (MacCallum et al., 1999; Worthington and Whittaker, 2006).

#### Measures

##### Cheers Attitudes towards Non-drinkers Scale (CANS)

Based on the qualitative data obtained from drinkers interviewed in Cheers et al. (2021), a list of items was generated for each theme, with 14 items representing a *Threat to Fun*, 26 items for the *Threat to Connection* and 22 for the *Threat to Self*. These 62 items were then assessed by the authors (including two experts in the field) to select the best 10 items for each theme. These 30 items were then examined for content validity by a further three experts in the field and three members of the target population (e.g. adult drinkers). Items were assessed on three aspects of content validity (1) relevance (all items should be relevant for the construct of interest within a specific population and context of use), (2) comprehensiveness (no key aspects of the construct should be missing) and (3) comprehensibility (the items should be understood by participants as intended; Terwee et al., 2007). One item was removed and 15 were amended based on the responses of the experts and target populations (see Supplemental Material for specific information regarding item development). This process resulted in a final set of 29 items which was used in the present study. Respondents indicated the level of agreement on each item (e.g. threat to self: ‘Non-drinkers make me think about my own drinking’, threat to fun: ‘Non-drinkers spoil the fun’, threat to connection: ‘It’s hard to get to know non-drinkers’) on a 5-point Likert-like scale (1 = strongly disagree; 5 = strongly agree).

##### Regan Attitudes towards Non-drinkers Scale (RANDS)

This 11-item scale examines attitudes towards non-drinkers (e.g. ‘I would hate to be a non-drinker’). It uses a 5-point Likert scale (1 = strongly disagree; 5 = strongly agree). The total score ranges from 11 to 55, with higher scores denoting more negative attitudes. Scale score reliability is good (alpha values range 0.82–0.89; Regan and Morrison, 2011, 2017).

##### Graduated frequency of total alcohol consumption (GF)

This section consisted of six questions in which participants indicated the frequency with which they had consumed different quantities of standard drinks within a single day in the last 12 months for example, ‘In the last 12 months how often have you had [number of] standard drinks in one day?’ (Greenfield et al., 2009). The specific number of standard drinks across questions were ‘1–2’, ‘3–4’, ‘5–6’, ‘7–10’, ‘11–19’ and ‘20 or more’ standard drinks per day. For each, participants indicated how often they consumed that range of standard drinks that is, ‘every day’, ‘5–6 days a week’, ‘3–4 days a week’ or ‘1–2 days a week’, ‘about 1 day a month’, ‘less often’ or ‘never’. To establish a total score, each participants response (number of times per day) was converted to an equivalent number of days per year, and then multiplied by the mid-point of the interval of standard drinks in that question (e.g. 3–4 standard drinks was converted to 3.5). These were then summed to create a total amount of standard drinks consumed per year. This was achieved through a formula in Stata (StataCorp, 2021) that ensured that only a maximum of 365 drinking days could contribute to a participant’s total.

##### Alcohol Use Disorders Identification Test (AUDIT)

The AUDIT is a 10-item screening measure for unhealthy alcohol use, defined as risky or hazardous consumption or any alcohol use disorder. Items 1–3 measure levels of *Alcohol Consumption* (e.g. ‘How many standard drinks containing alcohol do you have on a typical day when drinking?’) and items 4–6 measure *Alcohol Dependence Symptoms* (e.g. ‘During the past year, how often have you needed a drink in the morning to get yourself going after a heavy drinking session?’). Items 7–10 measure *Alcohol-related Harm* (e.g. ‘During the past year, how often have you been unable to remember what happened the night before because you had been drinking?’). For these first eight items, participants’ responses are scored on a scale from 0 to 4. For items 9–10 (e.g. ‘Have you or someone else been injured as a result of your drinking?’), participants indicate ‘no’, ‘yes, but not in the past year’ or ‘yes, during the past year’, scored at 0, 2 or 4, respectively. These three subscales can also be summed to form a total score that can be used to place participants into one of four ordinal categories: low risk (0–7); hazardous (7–15); harmful (16–19) and high risk (20 and over) or into the binary categories of low risk (non-hazardous; <8) and hazardous (risky; ⩾8) drinking (Saunders et al., 1993).

#### Procedure

Participants completed all measures on an online survey using Qualtrics software (Qualtrics, 2021, Provo, UT). After indicating agreement on the 29 initial CANS items, participants completed the RANDS and the GF, followed by the AUDIT. Participants were invited to enter their contact details after the survey (details were stored separately to ensure anonymity).

#### Data analysis

Factor Analysis (as opposed to Principal-Components Analysis) was used as it is recommended for the development of new scales when they aim to reveal an underlying factor structure of a set of items that express an underlying latent construct without imposing any preconceived structure (Worthington and Whittaker, 2006). Specifically, to test structural validity, an exploratory factor analysis (EFA) was run with principal axis factoring, parallel analysis to determine the optimal number of factors and oblimin rotation (oblique) as this is considered the best practice method when all items are expected to be correlated (Worthington and Whittaker, 2006). Cronbach’s alpha values were computed for each CANS subscale and the total score to examine internal consistency. Criterion (concurrent) was tested by correlation of the CANS with the RANDS. Construct (convergent) validity was be tested by correlation of the CANS with two measures of alcohol consumption: AUDIT and GF.

### Results

#### Exploratory factor analysis (EFA)

Inter-item correlations showed that all items correlated adequately with other items in the scale (at least some inter-item correlations above 0.3, and most not above 0.8; Cristobal et al., 2007). All items correlated at least 0.3 with at least one other item, suggesting reasonable factorability. The Kaiser-Meyer-Olkin measure of sampling adequacy was 0.940 (above recommended 0.6; Tabachnick et al., 2007), and Bartlett’s test of sphericity was significant (χ^2^(406) = 7125, *p* < 0.001). Taken together this indicated the data was suitable to run the EFA.

Factor extraction was then conducted using parallel analysis and showed a three-factor solution. An EFA extracting three factors was run on all 29 items using the oblimin rotation. Items were deleted when factor loadings were less than 0.32 on any item, when an item’s absolute loadings were higher than 0.32 on two or more factors, or when communalities were less than 0.3 (Tabachnick et al., 2007). Based on these criteria, 11 items were removed through an iterative process (see Supplemental Material for additional information). The remaining 18 items retained the three-factor structure with four items consistent with the *Threat to Connection*, five with the *Threat to Self* and nine with the *Threat to Fun.* To enhance the optimal structure of the scale, a thorough examination of each item was conducted to assess its meaningful contribution to the factor. Repetitive items were systematically removed to optimise the scale’s length and overall effectiveness. Based on this analysis, five items were deleted from the *Threat to Fun* factor: two items were too closely alike and highly correlated, and three items did not fit meaningfully with the other items in the factor. One item was removed from the *Threat to Self* factor as it was inconsistent with the rest of the items in that factor (See Supplemental Material for additional information). A final three-factor structure with 12 items was determined (shown in Table 1) and was shown to explain 52.4% of the total variance.

**Table 1.** Reliability, means, standard deviations and factor loadings of exploratory factor analysis (EFA) of the Cheers Attitude to Non-drinkers Scale (CANS).

	EFA		
	*M*	SD	Absolute loading
Factor 1: Threat to fun (α = 0.810)			
1. Non-drinkers spoil the fun.	1.62	0.792	0.841
2. Non-drinkers make it harder for me to relax.	1.75	0.921	0.799
3. I am conscious of not having too much fun around non-drinkers at parties.	1.83	1.01	0.623
4. I sometimes want non-drinkers to drink to make me more comfortable.	1.87	0.965	0.600
Factor 2: Threat to self (α = 0.780)			
5. Non-drinkers are making a healthier choice than me.	3.74	1.05	0.829
6. If I was a non-drinker, I would be healthier.	3.54	1.14	0.792
7. Non-drinkers are making a choice I wish I could make.	2.60	1.14	0.590
8. Non-drinkers make me think about my own drinking.	3.01	1.26	0.531
Factor 3: Threat to connection (α = 0.793)			
9. Drinking makes it easier to make new friends.	3.10	1.14	0.771
10. Alcohol provides a short-cut to social bonding.	3.57	0.975	0.755
11. Getting to know someone is easier if they are drinking.	2.91	1.14	0.747
12. I believe the bonds made over drinks are the strongest ones.	2.33	0.993	0.563

Extraction Method: Principal Axis Factoring; Rotation Method: Oblimin.

#### Internal consistency

The final 12-item scale demonstrated strong internal consistency, as indicated by a Cronbach’s Alpha of 0.824. Internal consistency for each factor (e.g. subscale) was further examined using Cronbach’s alpha and were all shown to have good internal consistency (see Table 1). No increases in Cronbach’s alpha for any of the scales could have been achieved by eliminating items.

#### Validity and correlations

To assess validity, correlations between the total and each CANS subscale, RANDS, GF and AUDIT (and all AUDIT subscales) scores were examined (Table 2). All variables were shown to be significantly skewed, with all showing significant Kolmogorov-Smirnov tests (*ps* < 0.001) and all but CANS (*p* = 0.142) showing significant Shapiro-Wilk tests (*ps* < 0.001). For this reason, Spearman rank correlations were used. All variables were significantly correlated (all *ps* < 0.001); however, there were differences in the strengths of correlations, most notably concerning the *Threat to Self* subscale, which, compared to the other subscales, showed weaker correlations with the RANDS. Examination of inter-factor correlations showed that all subscales were significantly correlated to each other. However, the *Threat to Self* subscale showed a weaker correlation with both the *Threat to Fun* and *Threat to Connection* subscales, compared to the correlation between the *Threat to Fun* and *Threat to Connection* subscales (see Table 2).

**Table 2.** Spearman rank correlation coefficients (Spearman’s rho) between variables in Study 1.

	1.	2.	3.	4.	5.	6.	7.	8.	9.	10.	11.
1. CANS Total (*n* = 426)	–										
2. CANS Threat to fun	0.727***	–									
3. CANS Threat to self	0.698***	0.228***	–								
4. CANS Threat to connection	0.782***	0.506***	0.281***	–							
5. RANDS (*n* = 426)	0.634***	0.661***	0.193***	0.624***	–						
6. AUDIT Total (*n* = 404)	0.632***	0.332***	0.515***	0.538***	0.450***	–					
7. AUDIT Consumption	0.564***	0.312***	0.416***	0.515***	0.475***	0.916***	–				
8. AUDIT Dependence	0.535***	0.259***	0.467***	0.435***	0.349***	0.760***	0.624***	–			
9. AUDIT Alcohol-harm	0.532***	0.244***	0.499***	0.416***	0.291***	0.857***	0.632***	0.638***	–		
10. GF (*n* = 418)	0.564***	0.348***	0.398***	0.512***	0.468***	0.860***	0.913***	0.617***	0.608***	–	
11. Age (*n* = 426)	−0.107*	−0.063	−0.080	−0.093	−0.067	−0.046	0.010	−0.089	−0.078	−0.021	–
12. Gender (*n* = 426)	−0.275***	−0.224***	−0.160**	−0.254***	−0.226***	−0.312***	−0.346***	−0.202***	−0.185***	−0.344***	0.002

All values are Spearman’s Rho except for correlations with gender which are point-biserial correlations (male = 0, female = 1). Note differing sample sizes were due to participants exiting before completing the survey.

Age: participant’s age in years; CANS: Cheers Attitudes Towards Non-drinkers Scale total score; RANDS: Regan Attitudes to Non-drinkers total score; AUDIT: Alcohol Use Disorders Identification Test (AUDIT) total score; GF: graduated frequency of total alcohol consumption per year.

* *p* < 0.05. **p < 0.01. ***p < 0.001.

Criterion (concurrent) validity was demonstrated as total CANS scores showed a strong, significant positive correlation with the RANDS. Construct (convergent) validity was demonstrated as the total CANS score was significantly correlated with all measures of alcohol consumption, showing strong positive correlations with the AUDIT total score, each subscale, and GF. Greater alcohol consumption on both measures predicted more negative attitudes towards non-drinkers (higher CANS scores). Using Fisher’s *r*-to-*z* transformations, the AUDIT consumption subscale was shown to be significantly more related to the threat to connection subscale (compared to the other subscales), and the *Threat to Self* subscale was significantly more related to the AUDIT Alcohol-harm subscale compared to the AUDIT Consumption subscale.

Table 2 shows that age was significantly correlated with the total CANS score. However, the Spearman’s rho shows this correlation was negligible. Point-biserial correlations were performed with gender on all variables to test if there was any significant difference between means for men and women, with men coded as 0 and women as 1. All variables showed significant negative correlations, indicating men, on average, had higher scores on the total CANS score, all CANS subscale, RANDS, AUDIT and GF.

## Study 2

### Method

#### Participants

Following best practices in scale development, a new sample was recruited for a Confirmatory Factor Analysis (CFA; Flora and Flake, 2017). The sample of 389 Australian adults aged 18–70 years (*M_age_* = 39.78 years, SD = 13.50) was comprised of 211 women (54.2%), 169 men (43.4%), 7 non-binary or other specified (1.8%) and 2 (0.5%) preferred not to indicate gender (for further demographics see Supplemental Table 1). Participants were recruited (April-July 2022) using an advertisement on Facebook and Instagram. This sample size can be considered adequate as it is greater than 200 and at least 10 participants per item (Boateng et al., 2018; Worthington and Whittaker, 2006). The top age range limit was increased from 50 to 70 years based on feedback in Study 1 from people over 50 years old who felt their participation was important but were previously excluded. Participants were offered the chance to enter a draw to receive one of four $50 supermarket vouchers.

#### Measures and procedure

Participants completed the 12-item CANS resulting from Study 1. Similar to Study 1, participants completed the RANDS, AUDIT and GF. Measures were completed through an online survey hosted by Qualtrics, as in Study 1.

#### Data analysis

A CFA will be run to test the dimensionality of the CANS. It is hypothesised that the same three-factor structure extracted in Study 1 will be shown in the new sample. Internal consistency and validity of the CANS will be examined using the same process as in Study 1 that is, Cronbach’s alpha values for each CANS subscale and the total score (internal consistency), correlation of CANS with the RANDS (criterion validity) and correlation of CANS with AUDIT and GF (construct validity).

### Results

#### Confirmatory factor analysis (CFA)

The three-factor solution shown in Study 1, corresponding to a *Threat to Fun, Self* and *Connection* was tested using JAMOVI software version 2. We assessed the adequacy of the model according to the following fit indices, as recommended by Xia and Yang (2019): (1) root mean square error of approximation (RMSEA; Steiger, 1990) at or less than 0.06 to indicate a ‘reasonable’ model data fit; (2) the Comparative Fit Index (CFI; Bentler, 1990) and Tucker–Lewis index (TLI; Tucker and Lewis, 1973) at or above 0.95 (Hu and Bentler, 1999); (3) the standardised root mean square residual (SRMR; Bentler, 1990) less than 0.08, indicating acceptable model fit (Hu and Bentler, 1999). Note that a significant chi-square test statistic was not run as this is not considered useful at sample sizes over 200 (Xia and Yang, 2019).

The distribution for each item was examined. Most individual items did not meet thresholds of adequate normality as their absolute standardised skew value was larger than 1.96 (Field, 2017) and visual inspection of histograms showed skewness across most items. As the assumption of normality could not be met, the weighted least square estimation method was used for the CFA (Li, 2016). An examination of inter-item correlations shows all correlation values between 0.15 and 0.5, indicating items show a good balance of correlating well but not too high and thus repetitive in measuring the intended construct (Clark and Watson, 1995). Overall, the three-factor CFA model showed a close fit to the data with CFI = 0.987 and TLI = 0.983, and RMSEA = 0.042 (95% CI [0.025–0.057]) and SRMR = 0.054 (See Supplemental Table 6 for individual standardised betas and statistics for items in each factor.

#### Internal consistency

The 12-item version of the CANS was examined for internal consistency. Cronbach alpha of 0.842 for all 12 items is considered ‘good’ (Hair et al., 2018), supporting the use of a total score. The three subscales of the CANS also demonstrated acceptable internal consistency through adequate Cronbach alphas (*Threat to Fun* α = 0.830, *Threat to Self* α = 0.761 and *Threat to Connection* α = 0.835). All subscales were significantly (*ps* < 0.001) and positively correlated with each other. However, no subscales showed high correlation (all alphas < 0.8), indicating good discriminative validity between subscales.

#### Validity and correlations

A correlation matrix was examined for total CANS scores, CANS subscales, RANDS score, GF and AUDIT scores (Table 3). As in Study 1, because all variables were significantly skewed, Spearman Rank Correlations were used. As predicted, all CANS subscales were significantly correlated with the RANDS, AUDIT and GF. However, the magnitude of the correlations differed between the subscales, with the *Threat to Self* subscale showing weaker correlations than the *Threat to Self* and *Threat to Connection* subscales. The correlations with the AUDIT and GF were similar across the three subscales.

**Table 3.** Spearman rank correlation coefficients (Spearman’s rho) between variables in Study 2.

	1.	2.	3.	4.	5.	6.	7.	8.	9.	10.	11.
1. CANS Total (*n* = 389)	–										
2. CANS Threat to fun	0.748***	–									
3. CANS Threat to self	0.656***	0.185***	–								
4. CANS Threat to connection	0.828***	0.552***	0.322***	–							
5. RANDS (*n* = 383)	0.677***	0.734***	0.195***	0.607***	–						
6. AUDIT Total (*n* = 385)	0.542***	0.419***	0.380***	0.429***	0.506***	–					
7. AUDIT Consumption	0.409***	0.347***	0.252***	0.335***	0.437***	0.869***	–				
8. AUDIT Dependence	0.461***	0.341***	0.351***	0.341***	0.415***	0.770***	0.563***	–			
9. AUDIT Alcohol-harm	0.524***	0.374***	0.412***	0.402***	0.427***	0.832***	0.503***	0.629***	–		
10. GF (*n* = 418)	0.386***	0.318***	0.254***	0.288***	0.427***	0.807***	0.886***	0.550***	0.502***	–	
11. Age (*n* = 389)	−0.251***	−0.163**	−0.079	−0.330***	−0.194***	−0.192***	−0.129*	−0.148**	−0.179***	−0.111*	–
12. Gender (*n* = 380)	−0.129*	−0.123*	−0.087	−0.096	−0.144**	−0.251***	−0.258***	−0.150**	−0.175***	−0.292***	−0.033

All values are Spearman’s Rho except for correlations with gender which are point-biserial correlations (male = 0, female = 1). Note differing sample sizes were due to participants exiting before completing the survey.

Age: participant’s age in years; CANS: Cheers Attitudes Towards Non-drinkers Scale total score; RANDS: Regan Attitudes to Non-drinkers total score; AUDIT: Alcohol Use Disorders Identification Test (AUDIT) total score; GF: graduated frequency of total alcohol consumption per year.

* *p* < 0.05. **p < 0.01. ***p < 0.001.

As evidence of criterion (concurrent) validity, the total CANS score showed a strong, significant positive correlation with the RANDS. In evidence of construct (convergent) validity, total CANS scores showed significant positive correlations with AUDIT total, AUDIT subscale scores and GF. The correlation with AUDIT total was strong, compared to a moderate correlation with GF. Again, using Fisher’s *r*-to-*z* transformations, the AUDIT Consumption subscale was shown to be significantly more related to the *Threat to Connection* subscale, and the *Threat to Self* subscale was significantly more related to the AUDIT Alcohol-harm subscale compared to the AUDIT Consumption subscale.

Table 3 also shows that age was significantly correlated with most variables. However, Spearman’s rho shows these correlations were weak to moderate in strength. As in Study 1, point-biserial correlation with gender was performed on all variables, showing significant negative correlations with all variables except the threat to self and *Threat to Connection* subscales, indicating males, on average, had higher scores on total CANS score, the threat to fun subscale, RANDS, AUDIT and GF.

### Discussion

The two studies reported here provide initial psychometric support for the Cheers Attitudes Towards Non-drinkers Scale (CANS), a self-report measure of attitudes towards non-drinkers. In Study 1, 29 items were created based on the group interviews with Australian drinkers (as presented in Cheers et al., 2021) and through EFA, a 12-item scale emerged with three factors (i.e. subscales). These factors corresponded with the *triple threat theory* presented by Cheers et al. (2021), showing drinkers’ attitudes towards non-drinkers are grounded in *threats to fun, self and connection*. Study 2 presented a CFA in a new sample of drinkers and showed the three-factor structure to hold and the scale to show good internal consistency and validity.

The 12-item CANS assessed attitudes towards non-drinkers and showed promising psychometric properties across two large samples of drinkers. The CANS also showed evidence of criterion validity through the expected significant and positive correlation with the Regan Attitudes towards Non-drinkers Scale (RANDS), the other existing scale of this construct. The subscales also showed good internal consistency and correlated adequately with each other. As such, these studies contribute to the growing evidence indicating that stigma towards non-drinkers is grounded in the threats non-drinkers are perceived to present to both the values of connection and fun (as shown by Regan and Morrison, 2011), but also the threat of self-reflection of an individual’s own drinking. As demonstrated in the foundational qualitative work (Cheers et al., 2021), part of this stems from the scepticism of the ability to have a good time without alcohol (*Threat to Fun*) and a lack of confidence in the ability to develop strong connections without the social inhibition provided by alcohol (*Threat to Connection*). Further, drinkers may fear that their intoxicated behaviour might be brought against them after the drinking occasion, as well as show a reluctance to question their own drinking and the negative consequences of their drinking (*Threat to Self*).

Supporting existing research (Regan and Morrison, 2013, 2017; Young et al., 2016; Zimmermann and Sieverding, 2010, 2011), we identified that more negative attitudes towards non-drinkers (high scores on CANS) were related to higher alcohol consumption (yearly total drinks and AUDIT). This relationship, consistently shown by the RANDS (Regan and Morrison, 2013, 2017), provides evidence of construct validity for the CANS. Interestingly, the relationship between alcohol consumption and attitudes towards non-drinkers was shown to be weaker for the graduated frequency measure of total consumption compared to the AUDIT. Further, the CANS Threat to Self was more strongly associated with the AUDIT Alcohol-related Harms than the Consumption subscale. Considering that AUDIT is a measure of alcohol-related harm, hazardous consumption and possible dependence (Saunders et al., 1993), this may suggest that stigmatising attitudes towards non-drinkers are more strongly related to risky or harmful drinking rather than just the amount of alcohol consumed. As this type of harmful consumption is more likely to be of concern for a drinker, the results of these two studies give further support to the theory that the stigmatising of the non-drinker is a defence reaction to a threat to the ego, that is, that the presence of non-drinkers forces an unwanted reflection relating to their own risky drinking patterns that drinkers stigmatise to avoid (Cheers et al., 2021).

The CANS measure allows an operationalisation of the *Triple Threat Theory*, and through the three subscales, it gives the ability to examine differences in the impact of the three primary threats non-drinkers are perceived to pose. All threat subscales were shown to have expected significant correlations with the alcohol consumption measures (AUDIT and GF). Notably, the *Threat to Self* subscale showed a weaker correlation with the RANDS compared to the *Threat to Fun* and *Threat to Connection* subscales. As the RANDS was constructed to measure perceived ‘social costs’ of non-drinking (Regan and Morrison, 2011), stronger correlations with the *Threat to Fun* and *Threat to Connection* factors are expected. This pattern of difference between the subscales further supports the utility of the three subscales of the CANS. The CANS allows this *Threat to Self* factor to be measured and, as such, is an important contribution to our theoretical understanding of what drives the stigma towards non-drinkers.

The findings of Studies 1 and 2 provide foundational evidence for the CANS as a valid and reliable new measure that warrants further examination. It is hoped that future studies will explore the utility of the CANS within more representative and larger samples to gain further insight into the stigma directed towards non-drinkers, an increasing population in Australia and other high-income countries (AIHW, 2020; McCabe et al., 2021). The next steps in CANS development should include examination of test-retest reliability and further research in more heterogenous samples, for example, samples with a wider range of education, gender and rurality.

The current studies offer a new conceptual framework to understand what drives stigma towards non-drinkers and present a short, reliable measure that can establish how the stigma may be influenced by individual differences (e.g. age, gender and personality dimensions). As such, this research can inform the growing number of public health campaigns that aim to reduce alcohol-related harm by increasing community acceptance of non-drinking (e.g. *Don’t Judge Me* Campaign, Sir David Martin Foundation, 2019). For example, supporting Peralta (2007), across both studies, men were shown to have more negative attitudes towards non-drinkers (higher CANS scores), suggesting changing male attitudes should be a critical focus of campaigns.

In a new contribution to the field, the CANS allows specific components of the stigma towards non-drinkers (e.g. Threat to Fun, Connection and Self) to be measured and inform more targeted strategies. For example, the CANS allows the *Threat to Self* component of stigma to be measured for the first time. This may inform a new type of campaign that aims to encourage drinkers to understand that their stigmatising of non-drinkers may be a deflection from their own issues with drinking and, through this awareness, lead to a reduction in their stigmatising behaviour. Finally, the CANS could be used to evaluate the success of these campaigns in reducing stigmatising attitudes.

While the negative experiences of non-drinkers are well understood, the factors that drive drinkers to stigmatise non-drinkers have been less examined. The studies presented here endorse that the stigmatisation of non-drinkers is underpinned by the threat drinkers perceive non-drinkers pose to fun, connection and self. Taken together, these studies also present the CANS as a short, reliable and valid measure that has great potential to promote a better understanding of this stigma and inform strategies that might work to encourage a more accepting environment for people who choose not to drink. As such, it is hoped the CANS will contribute to a growing body of research that aims to better understand, and therefore better challenge, the stigma experienced by non-drinkers and promote greater acceptance of healthier drinking choices.

## Supplemental Material

sj-docx-8-hpq-10.1177_13591053231220519 – Supplemental material for Development and Validation of the Cheers Attitudes towards Non-drinkers Scale (CANS) Supplemental material, sj-docx-8-hpq-10.1177_13591053231220519 for Development and Validation of the Cheers Attitudes towards Non-drinkers Scale (CANS) by Christopher Cheers, Amy Pennay, Xochitl de la Piedad Garcia and Sarah Callinan in Journal of Health Psychology

sj-docx-1-hpq-10.1177_13591053231220519 – Supplemental material for Development and Validation of the Cheers Attitudes towards Non-drinkers Scale (CANS) Supplemental material, sj-docx-1-hpq-10.1177_13591053231220519 for Development and Validation of the Cheers Attitudes towards Non-drinkers Scale (CANS) by Christopher Cheers, Amy Pennay, Xochitl de la Piedad Garcia and Sarah Callinan in Journal of Health Psychology This article is distributed under the terms of the Creative Commons Attribution 4.0 License ( https://creativecommons.org/licenses/by/4.0/) which permits any use, reproduction and distribution of the work without further permission provided the original work is attributed as specified on the SAGE and Open Access pages ( https://us.sagepub.com/en-us/nam/open-access-at-sage).

sj-omv-2-hpq-10.1177_13591053231220519 – Supplemental material for Development and Validation of the Cheers Attitudes towards Non-drinkers Scale (CANS) Supplemental material, sj-omv-2-hpq-10.1177_13591053231220519 for Development and Validation of the Cheers Attitudes towards Non-drinkers Scale (CANS) by Christopher Cheers, Amy Pennay, Xochitl de la Piedad Garcia and Sarah Callinan in Journal of Health Psychology This article is distributed under the terms of the Creative Commons Attribution 4.0 License ( https://creativecommons.org/licenses/by/4.0/) which permits any use, reproduction and distribution of the work without further permission provided the original work is attributed as specified on the SAGE and Open Access pages ( https://us.sagepub.com/en-us/nam/open-access-at-sage).

sj-omv-5-hpq-10.1177_13591053231220519 – Supplemental material for Development and Validation of the Cheers Attitudes towards Non-drinkers Scale (CANS) Supplemental material, sj-omv-5-hpq-10.1177_13591053231220519 for Development and Validation of the Cheers Attitudes towards Non-drinkers Scale (CANS) by Christopher Cheers, Amy Pennay, Xochitl de la Piedad Garcia and Sarah Callinan in Journal of Health Psychology This article is distributed under the terms of the Creative Commons Attribution 4.0 License ( https://creativecommons.org/licenses/by/4.0/) which permits any use, reproduction and distribution of the work without further permission provided the original work is attributed as specified on the SAGE and Open Access pages ( https://us.sagepub.com/en-us/nam/open-access-at-sage).

sj-pdf-3-hpq-10.1177_13591053231220519 – Supplemental material for Development and Validation of the Cheers Attitudes towards Non-drinkers Scale (CANS) Supplemental material, sj-pdf-3-hpq-10.1177_13591053231220519 for Development and Validation of the Cheers Attitudes towards Non-drinkers Scale (CANS) by Christopher Cheers, Amy Pennay, Xochitl de la Piedad Garcia and Sarah Callinan in Journal of Health Psychology This article is distributed under the terms of the Creative Commons Attribution 4.0 License ( https://creativecommons.org/licenses/by/4.0/) which permits any use, reproduction and distribution of the work without further permission provided the original work is attributed as specified on the SAGE and Open Access pages ( https://us.sagepub.com/en-us/nam/open-access-at-sage).

sj-pdf-4-hpq-10.1177_13591053231220519 – Supplemental material for Development and Validation of the Cheers Attitudes towards Non-drinkers Scale (CANS) Supplemental material, sj-pdf-4-hpq-10.1177_13591053231220519 for Development and Validation of the Cheers Attitudes towards Non-drinkers Scale (CANS) by Christopher Cheers, Amy Pennay, Xochitl de la Piedad Garcia and Sarah Callinan in Journal of Health Psychology This article is distributed under the terms of the Creative Commons Attribution 4.0 License ( https://creativecommons.org/licenses/by/4.0/) which permits any use, reproduction and distribution of the work without further permission provided the original work is attributed as specified on the SAGE and Open Access pages ( https://us.sagepub.com/en-us/nam/open-access-at-sage).

sj-pdf-6-hpq-10.1177_13591053231220519 – Supplemental material for Development and Validation of the Cheers Attitudes towards Non-drinkers Scale (CANS) Supplemental material, sj-pdf-6-hpq-10.1177_13591053231220519 for Development and Validation of the Cheers Attitudes towards Non-drinkers Scale (CANS) by Christopher Cheers, Amy Pennay, Xochitl de la Piedad Garcia and Sarah Callinan in Journal of Health Psychology This article is distributed under the terms of the Creative Commons Attribution 4.0 License ( https://creativecommons.org/licenses/by/4.0/) which permits any use, reproduction and distribution of the work without further permission provided the original work is attributed as specified on the SAGE and Open Access pages ( https://us.sagepub.com/en-us/nam/open-access-at-sage).

sj-pdf-7-hpq-10.1177_13591053231220519 – Supplemental material for Development and Validation of the Cheers Attitudes towards Non-drinkers Scale (CANS) Supplemental material, sj-pdf-7-hpq-10.1177_13591053231220519 for Development and Validation of the Cheers Attitudes towards Non-drinkers Scale (CANS) by Christopher Cheers, Amy Pennay, Xochitl de la Piedad Garcia and Sarah Callinan in Journal of Health Psychology This article is distributed under the terms of the Creative Commons Attribution 4.0 License ( https://creativecommons.org/licenses/by/4.0/) which permits any use, reproduction and distribution of the work without further permission provided the original work is attributed as specified on the SAGE and Open Access pages ( https://us.sagepub.com/en-us/nam/open-access-at-sage).
