# Supplementary material for: Development and Validation of the Cheers Attitudes towards Non-drinkers Scale (CANS)
Source: J Health Psychol. 2024 Jan 29;29(10):1101–14. doi: 10.1177/13591053231220519 (PMC11344955; doi:10.1177/13591053231220519)

**Additional Data Analysis - Supplementary Material**

**Supplementary Table 1**

*Participant Demographics of the Final Sample in both EFA and CFA studies.*

|  |  | Study 1 | |  | Study 2 | |
| --- | --- | --- | --- | --- | --- | --- |
| Total Sample |  | 426 |  |  | 389 |  |
|  |  | *n* | *%* |  | *n* | *%* |
| Gender |  |  |  |  |  |  |
| Female (including transgender women) |  | 225 | 52.8 |  | 211 | 54.2 |
| Male (including transgender men) |  | 189 | 44.4 |  | 169 | 43.4 |
| Non-binary, other specified |  | 9 | 2.8 |  | 7 | 1.8 |
| Prefer not to say |  | 3 | .7 |  | 2 | .5 |
| Age (years) |  |  |  |  |  |  |
| 18-24 |  | 53 | 12.4 |  | 35 | 9.0 |
| 25-34 |  | 101 | 23.7 |  | 132 | 33.9 |
| 35-49 |  | 254 | 59.6 |  | 126 | 32.4 |
| 50-70 |  | 18 | 4.2 |  | 96 | 24.7 |
| Education |  |  |  |  |  |  |
| Year 11 or below |  | 12 | 2.8 |  | 10 | 2.6 |
| Year 12 or equivalent |  | 65 | 15.3 |  | 47 | 12.1 |
| Diploma or equivalent |  | 87 | 20.4 |  | 69 | 17.7 |
| Bachelor’s degree or higher |  | 262 | 61.3 |  | 254 | 65.3 |
| Did not indicate |  | 0 | 0 |  | 9 | 2.3 |
| Student status |  |  |  |  |  |  |
| Current Student |  | 118 | 27.7 |  | 72 | 18.5 |
| Not current student |  | 308 | 72.3 |  | 308 | 79.1 |
| Did not indicate |  | 0 | 0 |  | 9 | 2.4 |
| Location |  |  |  |  |  |  |
| Victoria |  | 182 | 42.7 |  | 194 | 49.9 |
| New South Wales |  | 89 | 20.9 |  | 102 | 26.2 |
| Queensland |  | 53 | 12.4 |  | 33 | 8.5 |
| Western Australia |  | 33 | 7.7 |  | 25 | 6.4 |
| South Australia |  | 35 | 8.2 |  | 19 | 4.9 |
| Tasmania |  | 17 | 4.0 |  | 4 | 1.0 |
| Australian Capital Territory |  | 12 | 2.8 |  | 9 | 2.3 |
| Northern Territory |  | 5 | 1.2 |  | 3 | .8 |
| Remoteness |  |  |  |  |  |  |
| Major City |  | 323 | 75.8 |  | 287 | 73.8 |
| Inner Regional |  | 100 | 23.5 |  | 90 | 23.1 |
| Remote |  | 3 | .7 |  | 2 | .5 |
| Not indicated |  | 0 | 0 |  | 10 | 2.6 |

**Study 1: Item Development**

Expert feedback process

*Note: The following instructions were provided to the panel of experts to establish expert feedback on the initial items.*

Thank you for taking the time to complete this assessment of the suitability of a newly developed survey that aims to measure attitudes toward non-drinkers in Australia. This information will be used to identify areas of improvement in the newly developed items (e.g., re-wording items, removing items).

The items included are based on a theoretical framework that has been developed through focus groups which examined drinker attitudes toward non-drinkers. The attitudes presented by these focus groups were best understood as based on the following three themes:

(1) drinkers described non-drinkers as posing a ***threat to fun****,* with non-drinkers described as a judgmental ‘sober eye’ disrupting the hedonistic environment created by alcohol,

(2) non-drinkers were characterised as difficult to initiate and maintain ***social connection*** with, and

(3) drinkers described non-drinkers as forcing a ***reflection on the problematic aspects of their own drinking***.

On the following pages, for each proposed survey item you will be asked to:

1. Indicate which theme/s you believe the item is measuring:

**T**=Threat to fun, **S**=Social Connection, **R**=Reflection on the problematic aspects of their own drinking).

1. Rate the relevance of each item by choosing an option that best represents your response

**R**=relevant, **NR**=not relevant, **M**=relevant if Modified

When assessing the relevance of an item, you may consider the following: item clarity, item difficulty, and the suitability of each item to the construct.

Indicating **NR** ‘not relevant’ indicates that you consider the item should be discarded. Please explain why you think the item is 'not relevant'

Indicating **M** ‘relevant if modified’ requires you to explain what concerns you may have about the item and how you would revise it accordingly

Please also keep the items in mind as you are progressing through as you will be asked at the end whether the items comprehensively reflect the construct. 

When rating each item keep in mind the survey will start with the following statement:

“For the following questions, imagine you were in a social situation, like a party, where most people were drinking, and you met someone who said they were not drinking. This person will be referred to as a non-drinker.”

Participants will then be asked to rate each item from strongly disagree to strongly agree (a five-point Likert scale will be added *Strongly Disagree, Disagree, Neither, Agree, Strongly Agree*).

Please now rate each item.

*[Note: for the list of items presented please refer to the list in the “process of item removal or retention” section of this supplementary material]*

In your opinion, do you think that the combination of items presented throughout the survey comprehensively measure the construct in relation to its factors (threat to fun, social connection, reflection on drinking)? Please provide any comments around the items specifically or in general (if you wish to respond with feedback below)

If you have any final comments and feedback to provide around the items and concepts, please provide this in the space provided below.

Potential Participants Item Feedback Process

*Note: The following instructions were presented to potential participants before the initial list of questions to guide feedback.*

For the following questions, imagine you were in a social situation, like a party, where most people were drinking, and you met someone who said they were not drinking. This person will be referred to as a non-drinker.

Imagine you have been asked to rate each item from strongly disagree to strongly agree (a five-point Likert scale will be added *Strongly Disagree, Disagree, Neither, Agree, Strongly Agree*). Please tick the box if you have no issues with the item. If you think the item needs removal or to be changed please comment. Some reasons for item change may be that the item is: difficult to answer, seems unrelated to the topic, or is hard to understand.

*[Note: for the list of items presented please refer to the list in the “process of item removal or retention” section of this supplementary material]*

**Study 1: Process of item removal or retention**

*Note: Below summarises whether each of the initial items was removed, modified, or retained for the exploratory factor analysis, based on feedback from both experts and potential participants.*

**Threat to Fun**

1. Non-drinkers are choosing to have less fun at the party. - Removed as *lacked clarity*.
2. Non-drinkers will be a buzzkill for other people at the party. - Established as *relevant if modified and thus amended to* “Non-drinkers will spoil the fun for other people at the party.”
3. Non-drinkers would have more fun if they were drinking. - Removed as *not relevant*
4. Non-drinkers would make it harder for me to relax and have fun. - *Established as relevant if modified and thus amended to remove* “and have fun.”
5. Non-drinkers spoil the fun. – item retained
6. You have to be careful around non-drinkers at a party, as they will remember things you may do when you’re drunk. - Removed as *lacked clarity*.
7. I am conscious not to have too much fun around non-drinkers at parties - *Established as relevant if modified and thus amended to* “I am conscious of not having too much fun around non-drinkers at parties”)
8. You have to be aware of your behaviour around non-drinkers at parties. - Removed as *lacked clarity* and *not relevant.*
9. I would have less fun with a non-drinker at a party. – item retained
10. I feel like the party gets more fun when the non-drinker leaves. – item retained
11. I feel more comfortable with someone who is drinking at a party. – item retained
12. Non-drinkers make a party uncomfortable for people - Removed as *lacked clarity*
13. It’s easier to have fun with drinkers. - *Established as relevant if modified and thus amended to* “It’s easier to enjoy myself with drinkers.”
14. I sometimes wonder why non-drinkers are at parties where people are drinking and having fun. - Removed as *lacked clarity* and *not relevant.*

**Threat to connection**

1. It’s harder to connect to non-drinkers. - Removed as *lacked clarity*.
2. It can be difficult to connect with non-drinkers. – item retained
3. It can be difficult to talk to non-drinkers. - Removed as *lacked clarity*.
4. When first meeting a non-drinker it can be challenging to chat. - Removed as *lacked clarity*.
5. I believe the bonds made over drinks are the strongest ones. – item retained
6. It’s easier to form intimate connections with drinkers - Established as *relevant if modified and thus amended to* “Drinking makes it easier to make new friends.”
7. It takes more effort to talk to non-drinkers. – item retained
8. It would be difficult to date a non-drinker – removed as referring to dating was seen as not relevant and overcomplicates the item.
9. Its easier to get to know a drinker. - Established as *relevant if modified and thus amended to* “Getting to know someone is easier if they are drinking.”
10. I would hope a first date would be a drinker – removed as referring to dating was seen as not relevant and overcomplicates the item.
11. If I went on a first date with a non-drinker it would be challenging. – removed as referring to dating was seen as not relevant and overcomplicates the item.
12. Drinking makes it easier to form a deeper connection with people. – Removed as *lacked clarity*.
13. You can form close connection with non-drinkers but it would take longer. – Removed as *lacked clarity*.
14. I feel non-drinkers are not as authentic. - Established as *relevant if modified and thus amended to* “Non-drinkers can hide who they really are.”
15. I feel like non-drinkers are wearing a mask – Removed as *lacked clarity*.
16. Drinking helps you reveal yourself to others – Removed as *lacked clarity*.
17. Non-drinkers are more guarded socially. – item retained
18. I think non-drinkers are hiding something. - Established as *relevant if modified and thus amended to* “I think non-drinkers are hiding who they are.”
19. Non-drinkers are less sociable. – Removed as *lacked clarity*.
20. Drinking helps bond people. – Removed as *lacked clarity*.
21. Alcohol helps people come together. – Removed as *lacked clarity* and *not relevant*.
22. Alcohol provides a short-cut to social bonds - Established as *relevant if modified and thus amended to* “Alcohol provides a short-cut to social bonding.”)
23. It’s hard to get to know non-drinkers. – item retained
24. If my partner stopped drinking, that would be challenging for the relationship – removed as referring to a partner was seen as not relevant and overcomplicated the item.
25. If my partner did not drink, that would cause friction in our relationship – removed as referring to a partner was seen as not relevant and overcomplicated the item.
26. Drinking creates intimacy between people. – Removed as *lacked clarity*.

**Threat to self**

1. I sometimes wish I could be a non-drinker. – Established as *relevant if modified and thus amended to* “I sometimes wish I could drink less.”
2. If I was a non-drinker, I would be healthier. – item retained
3. If I was a non-drinker, I would be happier – Removed as *not relevant*.
4. Non-drinkers make me feel guilty - Established as *relevant if modified and thus amended to* “Non-drinkers make me feel guilty about my drinking.”
5. Non-drinkers make me uncomfortable about my drinking. – item retained
6. Non-drinkers make me think about my drinking choices – Removed as *not relevant*.
7. Non-drinkers are making a choice I wish I could make. – item retained
8. Non-drinkers make me confront issues with my own drinking – Removed as *lacked clarity*.
9. I can feel resentful toward non-drinkers – Removed as *not relevant as wording too strong*.
10. I feel threatened by non-drinkers. – Removed as *not relevant* *as wording too strong*.
11. Non-drinkers hold a mirror up to my own drinking. – Removed as *lacked clarity*.
12. Non-drinkers make me think about my own drinking. – item retained
13. I sometimes want non-drinkers to drink to make me more comfortable. – item retained
14. Non-drinkers make it difficult to justify my drinking. – Removed as *lacked clarity*.
15. I can feel threatened by non-drinkers. – Removed as *not relevant* *as wording too strong*.
16. It can feel like non-drinkers are judging me. – Established as *relevant if modified and thus amended to* “I feel like non-drinkers are judging me.”
17. You have to be cautious around non-drinkers. – Removed as *lacked clarity*.
18. Non-drinkers can make me feel self-aware. – item retained
19. Non-drinkers ruin the illusion of fun. – Removed as *lacked clarity*.
20. Non-drinkers are making the healthier choice. – Established as *relevant if modified and thus amended to* “Non-drinkers are making a healthier choice than me.”)
21. Non-drinkers are healthier. – Removed as *lacked clarity*.
22. You should watch yourself around non-drinkers at parties. – item retained

**Study 1: Item List.**

*Note: The following includes the instructions and 29 items that were presented to participants in Study 1.*

We are interested in your attitudes toward non-drinkers. In the items below when we refer to a non-drinker, we mean a person in a social situation, like a party, where most people are drinking, and they are not.

Based on this, indicate for each of the items below whether you strongly disagree to strongly agree.

1. I believe the bonds made over drinks are the strongest ones.
2. It can be difficult to connect with non-drinkers.
3. I think non-drinkers are hiding who they are.
4. It’s hard to get to know non-drinkers.
5. Non-drinkers are more guarded socially.
6. Alcohol provides a short-cut to social bonding.
7. It takes more effort to talk to non-drinkers.
8. Getting to know someone is easier if they are drinking.
9. Drinking makes it easier to make new friends.
10. Non-drinkers can hide who they really are.
11. You should watch yourself around non-drinkers at parties.
12. Non-drinkers make it harder for me to relax.
13. Non-drinkers spoil the fun.
14. I am conscious of not having too much fun around non-drinkers at parties.
15. I would have less fun with a non-drinker at a party.
16. It’s easier to enjoy myself with drinkers.
17. I feel like the party gets more fun when non-drinkers leave.
18. I feel more comfortable with someone who is drinking at a party.
19. Non-drinkers will spoil the fun for other people at the party.
20. I sometimes wish I could drink less.
21. Non-drinkers make me feel guilty about my drinking.
22. Non-drinkers make me think about my own drinking.
23. I feel like non-drinkers are judging me.
24. Non-drinkers are making a healthier choice than me.
25. I sometimes want non-drinkers to drink to make me more comfortable.
26. Non-drinkers make me uncomfortable about my drinking.
27. If I was a non-drinker, I would be healthier.
28. Non-drinkers are making a choice I wish I could make.
29. Non-drinkers can make me feel very self-aware.

**Study 1: Exploratory Factor Analysis**

**Supplementary Figure 1.**

*Initial Scree Plot*


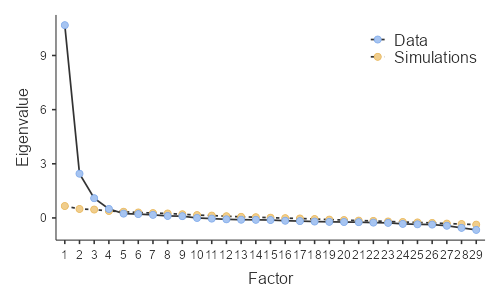


**EFA Steps**

*Note: The following summarises the steps that were involved in the EFA and the reasons each item was removed.*

**Supplementary Table 2.**

*EFA run all 29 items (Step One)*

|  | | **Factor** | | | | | |  | |
| --- | --- | --- | --- | --- | --- | --- | --- | --- | --- |
|  | | **1** | | **2** | | **3** | | **Uniqueness** | |
| CAN29 |  | 0.334 |  |  |  | 0.570 |  | 0.463 |  |
| CAN28 |  |  |  |  |  | 0.660 |  | 0.574 |  |
| CAN27 |  |  |  |  |  | 0.704 |  | 0.483 |  |
| CAN26 |  | 0.542 |  |  |  | 0.451 |  | 0.389 |  |
| CAN25 |  | 0.623 |  |  |  |  |  | 0.514 |  |
| CAN24 |  |  |  |  |  | 0.735 |  | 0.445 |  |
| CAN23 |  | 0.628 |  |  |  |  |  | 0.506 |  |
| CAN22 |  |  |  |  |  | 0.679 |  | 0.501 |  |
| CAN21 |  | 0.562 |  |  |  | 0.454 |  | 0.433 |  |
| CAN20 |  |  |  |  |  | 0.664 |  | 0.475 |  |
| CAN19 |  | 0.781 |  |  |  |  |  | 0.387 |  |
| CAN18 |  | 0.373 |  | 0.471 |  |  |  | 0.405 |  |
| CAN17 |  | 0.614 |  |  |  |  |  | 0.399 |  |
| CAN16 |  |  |  | 0.587 |  |  |  | 0.385 |  |
| CAN15 |  | 0.463 |  | 0.385 |  |  |  | 0.426 |  |
| CAN14 |  | 0.581 |  |  |  |  |  | 0.568 |  |
| CAN13 |  | 0.752 |  |  |  |  |  | 0.366 |  |
| CAN12 |  | 0.641 |  |  |  |  |  | 0.441 |  |
| CAN11 |  | 0.646 |  |  |  |  |  | 0.589 |  |
| CAN10 |  | 0.355 |  |  |  |  |  | 0.789 |  |
| CAN9 |  |  |  | 0.707 |  |  |  | 0.502 |  |
| CAN8 |  |  |  | 0.780 |  |  |  | 0.378 |  |
| CAN7 |  |  |  | 0.520 |  |  |  | 0.487 |  |
| CAN6 |  |  |  | 0.753 |  |  |  | 0.484 |  |
| CAN5 |  | 0.343 |  | 0.312 |  |  |  | 0.654 |  |
| CAN4 |  | 0.345 |  | 0.506 |  |  |  | 0.440 |  |
| CAN3 |  | 0.590 |  |  |  |  |  | 0.622 |  |
| CAN2 |  |  |  | 0.561 |  |  |  | 0.466 |  |
| CAN1 |  |  |  | 0.550 |  |  |  | 0.650 |  |

*NB. Uniqueness is 1 – communality e.g., a communality above .7 is equivalent to below .3.*

Item 10 was removed as communality below .3 (uniqueness above .7).

Items removed as cross-loading above .32: CAN 4, 5, 15 ,18, 26, 21, 29.

**Supplementary Table 3.**

*EFA on the remaining 21 items (Step 2)*

|  | | **Factor** | | | | | |  | |
| --- | --- | --- | --- | --- | --- | --- | --- | --- | --- |
|  | | **1** | | **2** | | **3** | | **Uniqueness** | |
| CAN28 |  |  |  |  |  | 0.674 |  | 0.565 |  |
| CAN27 |  |  |  |  |  | 0.758 |  | 0.424 |  |
| CAN24 |  |  |  |  |  | 0.782 |  | 0.392 |  |
| CAN23 |  | 0.580 |  |  |  |  |  | 0.558 |  |
| CAN22 |  |  |  |  |  | 0.606 |  | 0.591 |  |
| CAN20 |  |  |  |  |  | 0.672 |  | 0.463 |  |
| CAN19 |  | 0.828 |  |  |  |  |  | 0.366 |  |
| CAN17 |  | 0.698 |  |  |  |  |  | 0.382 |  |
| CAN14 |  | 0.603 |  |  |  |  |  | 0.586 |  |
| CAN13 |  | 0.848 |  |  |  |  |  | 0.311 |  |
| CAN12 |  | 0.692 |  |  |  |  |  | 0.430 |  |
| CAN9 |  |  |  | 0.737 |  |  |  | 0.458 |  |
| CAN8 |  |  |  | 0.808 |  |  |  | 0.351 |  |
| CAN7 |  | 0.317 |  | 0.473 |  |  |  | 0.496 |  |
| CAN6 |  |  |  | 0.758 |  |  |  | 0.470 |  |
| CAN3 |  | 0.594 |  |  |  |  |  | 0.635 |  |
| CAN2 |  | 0.301 |  | 0.504 |  |  |  | 0.482 |  |
| CAN1 |  |  |  | 0.511 |  |  |  | 0.657 |  |
| CAN25 |  | 0.632 |  |  |  |  |  | 0.538 |  |
| CAN11 |  | 0.660 |  |  |  |  |  | 0.583 |  |
| CAN16 |  |  |  | 0.531 |  |  |  | 0.416 |  |
|  | | | | | | | | | |

 Items removed as cross-loading above .32: CAN 7, 2.

**Supplementary Table 4.**

*EFA on the remaining 19 items (Step 3)*

|  | | **Factor** | | | | | |  | |
| --- | --- | --- | --- | --- | --- | --- | --- | --- | --- |
|  | | **1** | | **2** | | **3** | | **Uniqueness** | |
| CAN13 |  | 0.849 |  |  |  |  |  | 0.311 |  |
| CAN19 |  | 0.827 |  |  |  |  |  | 0.364 |  |
| CAN17 |  | 0.704 |  |  |  |  |  | 0.377 |  |
| CAN12 |  | 0.700 |  |  |  |  |  | 0.439 |  |
| CAN11 |  | 0.656 |  |  |  |  |  | 0.576 |  |
| CAN25 |  | 0.641 |  |  |  |  |  | 0.546 |  |
| CAN14 |  | 0.599 |  |  |  |  |  | 0.584 |  |
| CAN3 |  | 0.598 |  |  |  |  |  | 0.633 |  |
| CAN23 |  | 0.588 |  |  |  |  |  | 0.559 |  |
| CAN24 |  |  |  | 0.779 |  |  |  | 0.394 |  |
| CAN27 |  |  |  | 0.753 |  |  |  | 0.426 |  |
| CAN28 |  |  |  | 0.683 |  |  |  | 0.559 |  |
| CAN20 |  |  |  | 0.675 |  |  |  | 0.463 |  |
| CAN22 |  |  |  | 0.596 |  |  |  | 0.592 |  |
| CAN8 |  |  |  |  |  | 0.781 |  | 0.357 |  |
| CAN6 |  |  |  |  |  | 0.761 |  | 0.452 |  |
| CAN9 |  |  |  |  |  | 0.738 |  | 0.442 |  |
| CAN16 |  | 0.315 |  |  |  | 0.527 |  | 0.406 |  |
| CAN1 |  |  |  |  |  | 0.456 |  | 0.684 |  |
|  | | | | | | | | | |

Items removed as cross-loading above .32: CAN 16.

Items deleted based on evaluation of meaningful contribution and repetition:

CAN20 (“I sometimes wish I could drink less”) was removed as showed inconsistencies with the rest of the factor as did not focus on an attitude about non-drinkers.

CAN23 (“I feel like non-drinkers are judging me”), CAN 3 (“I think non-drinkers are hiding who they are” and CAN11 (“You should watch yourself around non-drinkers at parties”) removed as do not meaningfully contribute to *threat to fun* factor nor align with the other items in that factor.

CAN17 (“I feel like the party gets more fun when non-drinkers leave”) and CAN19 (“Non-drinkers will spoil the fun for other people at the party”) removed as the content was repetitive of, and showed high correlation with, CAN13 (“Non-drinkers spoil the fun”).

**Supplementary Table 5.**

*Means, standard deviations and statistics for variables in Study One.*

|  | | Age | | RANDS | | AUDIT | | CANS | | GF | |
| --- | --- | --- | --- | --- | --- | --- | --- | --- | --- | --- | --- |
| N |  | 426 |  | 426 |  | 404 |  | 426 |  | 418 |  |
| Missing |  | 0 |  | 0 |  | 22 |  | 0 |  | 8 |  |
| Mean |  | 37.3 |  | 27.1 |  | 17.6 |  | 31.9 |  | 966 |  |
| Median |  | 39.0 |  | 27.0 |  | 16.0 |  | 32.0 |  | 420 |  |
| Standard deviation |  | 9.37 |  | 6.09 |  | 5.92 |  | 7.37 |  | 1345 |  |
| Minimum |  | 18.0 |  | 11.0 |  | 11.0 |  | 12.0 |  | 0.00 |  |
| Maximum |  | 50.0 |  | 55.0 |  | 42.0 |  | 51.0 |  | 7191 |  |
| Skewness |  | -0.499 |  | 0.442 |  | 1.29 |  | 0.0691 |  | 2.40 |  |
| Std. error skewness |  | 0.118 |  | 0.118 |  | 0.121 |  | 0.118 |  | 0.119 |  |
| Kurtosis |  | -0.818 |  | 0.473 |  | 1.56 |  | -0.251 |  | 6.30 |  |
| Std. error kurtosis |  | 0.236 |  | 0.236 |  | 0.242 |  | 0.236 |  | 0.238 |  |
| Shapiro-Wilk W |  | 0.933 |  | 0.982 |  | 0.879 |  | 0.994 |  | 0.698 |  |
| Shapiro-Wilk p |  | < .001 |  | < .001 |  | < .001 |  | 0.130 |  | < .001 |  |

*Note.* Age = Participant’s age in years, CANS = Cheers Attitude Toward Non-drinkers Scale total score, RANDS = Regan Attitudes to Non-drinkers total score, AUDIT = Alcohol Use Disorders Identification Test (AUDIT) total score, and GF = graduated frequency of total alcohol consumption per year.

**Study 2: Cheers Attitudes to Non-drinkers Scale (CANS)**

We are interested in your attitudes toward non-drinkers. In the items below when we refer to a non-drinker, we mean a person in a social situation, like a party, where most people are drinking, and they are not.

Based on this, indicate for each of the items below whether you strongly disagree to strongly agree.

1. Getting to know someone is easier if they are drinking.
2. Non-drinkers make it harder for me to relax.
3. Alcohol provides a shortcut to social bonding.
4. If I was a non-drinker, I would be healthier.
5. I sometimes want non-drinkers to drink to make me more comfortable.
6. Non-drinkers make me think about my own drinking.
7. Non-drinkers spoil the fun.
8. Drinking makes it easier to make new friends.
9. Non-drinkers are making a choice I wish I could make.
10. I am conscious of not having too much fun around non-drinkers at parties.
11. Non-drinkers are making a healthier choice than me.
12. I believe the bonds made over drinks are the strongest ones.

*Note for scoring:*

The CANS is a quantitative measure of stigma toward non-drinkers on three subscales: threat to fun, threat to self and threat to connection. Each item should be given a score from 1 (strongly disagree) to 5 (strongly agree). Scores on each item can be summed to give total CANS score. Items in each subscale can also be summed to give subscale scores according to the following:

Threat to fun: Items 2, 5, 7, 10

Threat to self: Items 4, 6, 9, 11

Threat to connection: Items 1, 3, 8, 12

**Supplementary Table 6.**

*Reliability, means, standard deviations and standardised betas of CFA of the [redacted] Attitude to Non-drinkers Scale (CANS)*

|  |  | CFA |  |
| --- | --- | --- | --- |
| Items | M | SD | β |
| **Factor 1: Threat to fun (α = .830)** |  |  |  |
| 1. Non-drinkers spoil the fun. | 1.74 | .802 | .734 |
| 1. Non-drinkers make it harder for me to relax. | 1.74 | .909 | .773 |
| 1. I am conscious of not having too much fun around non-drinkers at parties. | 1.96 | 1.07 | .731 |
| 1. I sometimes want non-drinkers to drink to make me more comfortable. | 1.94 | 1.10 | .771 |
| **Factor 2: Threat to self (α = .761)** |  |  |  |
| 1. Non-drinkers are making a healthier choice than me. | 3.83 | .969 | .635 |
| 1. If I was a non-drinker, I would be healthier. | 3.66 | 1.00 | .682 |
| 1. Non-drinkers are making a choice I wish I could make. | 2.61 | 1.06 | .703 |
| 1. Non-drinkers make me think about my own drinking. | 3.35 | 1.09 | .624 |
| **Factor 3: Threat to connection (α = .835)** |  |  |  |
| 1. Drinking makes it easier to make new friends. | 3.25 | 1.10 | .790 |
| 1. Alcohol provides a short-cut to social bonding. | 3.63 | .988 | .697 |
| 1. Getting to know someone is easier if they are drinking. | 3.02 | 1.12 | .812 |
| 1. I believe the bonds made over drinks are the strongest ones. | 2.00 | .907 | .677 |
|  | | | |

**Supplementary Table 7.**

*Means, standard deviations and statistics for Study Two variables.*

|  | | Age | | RANDS | | AUDIT | | CANS | | GF | |
| --- | --- | --- | --- | --- | --- | --- | --- | --- | --- | --- | --- |
| N |  | 389 |  | 383 |  | 385 |  | 389 |  | 389 |  |
| Missing |  | 0 |  | 6 |  | 4 |  | 0 |  | 0 |  |
| Mean |  | 39.8 |  | 25.3 |  | 8.21 |  | 32.7 |  | 783 |  |
| Median |  | 36.0 |  | 25.0 |  | 7.00 |  | 33.0 |  | 438 |  |
| Standard deviation |  | 13.5 |  | 8.14 |  | 5.78 |  | 7.35 |  | 1013 |  |
| Variance |  | 181 |  | 66.2 |  | 33.4 |  | 54.0 |  | 1.03e+6 |  |
| Range |  | 52.0 |  | 35.0 |  | 31.0 |  | 41.0 |  | 7656 |  |
| Minimum |  | 18.0 |  | 11.0 |  | 1.00 |  | 14.0 |  | 9.00 |  |
| Maximum |  | 70.0 |  | 46.0 |  | 32.0 |  | 55.0 |  | 7665 |  |
| Skewness |  | 0.580 |  | 0.340 |  | 1.19 |  | 0.127 |  | 2.66 |  |
| Std. error skewness |  | 0.124 |  | 0.125 |  | 0.124 |  | 0.124 |  | 0.124 |  |
| Kurtosis |  | -0.629 |  | -0.621 |  | 1.49 |  | -0.183 |  | 9.72 |  |
| Std. error kurtosis |  | 0.247 |  | 0.249 |  | 0.248 |  | 0.247 |  | 0.247 |  |
| Shapiro-Wilk W |  | 0.939 |  | 0.974 |  | 0.908 |  | 0.994 |  | 0.715 |  |
| Shapiro-Wilk p |  | < .001 |  | < .001 |  | < .001 |  | 0.151 |  | < .001 |  |

*Note.* Age = Participant’s age in years, CANS = Cheers Attitude Toward Non-drinkers Scale total score, RANDS = Regan Attitudes to Non-drinkers total score, AUDIT = Alcohol Use Disorders Identification Test (AUDIT) total score, and GF = graduated frequency of total alcohol consumption per year.

**Supplementary Table 8.**

*Statistics for individual items in the final CFA.*

|  | | | | | | | | **95% Confidence Intervals** | | | |  | | | | | |
| --- | --- | --- | --- | --- | --- | --- | --- | --- | --- | --- | --- | --- | --- | --- | --- | --- | --- |
| **Latent** | | **Observed** | | **Estimate** | | **SE** | | **Lower** | | **Upper** | | **β** | | **z** | | **p** | |
| Fun |  | CAN1 |  | 1.000 |  | 0.0000 |  | 1.000 |  | 1.000 |  | 0.734 |  |  |  |  |  |
|  |  | CAN2 |  | 1.178 |  | 0.0984 |  | 0.985 |  | 1.371 |  | 0.773 |  | 11.96 |  | < .001 |  |
|  |  | CAN3 |  | 1.328 |  | 0.1359 |  | 1.062 |  | 1.595 |  | 0.731 |  | 9.77 |  | < .001 |  |
|  |  | CAN4 |  | 1.442 |  | 0.1132 |  | 1.220 |  | 1.664 |  | 0.771 |  | 12.74 |  | < .001 |  |
| Ego |  | CAN5 |  | 1.000 |  | 0.0000 |  | 1.000 |  | 1.000 |  | 0.635 |  |  |  |  |  |
|  |  | CAN12 |  | 1.091 |  | 0.1385 |  | 0.819 |  | 1.362 |  | 0.672 |  | 7.87 |  | < .001 |  |
|  |  | CAN8 |  | 1.140 |  | 0.1649 |  | 0.817 |  | 1.463 |  | 0.678 |  | 6.91 |  | < .001 |  |
|  |  | CAN6 |  | 1.098 |  | 0.1518 |  | 0.800 |  | 1.395 |  | 0.617 |  | 7.23 |  | < .001 |  |
| Con |  | CAN9 |  | 1.000 |  | 0.0000 |  | 1.000 |  | 1.000 |  | 0.788 |  |  |  |  |  |
|  |  | CAN7 |  | 0.794 |  | 0.0558 |  | 0.684 |  | 0.903 |  | 0.702 |  | 14.22 |  | < .001 |  |
|  |  | CAN10 |  | 1.044 |  | 0.0560 |  | 0.935 |  | 1.154 |  | 0.814 |  | 18.64 |  | < .001 |  |
|  |  | CAN11 |  | 0.697 |  | 0.0597 |  | 0.580 |  | 0.814 |  | 0.679 |  | 11.68 |  | < .001 |  |
|  | | | | | | | | | | | | | | | | | |

**Supplementary Figure 2.**

*CFA Path Model with coefficients*


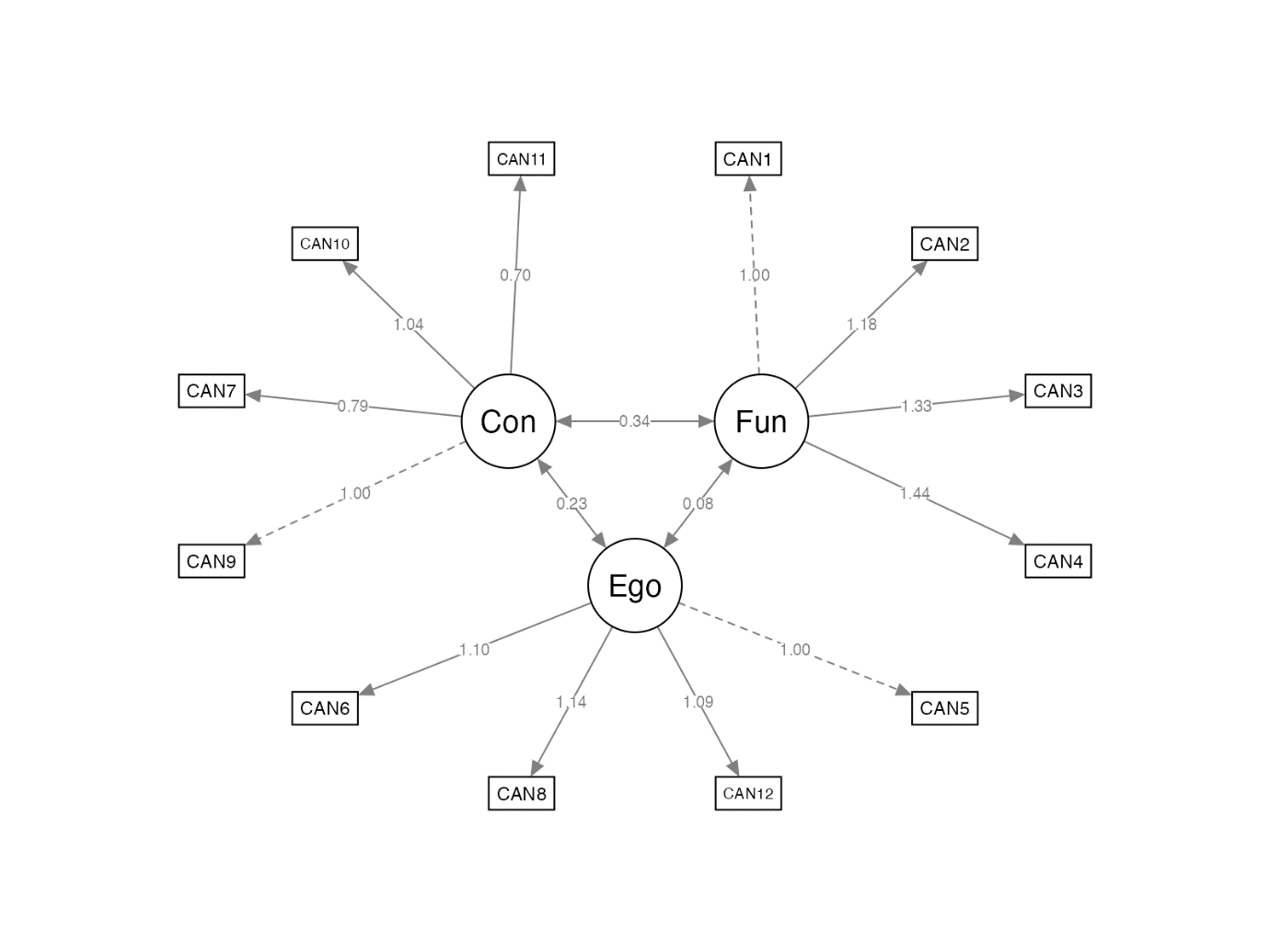

Supplement: sj-docx-8-hpq-10.1177_13591053231220519 – Supplemental material for Development and Validation of the Cheers Attitudes towards Non-drinkers Scale (CANS) [file sj-docx-8-hpq-10.1177_13591053231220519.docx]
