# Supplementary material for: Development and Validation of the Cheers Attitudes towards Non-drinkers Scale (CANS)
Source: J Health Psychol. 2024 Jan 29;29(10):1101–14. doi: 10.1177/13591053231220519 (PMC11344955; doi:10.1177/13591053231220519)
Supplement: sj-docx-1-hpq-10.1177_13591053231220519 – Supplemental material for Development and Validation of the Cheers Attitudes towards Non-drinkers Scale (CANS) [file sj-docx-1-hpq-10.1177_13591053231220519.docx]

**Explanatory Memo**

All analyses were carried out in JAMOVI Version 2.3.19.0 (<https://www.jamovi.org>). JAMOVI does not allow data files to be presented separately from output/analysis files. As such the data and output are presented in the JAMOVI files (.omv). The analysis and syntax are also presented in PDF form for reviewers who do not use JAMOVI. As JAMOVI does not create separate syntax files, the analysis for each study is presented in two forms, one showing just the output and the other with syntax and output.

*Filename*: StudyOneEFA.omv

*Explanation*: JAMOVI file which includes data and all analysis.

*Filename*: StudyOneSyntax.pdf

*Explanation*: JAMOVI does not create separate syntax files, however, the program does allow you to show the syntax before each analysis in the output. As such this file contains the syntax and all analysis for Study One.

*Filename*: StudyOneEFA.pdf

*Explanation*: JAMOVI output (without syntax) in PDF format. Shows all analysis for Study One. Please note that a filter has been used to ensure only drinkers (those participants who have consumed alcohol in the last 12 months are included analysis). Specifically, the Output for Study One presents:

1. Exploratory Factor Analysis Phase 1-3 (see Supplementary Material for annotated output explaining and justifying all decisions).
2. Final 12-item EFA solution.
3. Descriptive and frequencies of all relevant variables.
4. Correlation matrix showing Spearman’s Rho for all correlations including hypothesised tests of validity
5. Reliability Analysis for 12-item total scale and each individual subscale (e.g., threat to fun, connection, and self)

*Filename*: StudyTwoSyntax.pdf

*Explanation*: JAMOVI does not create separate syntax files, however, the program does allow you to show the syntax before each analysis in the output. As such this file contains the syntax and all analysis for Study Two.

*Filename*: StudyTwoCFA.pdf

*Explanation*: JAMOVI output in PDF format. Shows all analysis for Study Two.

Please note that a filter has been used to ensure only drinkers (those participants who have consumed alcohol in the last 12 months are included analysis). Specifically, the Output for Study Two presents:

1. Descriptives and Frequencies for all relevant variables, including histogram and box plots to allow visual inspection of the distribution of data.
2. Correlation matrix showing Spearman’s Rho for all correlations including hypothesised tests of validity
3. Reliability Analysis for 12-item total scale and each individual subscale (e.g., threat to fun, connection, and self)
4. Descriptive analysis and histograms for each individual CAN item, showing most items are not normally distributed.
5. Confirmatory Factor Analysis using weighted least square estimation method.
6. Path Model.
