# Supplementary material for: Development and Validation of the Cheers Attitudes towards Non-drinkers Scale (CANS)
Source: J Health Psychol. 2024 Jan 29;29(10):1101–14. doi: 10.1177/13591053231220519 (PMC11344955; doi:10.1177/13591053231220519)
Supplement: sj-omv-2-hpq-10.1177_13591053231220519 – Supplemental material for Development and Validation of the Cheers Attitudes towards Non-drinkers Scale (CANS) [file sj-omv-2-hpq-10.1177_13591053231220519.omv › index.html]

Results


# Results

# Exploratory Factor Analysis Phase 1

| Factor Loadings | | | | | | | | | |
| --- | --- | --- | --- | --- | --- | --- | --- | --- | --- |
|  | | Factor | | | | | |  | |
|  | | 1 | | 2 | | 3 | | Uniqueness | |
| CAN29 |  | 0.334 |  |  |  | 0.570 |  | 0.463 |  |
| CAN28 |  |  |  |  |  | 0.660 |  | 0.574 |  |
| CAN27 |  |  |  |  |  | 0.704 |  | 0.483 |  |
| CAN26 |  | 0.542 |  |  |  | 0.451 |  | 0.389 |  |
| CAN25 |  | 0.623 |  |  |  |  |  | 0.514 |  |
| CAN24 |  |  |  |  |  | 0.735 |  | 0.445 |  |
| CAN23 |  | 0.628 |  |  |  |  |  | 0.506 |  |
| CAN22 |  |  |  |  |  | 0.679 |  | 0.501 |  |
| CAN21 |  | 0.562 |  |  |  | 0.454 |  | 0.433 |  |
| CAN20 |  |  |  |  |  | 0.664 |  | 0.475 |  |
| CAN19 |  | 0.781 |  |  |  |  |  | 0.387 |  |
| CAN18 |  | 0.373 |  | 0.471 |  |  |  | 0.405 |  |
| CAN17 |  | 0.614 |  |  |  |  |  | 0.399 |  |
| CAN16 |  |  |  | 0.587 |  |  |  | 0.385 |  |
| CAN15 |  | 0.463 |  | 0.385 |  |  |  | 0.426 |  |
| CAN14 |  | 0.581 |  |  |  |  |  | 0.568 |  |
| CAN13 |  | 0.752 |  |  |  |  |  | 0.366 |  |
| CAN12 |  | 0.641 |  |  |  |  |  | 0.441 |  |
| CAN11 |  | 0.646 |  |  |  |  |  | 0.589 |  |
| CAN10 |  | 0.355 |  |  |  |  |  | 0.789 |  |
| CAN9 |  |  |  | 0.707 |  |  |  | 0.502 |  |
| CAN8 |  |  |  | 0.780 |  |  |  | 0.378 |  |
| CAN7 |  |  |  | 0.520 |  |  |  | 0.487 |  |
| CAN6 |  |  |  | 0.753 |  |  |  | 0.484 |  |
| CAN5 |  | 0.343 |  | 0.312 |  |  |  | 0.654 |  |
| CAN4 |  | 0.345 |  | 0.506 |  |  |  | 0.440 |  |
| CAN3 |  | 0.590 |  |  |  |  |  | 0.622 |  |
| CAN2 |  |  |  | 0.561 |  |  |  | 0.466 |  |
| CAN1 |  |  |  | 0.550 |  |  |  | 0.650 |  |
|  |  |  |  |  |  |  |  |  |  |
| --- | --- | --- | --- | --- | --- | --- | --- | --- | --- |
| Note. 'Principal axis factoring' extraction method was used in combination with a 'oblimin' rotation | | | | | | | | | |
|  | | | | | | | | | |
| [3] | | | | | | | | | |

## Factor Statistics

| Summary | | | | | | | |
| --- | --- | --- | --- | --- | --- | --- | --- |
| Factor | | SS Loadings | | % of Variance | | Cumulative % | |
| 1 |  | 6.55 |  | 22.6 |  | 22.6 |  |
| 2 |  | 4.77 |  | 16.4 |  | 39.0 |  |
| 3 |  | 3.45 |  | 11.9 |  | 50.9 |  |
|  |  |  |  |  |  |  |  |
| --- | --- | --- | --- | --- | --- | --- | --- |
|  | | | | | | | |
|  | | | | | | | |

| Inter-Factor Correlations | | | | | | | |
| --- | --- | --- | --- | --- | --- | --- | --- |
|  | | 1 | | 2 | | 3 | |
| 1 |  | — |  | 0.591 |  | 0.199 |  |
| 2 |  |  |  | — |  | 0.317 |  |
| 3 |  |  |  |  |  | — |  |
|  |  |  |  |  |  |  |  |
| --- | --- | --- | --- | --- | --- | --- | --- |
|  | | | | | | | |
|  | | | | | | | |

## Model Fit

| Model Fit Measures | | | | | | | | | | | | | | | |
| --- | --- | --- | --- | --- | --- | --- | --- | --- | --- | --- | --- | --- | --- | --- | --- |
|  | | RMSEA 90% CI | | | |  | | | | Model Test | | | | | |
| RMSEA | | Lower | | Upper | | TLI | | BIC | | χ² | | df | | p | |
| 0.0703 |  | 0.0655 |  | 0.0754 |  | 0.872 |  | -949 |  | 1000 |  | 322 |  | < .001 |  |
|  |  |  |  |  |  |  |  |  |  |  |  |  |  |  |  |
| --- | --- | --- | --- | --- | --- | --- | --- | --- | --- | --- | --- | --- | --- | --- | --- |
|  | | | | | | | | | | | | | | | |
|  | | | | | | | | | | | | | | | |

## Assumption Checks

| Bartlett's Test of Sphericity | | | | | |
| --- | --- | --- | --- | --- | --- |
| χ² | | df | | p | |
| 7125 |  | 406 |  | < .001 |  |
|  |  |  |  |  |  |
| --- | --- | --- | --- | --- | --- |
|  | | | | | |
|  | | | | | |

| KMO Measure of Sampling Adequacy | | | |
| --- | --- | --- | --- |
|  | | MSA | |
| Overall |  | 0.940 |  |
| CAN29 |  | 0.946 |  |
| CAN28 |  | 0.830 |  |
| CAN27 |  | 0.808 |  |
| CAN26 |  | 0.949 |  |
| CAN25 |  | 0.946 |  |
| CAN24 |  | 0.800 |  |
| CAN23 |  | 0.952 |  |
| CAN22 |  | 0.894 |  |
| CAN21 |  | 0.934 |  |
| CAN20 |  | 0.885 |  |
| CAN19 |  | 0.960 |  |
| CAN18 |  | 0.956 |  |
| CAN17 |  | 0.962 |  |
| CAN16 |  | 0.954 |  |
| CAN15 |  | 0.956 |  |
| CAN14 |  | 0.957 |  |
| CAN13 |  | 0.952 |  |
| CAN12 |  | 0.960 |  |
| CAN11 |  | 0.941 |  |
| CAN10 |  | 0.923 |  |
| CAN9 |  | 0.942 |  |
| CAN8 |  | 0.958 |  |
| CAN7 |  | 0.955 |  |
| CAN6 |  | 0.919 |  |
| CAN5 |  | 0.954 |  |
| CAN4 |  | 0.944 |  |
| CAN3 |  | 0.920 |  |
| CAN2 |  | 0.960 |  |
| CAN1 |  | 0.943 |  |
|  |  |  |  |
| --- | --- | --- | --- |
|  | | | |
|  | | | |

## Eigenvalues

| Initial Eigenvalues | | | |
| --- | --- | --- | --- |
| Factor | | Eigenvalue | |
| 1 |  | 10.67933 |  |
| 2 |  | 2.45138 |  |
| 3 |  | 1.09252 |  |
| 4 |  | 0.49968 |  |
| 5 |  | 0.23940 |  |
| 6 |  | 0.21252 |  |
| 7 |  | 0.17041 |  |
| 8 |  | 0.11018 |  |
| 9 |  | 0.09669 |  |
| 10 |  | -0.00140 |  |
| 11 |  | -0.03693 |  |
| 12 |  | -0.07776 |  |
| 13 |  | -0.09772 |  |
| 14 |  | -0.10408 |  |
| 15 |  | -0.11670 |  |
| 16 |  | -0.15498 |  |
| 17 |  | -0.17520 |  |
| 18 |  | -0.19987 |  |
| 19 |  | -0.21620 |  |
| 20 |  | -0.22655 |  |
| 21 |  | -0.23652 |  |
| 22 |  | -0.25894 |  |
| 23 |  | -0.27381 |  |
| 24 |  | -0.32900 |  |
| 25 |  | -0.35436 |  |
| 26 |  | -0.36852 |  |
| 27 |  | -0.43229 |  |
| 28 |  | -0.54945 |  |
| 29 |  | -0.66238 |  |
|  |  |  |  |
| --- | --- | --- | --- |
|  | | | |
|  | | | |

### Scree Plot

# Exploratory Factor Analysis Phase 2

| Factor Loadings | | | | | | | | | |
| --- | --- | --- | --- | --- | --- | --- | --- | --- | --- |
|  | | Factor | | | | | |  | |
|  | | 1 | | 2 | | 3 | | Uniqueness | |
| CAN28 |  |  |  |  |  | 0.674 |  | 0.565 |  |
| CAN27 |  |  |  |  |  | 0.758 |  | 0.424 |  |
| CAN24 |  |  |  |  |  | 0.782 |  | 0.392 |  |
| CAN23 |  | 0.580 |  |  |  |  |  | 0.558 |  |
| CAN22 |  |  |  |  |  | 0.606 |  | 0.591 |  |
| CAN20 |  |  |  |  |  | 0.672 |  | 0.463 |  |
| CAN19 |  | 0.828 |  |  |  |  |  | 0.366 |  |
| CAN17 |  | 0.698 |  |  |  |  |  | 0.382 |  |
| CAN14 |  | 0.603 |  |  |  |  |  | 0.586 |  |
| CAN13 |  | 0.848 |  |  |  |  |  | 0.311 |  |
| CAN12 |  | 0.692 |  |  |  |  |  | 0.430 |  |
| CAN9 |  |  |  | 0.737 |  |  |  | 0.458 |  |
| CAN8 |  |  |  | 0.808 |  |  |  | 0.351 |  |
| CAN7 |  | 0.317 |  | 0.473 |  |  |  | 0.496 |  |
| CAN6 |  |  |  | 0.758 |  |  |  | 0.470 |  |
| CAN3 |  | 0.594 |  |  |  |  |  | 0.635 |  |
| CAN2 |  | 0.301 |  | 0.504 |  |  |  | 0.482 |  |
| CAN1 |  |  |  | 0.511 |  |  |  | 0.657 |  |
| CAN25 |  | 0.632 |  |  |  |  |  | 0.538 |  |
| CAN11 |  | 0.660 |  |  |  |  |  | 0.583 |  |
| CAN16 |  |  |  | 0.531 |  |  |  | 0.416 |  |
|  |  |  |  |  |  |  |  |  |  |
| --- | --- | --- | --- | --- | --- | --- | --- | --- | --- |
| Note. 'Principal axis factoring' extraction method was used in combination with a 'oblimin' rotation | | | | | | | | | |
|  | | | | | | | | | |
| [3] | | | | | | | | | |

## Factor Statistics

| Summary | | | | | | | |
| --- | --- | --- | --- | --- | --- | --- | --- |
| Factor | | SS Loadings | | % of Variance | | Cumulative % | |
| 1 |  | 5.00 |  | 23.8 |  | 23.8 |  |
| 2 |  | 3.23 |  | 15.4 |  | 39.2 |  |
| 3 |  | 2.62 |  | 12.5 |  | 51.6 |  |
|  |  |  |  |  |  |  |  |
| --- | --- | --- | --- | --- | --- | --- | --- |
|  | | | | | | | |
|  | | | | | | | |

| Inter-Factor Correlations | | | | | | | |
| --- | --- | --- | --- | --- | --- | --- | --- |
|  | | 1 | | 2 | | 3 | |
| 1 |  | — |  | 0.592 |  | 0.119 |  |
| 2 |  |  |  | — |  | 0.343 |  |
| 3 |  |  |  |  |  | — |  |
|  |  |  |  |  |  |  |  |
| --- | --- | --- | --- | --- | --- | --- | --- |
|  | | | | | | | |
|  | | | | | | | |

## Model Fit

| Model Fit Measures | | | | | | | | | | | | | | | |
| --- | --- | --- | --- | --- | --- | --- | --- | --- | --- | --- | --- | --- | --- | --- | --- |
|  | | RMSEA 90% CI | | | |  | | | | Model Test | | | | | |
| RMSEA | | Lower | | Upper | | TLI | | BIC | | χ² | | df | | p | |
| 0.0616 |  | 0.0543 |  | 0.0692 |  | 0.920 |  | -515 |  | 393 |  | 150 |  | < .001 |  |
|  |  |  |  |  |  |  |  |  |  |  |  |  |  |  |  |
| --- | --- | --- | --- | --- | --- | --- | --- | --- | --- | --- | --- | --- | --- | --- | --- |
|  | | | | | | | | | | | | | | | |
|  | | | | | | | | | | | | | | | |

## Assumption Checks

| Bartlett's Test of Sphericity | | | | | |
| --- | --- | --- | --- | --- | --- |
| χ² | | df | | p | |
| 4470 |  | 210 |  | < .001 |  |
|  |  |  |  |  |  |
| --- | --- | --- | --- | --- | --- |
|  | | | | | |
|  | | | | | |

| KMO Measure of Sampling Adequacy | | | |
| --- | --- | --- | --- |
|  | | MSA | |
| Overall |  | 0.922 |  |
| CAN28 |  | 0.829 |  |
| CAN27 |  | 0.789 |  |
| CAN24 |  | 0.770 |  |
| CAN23 |  | 0.960 |  |
| CAN22 |  | 0.872 |  |
| CAN20 |  | 0.853 |  |
| CAN19 |  | 0.938 |  |
| CAN17 |  | 0.942 |  |
| CAN14 |  | 0.949 |  |
| CAN13 |  | 0.936 |  |
| CAN12 |  | 0.940 |  |
| CAN9 |  | 0.925 |  |
| CAN8 |  | 0.931 |  |
| CAN7 |  | 0.954 |  |
| CAN6 |  | 0.903 |  |
| CAN3 |  | 0.909 |  |
| CAN2 |  | 0.952 |  |
| CAN1 |  | 0.927 |  |
| CAN25 |  | 0.941 |  |
| CAN11 |  | 0.935 |  |
| CAN16 |  | 0.955 |  |
|  |  |  |  |
| --- | --- | --- | --- |
|  | | | |
|  | | | |

## Eigenvalues

| Initial Eigenvalues | | | |
| --- | --- | --- | --- |
| Factor | | Eigenvalue | |
| 1 |  | 7.2415 |  |
| 2 |  | 2.0994 |  |
| 3 |  | 0.8446 |  |
| 4 |  | 0.2290 |  |
| 5 |  | 0.1252 |  |
| 6 |  | 0.1081 |  |
| 7 |  | 0.0576 |  |
| 8 |  | 0.0143 |  |
| 9 |  | -0.0526 |  |
| 10 |  | -0.1101 |  |
| 11 |  | -0.1267 |  |
| 12 |  | -0.1440 |  |
| 13 |  | -0.1784 |  |
| 14 |  | -0.1952 |  |
| 15 |  | -0.2104 |  |
| 16 |  | -0.2803 |  |
| 17 |  | -0.2933 |  |
| 18 |  | -0.3016 |  |
| 19 |  | -0.3964 |  |
| 20 |  | -0.5274 |  |
| 21 |  | -0.6618 |  |
|  |  |  |  |
| --- | --- | --- | --- |
|  | | | |
|  | | | |

### Scree Plot

# Exploratory Factor Analysis Phase 3

| Factor Loadings | | | | | | | | | |
| --- | --- | --- | --- | --- | --- | --- | --- | --- | --- |
|  | | Factor | | | | | |  | |
|  | | 1 | | 2 | | 3 | | Uniqueness | |
| CAN13 |  | 0.849 |  |  |  |  |  | 0.311 |  |
| CAN19 |  | 0.827 |  |  |  |  |  | 0.364 |  |
| CAN17 |  | 0.704 |  |  |  |  |  | 0.377 |  |
| CAN12 |  | 0.700 |  |  |  |  |  | 0.439 |  |
| CAN11 |  | 0.656 |  |  |  |  |  | 0.576 |  |
| CAN25 |  | 0.641 |  |  |  |  |  | 0.546 |  |
| CAN14 |  | 0.599 |  |  |  |  |  | 0.584 |  |
| CAN3 |  | 0.598 |  |  |  |  |  | 0.633 |  |
| CAN23 |  | 0.588 |  |  |  |  |  | 0.559 |  |
| CAN24 |  |  |  | 0.779 |  |  |  | 0.394 |  |
| CAN27 |  |  |  | 0.753 |  |  |  | 0.426 |  |
| CAN28 |  |  |  | 0.683 |  |  |  | 0.559 |  |
| CAN20 |  |  |  | 0.675 |  |  |  | 0.463 |  |
| CAN22 |  |  |  | 0.596 |  |  |  | 0.592 |  |
| CAN8 |  |  |  |  |  | 0.781 |  | 0.357 |  |
| CAN6 |  |  |  |  |  | 0.761 |  | 0.452 |  |
| CAN9 |  |  |  |  |  | 0.738 |  | 0.442 |  |
| CAN16 |  | 0.315 |  |  |  | 0.527 |  | 0.406 |  |
| CAN1 |  |  |  |  |  | 0.456 |  | 0.684 |  |
|  |  |  |  |  |  |  |  |  |  |
| --- | --- | --- | --- | --- | --- | --- | --- | --- | --- |
| Note. 'Principal axis factoring' extraction method was used in combination with a 'oblimin' rotation | | | | | | | | | |
|  | | | | | | | | | |
| [3] | | | | | | | | | |

## Factor Statistics

| Summary | | | | | | | |
| --- | --- | --- | --- | --- | --- | --- | --- |
| Factor | | SS Loadings | | % of Variance | | Cumulative % | |
| 1 |  | 4.72 |  | 24.9 |  | 24.9 |  |
| 2 |  | 2.60 |  | 13.7 |  | 38.6 |  |
| 3 |  | 2.51 |  | 13.2 |  | 51.8 |  |
|  |  |  |  |  |  |  |  |
| --- | --- | --- | --- | --- | --- | --- | --- |
|  | | | | | | | |
|  | | | | | | | |

| Inter-Factor Correlations | | | | | | | |
| --- | --- | --- | --- | --- | --- | --- | --- |
|  | | 1 | | 2 | | 3 | |
| 1 |  | — |  | 0.123 |  | 0.561 |  |
| 2 |  |  |  | — |  | 0.356 |  |
| 3 |  |  |  |  |  | — |  |
|  |  |  |  |  |  |  |  |
| --- | --- | --- | --- | --- | --- | --- | --- |
|  | | | | | | | |
|  | | | | | | | |

## Model Fit

| Model Fit Measures | | | | | | | | | | | | | | | |
| --- | --- | --- | --- | --- | --- | --- | --- | --- | --- | --- | --- | --- | --- | --- | --- |
|  | | RMSEA 90% CI | | | |  | | | | Model Test | | | | | |
| RMSEA | | Lower | | Upper | | TLI | | BIC | | χ² | | df | | p | |
| 0.0643 |  | 0.0561 |  | 0.0728 |  | 0.918 |  | -385 |  | 323 |  | 117 |  | < .001 |  |
|  |  |  |  |  |  |  |  |  |  |  |  |  |  |  |  |
| --- | --- | --- | --- | --- | --- | --- | --- | --- | --- | --- | --- | --- | --- | --- | --- |
|  | | | | | | | | | | | | | | | |
|  | | | | | | | | | | | | | | | |

## Assumption Checks

| Bartlett's Test of Sphericity | | | | | |
| --- | --- | --- | --- | --- | --- |
| χ² | | df | | p | |
| 3886 |  | 171 |  | < .001 |  |
|  |  |  |  |  |  |
| --- | --- | --- | --- | --- | --- |
|  | | | | | |
|  | | | | | |

| KMO Measure of Sampling Adequacy | | | |
| --- | --- | --- | --- |
|  | | MSA | |
| Overall |  | 0.908 |  |
| CAN28 |  | 0.822 |  |
| CAN27 |  | 0.791 |  |
| CAN24 |  | 0.771 |  |
| CAN23 |  | 0.952 |  |
| CAN22 |  | 0.875 |  |
| CAN20 |  | 0.843 |  |
| CAN19 |  | 0.929 |  |
| CAN17 |  | 0.938 |  |
| CAN14 |  | 0.942 |  |
| CAN13 |  | 0.927 |  |
| CAN12 |  | 0.931 |  |
| CAN9 |  | 0.910 |  |
| CAN8 |  | 0.910 |  |
| CAN6 |  | 0.878 |  |
| CAN1 |  | 0.932 |  |
| CAN25 |  | 0.940 |  |
| CAN11 |  | 0.935 |  |
| CAN3 |  | 0.897 |  |
| CAN16 |  | 0.944 |  |
|  |  |  |  |
| --- | --- | --- | --- |
|  | | | |
|  | | | |

## Eigenvalues

| Initial Eigenvalues | | | |
| --- | --- | --- | --- |
| Factor | | Eigenvalue | |
| 1 |  | 6.28051 |  |
| 2 |  | 2.10399 |  |
| 3 |  | 0.75427 |  |
| 4 |  | 0.20675 |  |
| 5 |  | 0.09922 |  |
| 6 |  | 0.08268 |  |
| 7 |  | 0.00130 |  |
| 8 |  | -0.04661 |  |
| 9 |  | -0.11745 |  |
| 10 |  | -0.13659 |  |
| 11 |  | -0.14195 |  |
| 12 |  | -0.17862 |  |
| 13 |  | -0.19513 |  |
| 14 |  | -0.27295 |  |
| 15 |  | -0.28587 |  |
| 16 |  | -0.31271 |  |
| 17 |  | -0.38102 |  |
| 18 |  | -0.52334 |  |
| 19 |  | -0.65606 |  |
|  |  |  |  |
| --- | --- | --- | --- |
|  | | | |
|  | | | |

### Scree Plot

# Exploratory Factor Analysis Final Solution

| Factor Loadings | | | | | | | | | |
| --- | --- | --- | --- | --- | --- | --- | --- | --- | --- |
|  | | Factor | | | | | |  | |
|  | | 1 | | 2 | | 3 | | Uniqueness | |
| CAN13 |  | 0.841 |  |  |  |  |  | 0.316 |  |
| CAN12 |  | 0.799 |  |  |  |  |  | 0.338 |  |
| CAN25 |  | 0.612 |  |  |  |  |  | 0.571 |  |
| CAN14 |  | 0.599 |  |  |  |  |  | 0.592 |  |
| CAN24 |  |  |  | 0.829 |  |  |  | 0.322 |  |
| CAN27 |  |  |  | 0.792 |  |  |  | 0.360 |  |
| CAN28 |  |  |  | 0.590 |  |  |  | 0.648 |  |
| CAN22 |  |  |  | 0.531 |  |  |  | 0.636 |  |
| CAN8 |  |  |  |  |  | 0.771 |  | 0.367 |  |
| CAN9 |  |  |  |  |  | 0.755 |  | 0.423 |  |
| CAN6 |  |  |  |  |  | 0.747 |  | 0.443 |  |
| CAN1 |  |  |  |  |  | 0.463 |  | 0.700 |  |
|  |  |  |  |  |  |  |  |  |  |
| --- | --- | --- | --- | --- | --- | --- | --- | --- | --- |
| Note. 'Principal axis factoring' extraction method was used in combination with a 'oblimin' rotation | | | | | | | | | |
|  | | | | | | | | | |
| [3] | | | | | | | | | |

## Assumption Checks

| KMO Measure of Sampling Adequacy | | | |
| --- | --- | --- | --- |
|  | | MSA | |
| Overall |  | 0.826 |  |
| CAN28 |  | 0.836 |  |
| CAN27 |  | 0.722 |  |
| CAN24 |  | 0.708 |  |
| CAN22 |  | 0.854 |  |
| CAN14 |  | 0.857 |  |
| CAN13 |  | 0.812 |  |
| CAN12 |  | 0.844 |  |
| CAN9 |  | 0.846 |  |
| CAN8 |  | 0.846 |  |
| CAN6 |  | 0.831 |  |
| CAN1 |  | 0.885 |  |
| CAN25 |  | 0.893 |  |
|  |  |  |  |
| --- | --- | --- | --- |
|  | | | |
|  | | | |

## Eigenvalues

| Initial Eigenvalues | | | |
| --- | --- | --- | --- |
| Factor | | Eigenvalue | |
| 1 |  | 3.5790 |  |
| 2 |  | 1.3236 |  |
| 3 |  | 0.6248 |  |
| 4 |  | 0.0587 |  |
| 5 |  | -0.0817 |  |
| 6 |  | -0.1261 |  |
| 7 |  | -0.1716 |  |
| 8 |  | -0.1893 |  |
| 9 |  | -0.2318 |  |
| 10 |  | -0.2721 |  |
| 11 |  | -0.3607 |  |
| 12 |  | -0.5740 |  |
|  |  |  |  |
| --- | --- | --- | --- |
|  | | | |
|  | | | |

### Scree Plot

# Descriptives

| Descriptives | | | | | | | | | | | |
| --- | --- | --- | --- | --- | --- | --- | --- | --- | --- | --- | --- |
|  | | Age | | Gender | | Education | | Student | | Location | |
| N |  | 426 |  | 426 |  | 426 |  | 426 |  | 426 |  |
| Missing |  | 0 |  | 0 |  | 0 |  | 0 |  | 0 |  |
| Mean |  | 37.3 |  | 1.52 |  | 3.70 |  | 2.56 |  | 2.70 |  |
| Median |  | 39.0 |  | 1.00 |  | 4.00 |  | 3.00 |  | 2.00 |  |
| Standard deviation |  | 9.37 |  | 0.626 |  | 1.19 |  | 0.753 |  | 1.68 |  |
| Minimum |  | 18.0 |  | 1 |  | 1 |  | 1 |  | 1 |  |
| Maximum |  | 50.0 |  | 4 |  | 6 |  | 3 |  | 8 |  |
|  |  |  |  |  |  |  |  |  |  |  |  |
| --- | --- | --- | --- | --- | --- | --- | --- | --- | --- | --- | --- |
|  | | | | | | | | | | | |
|  | | | | | | | | | | | |

## Frequencies

| Frequencies of Gender | | | | | | | |
| --- | --- | --- | --- | --- | --- | --- | --- |
| Levels | | Counts | | % of Total | | Cumulative % | |
| Male (including transgender men) |  | 225 |  | 52.8 % |  | 52.8 % |  |
| Female (including transgender women) |  | 189 |  | 44.4 % |  | 97.2 % |  |
| Prefer not to say |  | 3 |  | 0.7 % |  | 97.9 % |  |
| Prefer to self-describe as \_\_\_\_\_\_\_\_\_ (e.g. non-binary, gende |  | 9 |  | 2.1 % |  | 100.0 % |  |
|  |  |  |  |  |  |  |  |
| --- | --- | --- | --- | --- | --- | --- | --- |
|  | | | | | | | |
|  | | | | | | | |

| Frequencies of Education | | | | | | | |
| --- | --- | --- | --- | --- | --- | --- | --- |
| Levels | | Counts | | % of Total | | Cumulative % | |
| Year 11 or below |  | 12 |  | 2.8 % |  | 2.8 % |  |
| Year 12 |  | 65 |  | 15.3 % |  | 18.1 % |  |
| Graduate Certificate, Diploma (including TAFE) |  | 87 |  | 20.4 % |  | 38.5 % |  |
| Bachelor's degree |  | 163 |  | 38.3 % |  | 76.8 % |  |
| Master's degree |  | 72 |  | 16.9 % |  | 93.7 % |  |
| PhD/Doctorate |  | 27 |  | 6.3 % |  | 100.0 % |  |
|  |  |  |  |  |  |  |  |
| --- | --- | --- | --- | --- | --- | --- | --- |
|  | | | | | | | |
|  | | | | | | | |

| Frequencies of Student | | | | | | | |
| --- | --- | --- | --- | --- | --- | --- | --- |
| Levels | | Counts | | % of Total | | Cumulative % | |
| Yes, undergraduate |  | 68 |  | 16.0 % |  | 16.0 % |  |
| Yes, postgraduate |  | 50 |  | 11.7 % |  | 27.7 % |  |
| No |  | 308 |  | 72.3 % |  | 100.0 % |  |
|  |  |  |  |  |  |  |  |
| --- | --- | --- | --- | --- | --- | --- | --- |
|  | | | | | | | |
|  | | | | | | | |

| Frequencies of Location | | | | | | | |
| --- | --- | --- | --- | --- | --- | --- | --- |
| Levels | | Counts | | % of Total | | Cumulative % | |
| NSW |  | 89 |  | 20.9 % |  | 20.9 % |  |
| VIC |  | 182 |  | 42.7 % |  | 63.6 % |  |
| QLD |  | 53 |  | 12.4 % |  | 76.1 % |  |
| SA |  | 35 |  | 8.2 % |  | 84.3 % |  |
| WA |  | 33 |  | 7.7 % |  | 92.0 % |  |
| TAS |  | 17 |  | 4.0 % |  | 96.0 % |  |
| NT |  | 5 |  | 1.2 % |  | 97.2 % |  |
| ACT |  | 12 |  | 2.8 % |  | 100.0 % |  |
|  |  |  |  |  |  |  |  |
| --- | --- | --- | --- | --- | --- | --- | --- |
|  | | | | | | | |
|  | | | | | | | |

# Descriptives

| Descriptives | | | | | | | | | | | | | | | | | | | | | | | | | |
| --- | --- | --- | --- | --- | --- | --- | --- | --- | --- | --- | --- | --- | --- | --- | --- | --- | --- | --- | --- | --- | --- | --- | --- | --- | --- |
|  | | CAN13 | | CAN14 | | CAN12 | | CAN25 | | CAN22 | | CAN24 | | CAN27 | | CAN28 | | CAN6 | | CAN8 | | CAN9 | | CAN1 | |
| N |  | 426 |  | 426 |  | 426 |  | 426 |  | 426 |  | 426 |  | 426 |  | 426 |  | 426 |  | 426 |  | 426 |  | 426 |  |
| Missing |  | 0 |  | 0 |  | 0 |  | 0 |  | 0 |  | 0 |  | 0 |  | 0 |  | 0 |  | 0 |  | 0 |  | 0 |  |
| Mean |  | 1.62 |  | 1.83 |  | 1.75 |  | 1.87 |  | 3.01 |  | 3.74 |  | 3.54 |  | 2.60 |  | 3.57 |  | 2.91 |  | 3.10 |  | 2.33 |  |
| Median |  | 1.00 |  | 2.00 |  | 2.00 |  | 2.00 |  | 3.00 |  | 4.00 |  | 4.00 |  | 2.00 |  | 4.00 |  | 3.00 |  | 3.00 |  | 2.00 |  |
| Standard deviation |  | 0.792 |  | 1.01 |  | 0.921 |  | 0.965 |  | 1.26 |  | 1.05 |  | 1.14 |  | 1.14 |  | 0.975 |  | 1.14 |  | 1.14 |  | 0.993 |  |
| Minimum |  | 1 |  | 1 |  | 1 |  | 1 |  | 1 |  | 1 |  | 1 |  | 1 |  | 1 |  | 1 |  | 1 |  | 1 |  |
| Maximum |  | 5 |  | 5 |  | 5 |  | 5 |  | 5 |  | 5 |  | 5 |  | 5 |  | 5 |  | 5 |  | 5 |  | 5 |  |
| Skewness |  | 1.39 |  | 1.17 |  | 1.27 |  | 1.03 |  | -0.299 |  | -0.802 |  | -0.540 |  | 0.395 |  | -0.924 |  | -0.272 |  | -0.453 |  | 0.424 |  |
| Std. error skewness |  | 0.118 |  | 0.118 |  | 0.118 |  | 0.118 |  | 0.118 |  | 0.118 |  | 0.118 |  | 0.118 |  | 0.118 |  | 0.118 |  | 0.118 |  | 0.118 |  |
| Kurtosis |  | 2.03 |  | 0.570 |  | 1.08 |  | 0.417 |  | -1.20 |  | 0.210 |  | -0.475 |  | -0.651 |  | 0.475 |  | -1.07 |  | -0.859 |  | -0.553 |  |
| Std. error kurtosis |  | 0.236 |  | 0.236 |  | 0.236 |  | 0.236 |  | 0.236 |  | 0.236 |  | 0.236 |  | 0.236 |  | 0.236 |  | 0.236 |  | 0.236 |  | 0.236 |  |
| Shapiro-Wilk W |  | 0.734 |  | 0.769 |  | 0.758 |  | 0.799 |  | 0.864 |  | 0.861 |  | 0.889 |  | 0.901 |  | 0.825 |  | 0.879 |  | 0.870 |  | 0.881 |  |
| Shapiro-Wilk p |  | < .001 |  | < .001 |  | < .001 |  | < .001 |  | < .001 |  | < .001 |  | < .001 |  | < .001 |  | < .001 |  | < .001 |  | < .001 |  | < .001 |  |
|  |  |  |  |  |  |  |  |  |  |  |  |  |  |  |  |  |  |  |  |  |  |  |  |  |  |
| --- | --- | --- | --- | --- | --- | --- | --- | --- | --- | --- | --- | --- | --- | --- | --- | --- | --- | --- | --- | --- | --- | --- | --- | --- | --- |
|  | | | | | | | | | | | | | | | | | | | | | | | | | |
|  | | | | | | | | | | | | | | | | | | | | | | | | | |

# Correlation Matrix

| Correlation Matrix | | | | | | | | | | | | | | | | | |
| --- | --- | --- | --- | --- | --- | --- | --- | --- | --- | --- | --- | --- | --- | --- | --- | --- | --- |
|  | |  | | CAN\_12item\_TOTAL | | Fun Threat Factor | | Self Threat Factor | | Connection Threat Factor | | RAND\_Total | | AUDIT\_Total | | Total\_Volume | |
| CAN\_12item\_TOTAL |  | Spearman's rho |  | — |  |  |  |  |  |  |  |  |  |  |  |  |  |
|  |  | N |  | — |  |  |  |  |  |  |  |  |  |  |  |  |  |
| Fun Threat Factor |  | Spearman's rho |  | 0.727 |  | — |  |  |  |  |  |  |  |  |  |  |  |
|  |  | N |  | 426 |  | — |  |  |  |  |  |  |  |  |  |  |  |
| Self Threat Factor |  | Spearman's rho |  | 0.698 |  | 0.228 |  | — |  |  |  |  |  |  |  |  |  |
|  |  | N |  | 426 |  | 426 |  | — |  |  |  |  |  |  |  |  |  |
| Connection Threat Factor |  | Spearman's rho |  | 0.782 |  | 0.506 |  | 0.281 |  | — |  |  |  |  |  |  |  |
|  |  | N |  | 426 |  | 426 |  | 426 |  | — |  |  |  |  |  |  |  |
| RAND\_Total |  | Spearman's rho |  | 0.634 |  | 0.661 |  | 0.193 |  | 0.624 |  | — |  |  |  |  |  |
|  |  | N |  | 426 |  | 426 |  | 426 |  | 426 |  | — |  |  |  |  |  |
| AUDIT\_Total |  | Spearman's rho |  | 0.637 |  | 0.332 |  | 0.518 |  | 0.543 |  | 0.458 |  | — |  |  |  |
|  |  | N |  | 404 |  | 404 |  | 404 |  | 404 |  | 404 |  | — |  |  |  |
| Total\_Volume |  | Spearman's rho |  | 0.564 |  | 0.348 |  | 0.398 |  | 0.512 |  | 0.468 |  | 0.880 |  | — |  |
|  |  | N |  | 418 |  | 418 |  | 418 |  | 418 |  | 418 |  | 403 |  | — |  |
|  |  |  |  |  |  |  |  |  |  |  |  |  |  |  |  |  |  |
| --- | --- | --- | --- | --- | --- | --- | --- | --- | --- | --- | --- | --- | --- | --- | --- | --- | --- |
|  | | | | | | | | | | | | | | | | | |
|  | | | | | | | | | | | | | | | | | |

# Reliability Analysis CAN 12-items

| Scale Reliability Statistics | | | | | | | |
| --- | --- | --- | --- | --- | --- | --- | --- |
|  | | Mean | | SD | | Cronbach's α | |
| scale |  | 2.66 |  | 0.614 |  | 0.824 |  |
|  |  |  |  |  |  |  |  |
| --- | --- | --- | --- | --- | --- | --- | --- |
|  | | | | | | | |
| [3] | | | | | | | |

| Item Reliability Statistics | | | | | | | | | |
| --- | --- | --- | --- | --- | --- | --- | --- | --- | --- |
|  | | | | | | | | If item dropped | |
|  | | Mean | | SD | | Item-rest correlation | | Cronbach's α | |
| CAN13 |  | 1.62 |  | 0.792 |  | 0.486 |  | 0.812 |  |
| CAN14 |  | 1.83 |  | 1.008 |  | 0.470 |  | 0.812 |  |
| CAN12 |  | 1.75 |  | 0.921 |  | 0.547 |  | 0.806 |  |
| CAN25 |  | 1.87 |  | 0.965 |  | 0.495 |  | 0.810 |  |
| CAN22 |  | 3.01 |  | 1.260 |  | 0.474 |  | 0.812 |  |
| CAN24 |  | 3.74 |  | 1.054 |  | 0.410 |  | 0.817 |  |
| CAN27 |  | 3.54 |  | 1.144 |  | 0.440 |  | 0.814 |  |
| CAN28 |  | 2.60 |  | 1.144 |  | 0.370 |  | 0.821 |  |
| CAN1 |  | 2.33 |  | 0.993 |  | 0.424 |  | 0.815 |  |
| CAN6 |  | 3.57 |  | 0.975 |  | 0.562 |  | 0.805 |  |
| CAN8 |  | 2.91 |  | 1.142 |  | 0.552 |  | 0.804 |  |
| CAN9 |  | 3.10 |  | 1.139 |  | 0.561 |  | 0.804 |  |
|  |  |  |  |  |  |  |  |  |  |
| --- | --- | --- | --- | --- | --- | --- | --- | --- | --- |
|  | | | | | | | | | |
|  | | | | | | | | | |

# Reliability Analysis Fun

| Scale Reliability Statistics | | | | | | | |
| --- | --- | --- | --- | --- | --- | --- | --- |
|  | | Mean | | SD | | Cronbach's α | |
| scale |  | 1.77 |  | 0.738 |  | 0.810 |  |
|  |  |  |  |  |  |  |  |
| --- | --- | --- | --- | --- | --- | --- | --- |
|  | | | | | | | |
| [3] | | | | | | | |

| Item Reliability Statistics | | | | | | | | | |
| --- | --- | --- | --- | --- | --- | --- | --- | --- | --- |
|  | | | | | | | | If item dropped | |
|  | | Mean | | SD | | Item-rest correlation | | Cronbach's α | |
| CAN13 |  | 1.62 |  | 0.792 |  | 0.712 |  | 0.732 |  |
| CAN14 |  | 1.83 |  | 1.008 |  | 0.562 |  | 0.797 |  |
| CAN12 |  | 1.75 |  | 0.921 |  | 0.699 |  | 0.727 |  |
| CAN25 |  | 1.87 |  | 0.965 |  | 0.568 |  | 0.791 |  |
|  |  |  |  |  |  |  |  |  |  |
| --- | --- | --- | --- | --- | --- | --- | --- | --- | --- |
|  | | | | | | | | | |
|  | | | | | | | | | |

# Reliability Analysis Self

| Scale Reliability Statistics | | | | | | | |
| --- | --- | --- | --- | --- | --- | --- | --- |
|  | | Mean | | SD | | Cronbach's α | |
| scale |  | 3.22 |  | 0.895 |  | 0.780 |  |
|  |  |  |  |  |  |  |  |
| --- | --- | --- | --- | --- | --- | --- | --- |
|  | | | | | | | |
| [3] | | | | | | | |

| Item Reliability Statistics | | | | | | | | | |
| --- | --- | --- | --- | --- | --- | --- | --- | --- | --- |
|  | | | | | | | | If item dropped | |
|  | | Mean | | SD | | Item-rest correlation | | Cronbach's α | |
| CAN22 |  | 3.01 |  | 1.26 |  | 0.499 |  | 0.776 |  |
| CAN24 |  | 3.74 |  | 1.05 |  | 0.663 |  | 0.691 |  |
| CAN27 |  | 3.54 |  | 1.14 |  | 0.659 |  | 0.688 |  |
| CAN28 |  | 2.60 |  | 1.14 |  | 0.539 |  | 0.750 |  |
|  |  |  |  |  |  |  |  |  |  |
| --- | --- | --- | --- | --- | --- | --- | --- | --- | --- |
|  | | | | | | | | | |
|  | | | | | | | | | |

# Reliability Analysis Connection

| Scale Reliability Statistics | | | | | | | |
| --- | --- | --- | --- | --- | --- | --- | --- |
|  | | mean | | sd | | Cronbach's α | |
| scale |  | 2.98 |  | 0.837 |  | 0.793 |  |
|  |  |  |  |  |  |  |  |
| --- | --- | --- | --- | --- | --- | --- | --- |
|  | | | | | | | |
| [3] | | | | | | | |

| Item Reliability Statistics | | | | | | | | | |
| --- | --- | --- | --- | --- | --- | --- | --- | --- | --- |
|  | | | | | | | | if item dropped | |
|  | | mean | | sd | | item-rest correlation | | Cronbach's α | |
| CAN1 |  | 2.33 |  | 0.993 |  | 0.481 |  | 0.798 |  |
| CAN6 |  | 3.57 |  | 0.975 |  | 0.619 |  | 0.736 |  |
| CAN8 |  | 2.91 |  | 1.142 |  | 0.671 |  | 0.706 |  |
| CAN9 |  | 3.10 |  | 1.139 |  | 0.653 |  | 0.716 |  |
|  |  |  |  |  |  |  |  |  |  |
| --- | --- | --- | --- | --- | --- | --- | --- | --- | --- |
|  | | | | | | | | | |
|  | | | | | | | | | |

# References

[1]
The jamovi project (2022). *jamovi*. (Version 2.3) [Computer Software]. Retrieved from https://www.jamovi.org.

[2]
R Core Team (2021). *R: A Language and environment for statistical computing*. (Version 4.1) [Computer software]. Retrieved from https://cran.r-project.org. (R packages retrieved from MRAN snapshot 2022-01-01).

[3]
Revelle, W. (2019). *psych: Procedures for Psychological, Psychometric, and Personality Research*. [R package]. Retrieved from https://cran.r-project.org/package=psych.
