# Supplementary figures and images for: Development and Validation of the Cheers Attitudes towards Non-drinkers Scale (CANS)
Source: J Health Psychol. 2024 Jan 29;29(10):1101–14. doi: 10.1177/13591053231220519 (PMC11344955; doi:10.1177/13591053231220519)

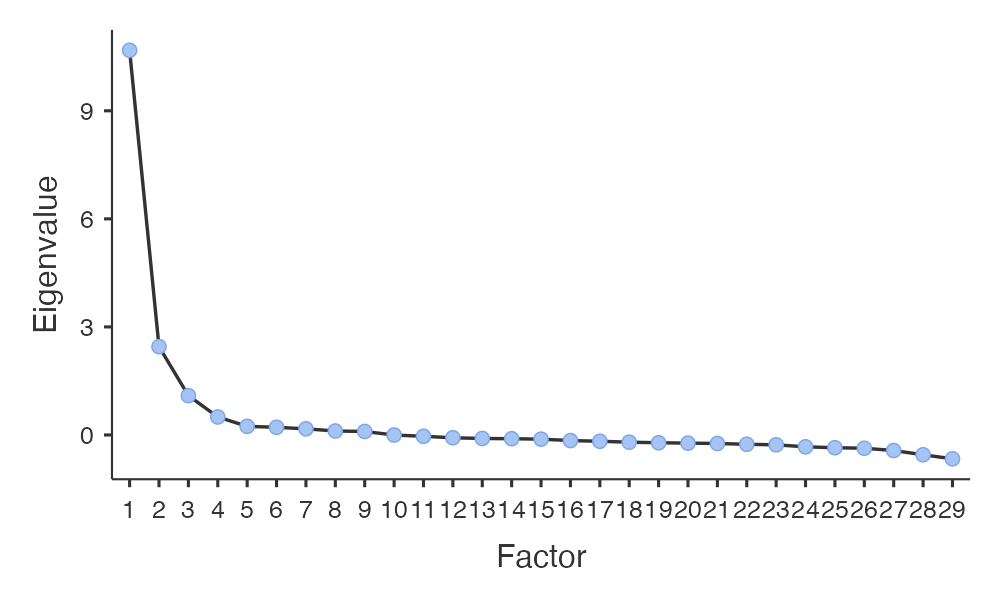

Supplement: sj-omv-2-hpq-10.1177_13591053231220519 – Supplemental material for Development and Validation of the Cheers Attitudes towards Non-drinkers Scale (CANS) [file sj-omv-2-hpq-10.1177_13591053231220519.omv › 02 efa/resources/db527b12ec17e964.png]

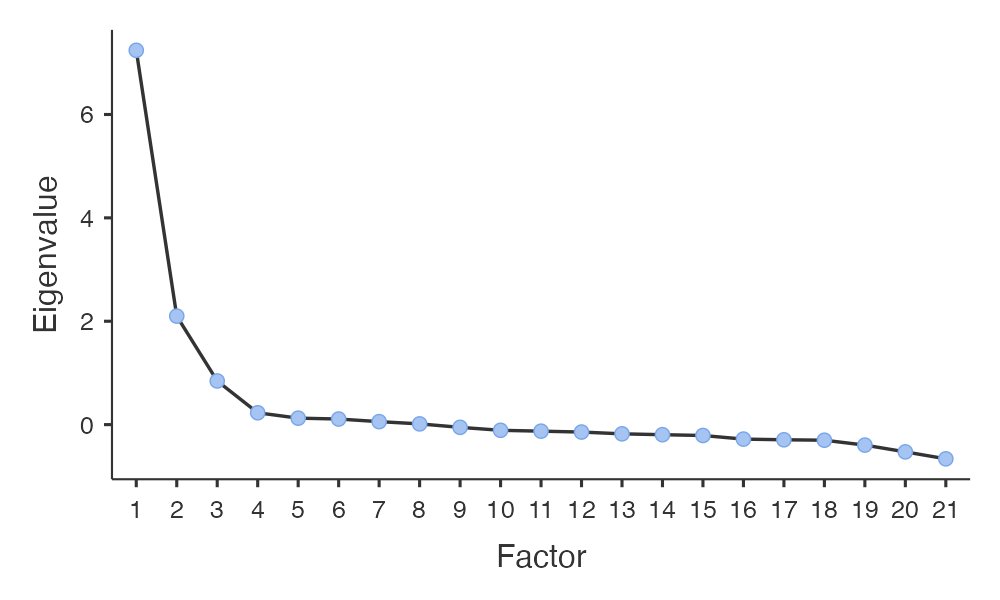

Supplement: sj-omv-2-hpq-10.1177_13591053231220519 – Supplemental material for Development and Validation of the Cheers Attitudes towards Non-drinkers Scale (CANS) [file sj-omv-2-hpq-10.1177_13591053231220519.omv › 30 efa/resources/08a7c0ffad7b6269.png]

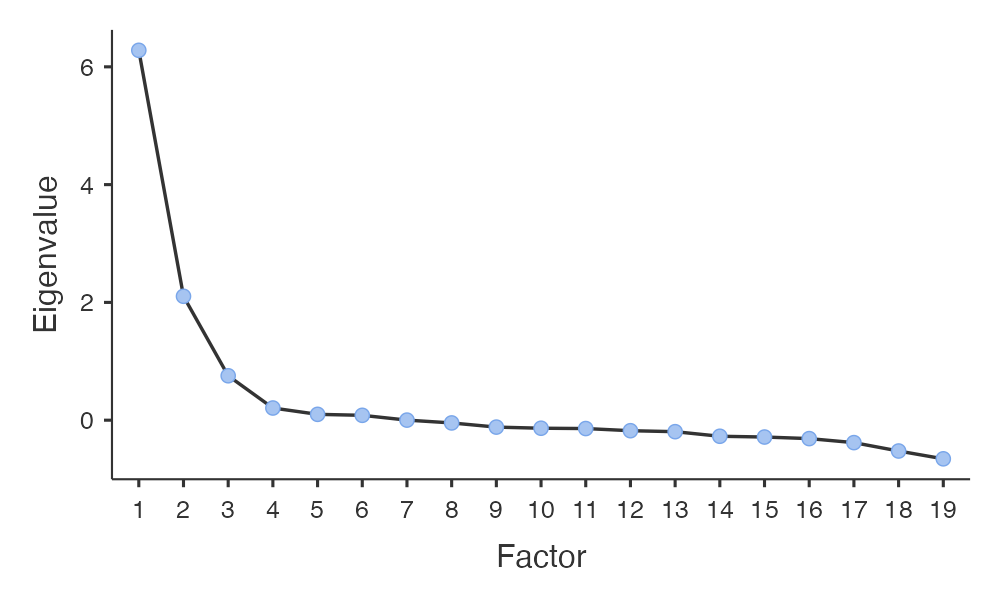

Supplement: sj-omv-2-hpq-10.1177_13591053231220519 – Supplemental material for Development and Validation of the Cheers Attitudes towards Non-drinkers Scale (CANS) [file sj-omv-2-hpq-10.1177_13591053231220519.omv › 38 efa/resources/bf79b9dc613e0544.png]

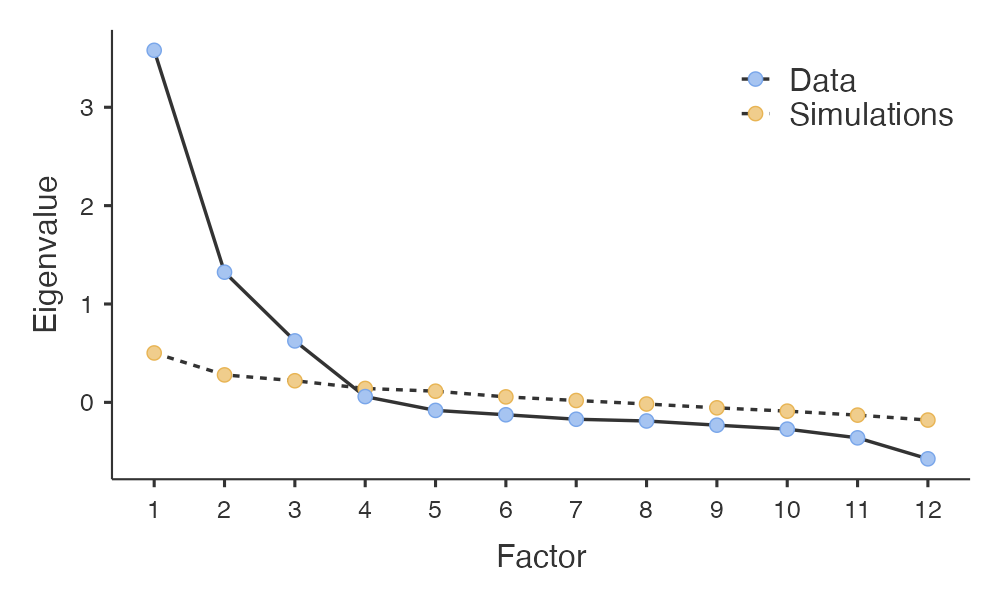

Supplement: sj-omv-2-hpq-10.1177_13591053231220519 – Supplemental material for Development and Validation of the Cheers Attitudes towards Non-drinkers Scale (CANS) [file sj-omv-2-hpq-10.1177_13591053231220519.omv › 34 efa/resources/591ccd54f959a981.png]

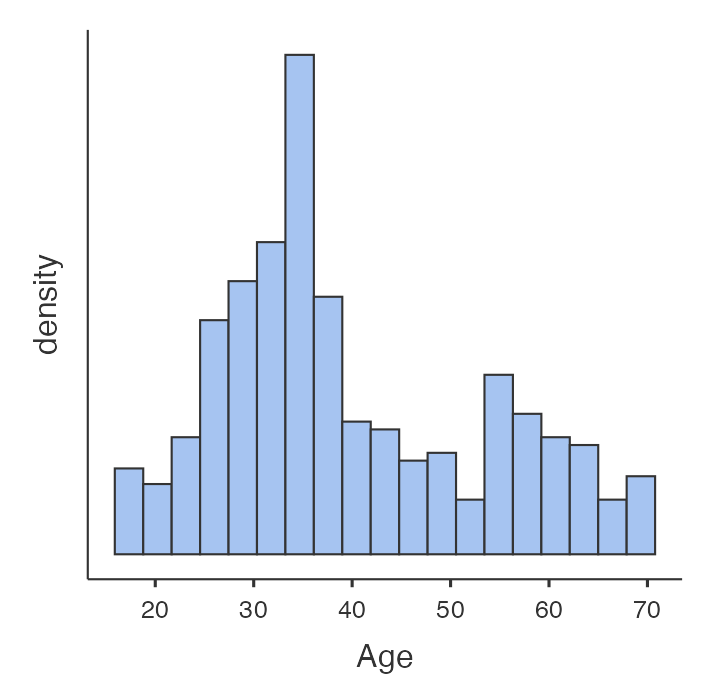

Supplement: sj-omv-5-hpq-10.1177_13591053231220519 – Supplemental material for Development and Validation of the Cheers Attitudes towards Non-drinkers Scale (CANS) [file sj-omv-5-hpq-10.1177_13591053231220519.omv › 02 descriptives/resources/bd3ff1e5eb25066d.png]

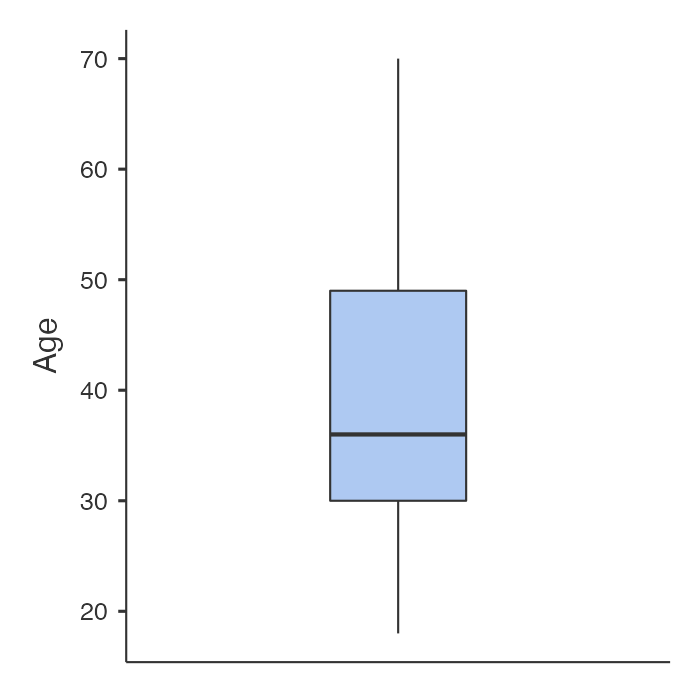

Supplement: sj-omv-5-hpq-10.1177_13591053231220519 – Supplemental material for Development and Validation of the Cheers Attitudes towards Non-drinkers Scale (CANS) [file sj-omv-5-hpq-10.1177_13591053231220519.omv › 02 descriptives/resources/7b17493605dae2bf.png]

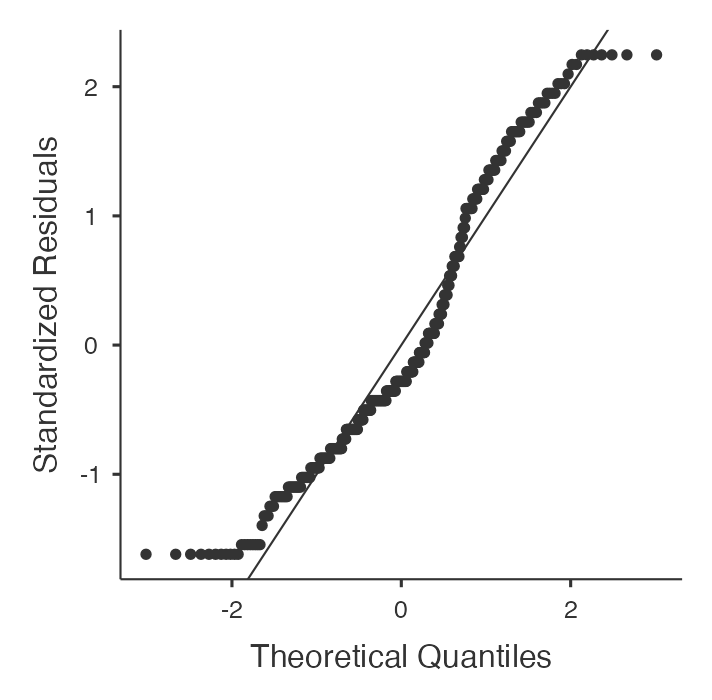

Supplement: sj-omv-5-hpq-10.1177_13591053231220519 – Supplemental material for Development and Validation of the Cheers Attitudes towards Non-drinkers Scale (CANS) [file sj-omv-5-hpq-10.1177_13591053231220519.omv › 02 descriptives/resources/bac2a1a800d448ee.png]

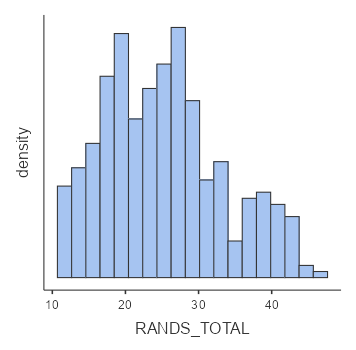

Supplement: sj-omv-5-hpq-10.1177_13591053231220519 – Supplemental material for Development and Validation of the Cheers Attitudes towards Non-drinkers Scale (CANS) [file sj-omv-5-hpq-10.1177_13591053231220519.omv › 02 descriptives/resources/a582d54fc82f1f39.png]

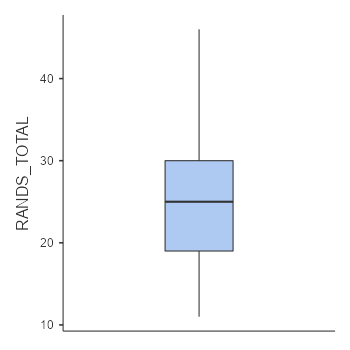

Supplement: sj-omv-5-hpq-10.1177_13591053231220519 – Supplemental material for Development and Validation of the Cheers Attitudes towards Non-drinkers Scale (CANS) [file sj-omv-5-hpq-10.1177_13591053231220519.omv › 02 descriptives/resources/a88edc0448017294.png]

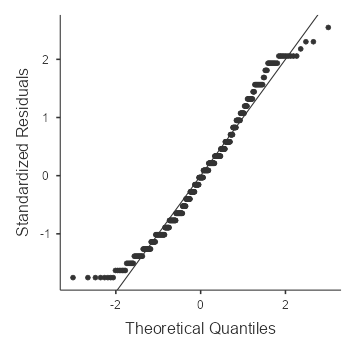

Supplement: sj-omv-5-hpq-10.1177_13591053231220519 – Supplemental material for Development and Validation of the Cheers Attitudes towards Non-drinkers Scale (CANS) [file sj-omv-5-hpq-10.1177_13591053231220519.omv › 02 descriptives/resources/4214e311aa65b788.png]

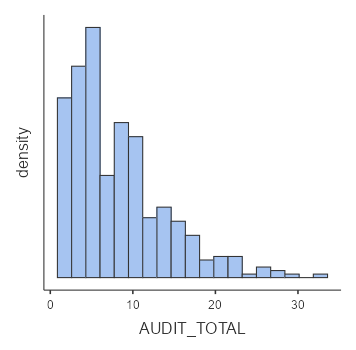

Supplement: sj-omv-5-hpq-10.1177_13591053231220519 – Supplemental material for Development and Validation of the Cheers Attitudes towards Non-drinkers Scale (CANS) [file sj-omv-5-hpq-10.1177_13591053231220519.omv › 02 descriptives/resources/0006171133fecc39.png]

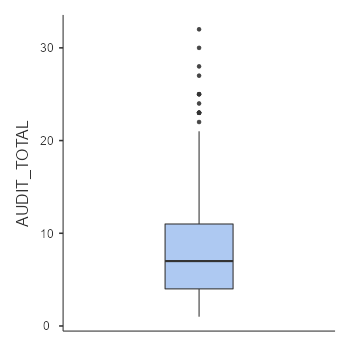

Supplement: sj-omv-5-hpq-10.1177_13591053231220519 – Supplemental material for Development and Validation of the Cheers Attitudes towards Non-drinkers Scale (CANS) [file sj-omv-5-hpq-10.1177_13591053231220519.omv › 02 descriptives/resources/7abf1c7faada79f2.png]

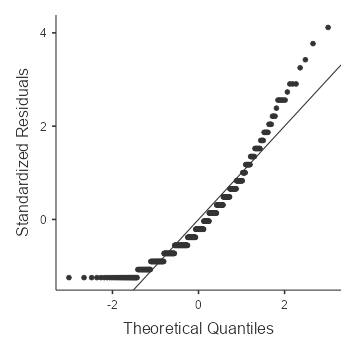

Supplement: sj-omv-5-hpq-10.1177_13591053231220519 – Supplemental material for Development and Validation of the Cheers Attitudes towards Non-drinkers Scale (CANS) [file sj-omv-5-hpq-10.1177_13591053231220519.omv › 02 descriptives/resources/359e35ce3b7dca01.png]

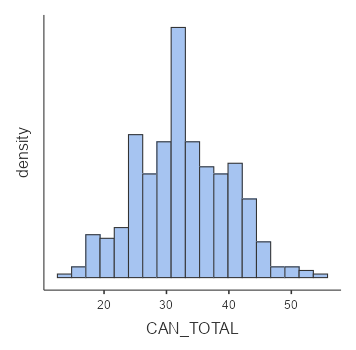

Supplement: sj-omv-5-hpq-10.1177_13591053231220519 – Supplemental material for Development and Validation of the Cheers Attitudes towards Non-drinkers Scale (CANS) [file sj-omv-5-hpq-10.1177_13591053231220519.omv › 02 descriptives/resources/d4e4ac830417f944.png]

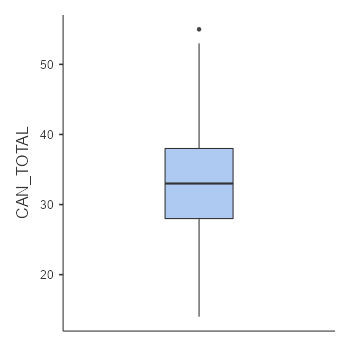

Supplement: sj-omv-5-hpq-10.1177_13591053231220519 – Supplemental material for Development and Validation of the Cheers Attitudes towards Non-drinkers Scale (CANS) [file sj-omv-5-hpq-10.1177_13591053231220519.omv › 02 descriptives/resources/ace976f66a080e1c.png]

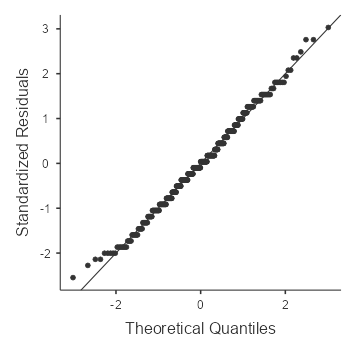

Supplement: sj-omv-5-hpq-10.1177_13591053231220519 – Supplemental material for Development and Validation of the Cheers Attitudes towards Non-drinkers Scale (CANS) [file sj-omv-5-hpq-10.1177_13591053231220519.omv › 02 descriptives/resources/fc129fac37240e42.png]

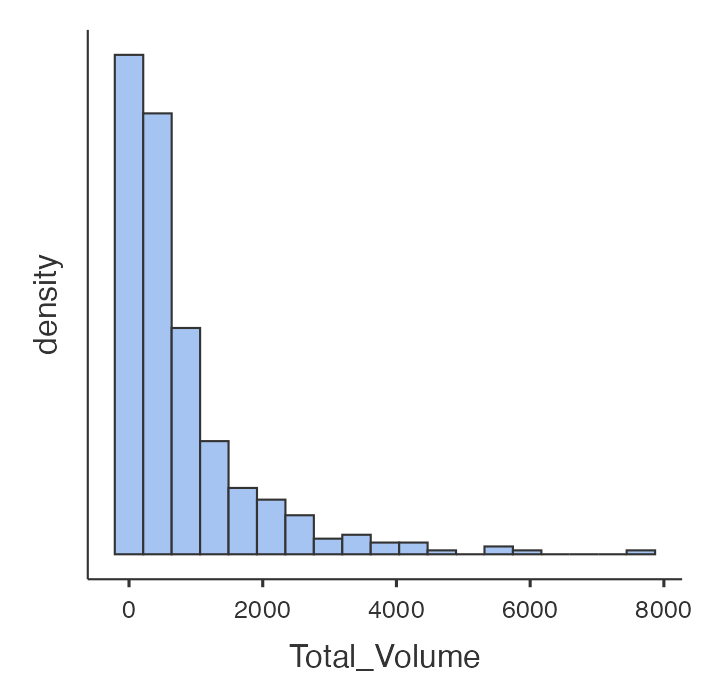

Supplement: sj-omv-5-hpq-10.1177_13591053231220519 – Supplemental material for Development and Validation of the Cheers Attitudes towards Non-drinkers Scale (CANS) [file sj-omv-5-hpq-10.1177_13591053231220519.omv › 02 descriptives/resources/203a49a1d8f2da85.png]

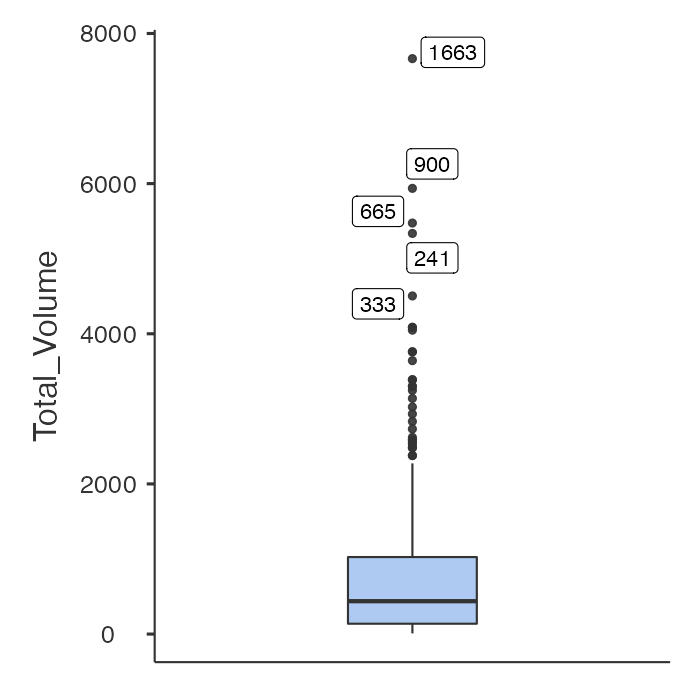

Supplement: sj-omv-5-hpq-10.1177_13591053231220519 – Supplemental material for Development and Validation of the Cheers Attitudes towards Non-drinkers Scale (CANS) [file sj-omv-5-hpq-10.1177_13591053231220519.omv › 02 descriptives/resources/1aa7c3e6970ec5ce.png]

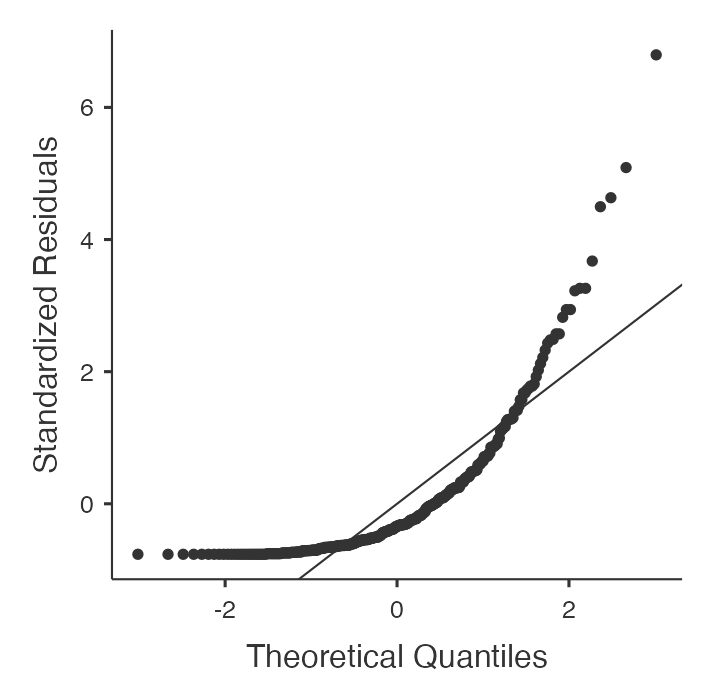

Supplement: sj-omv-5-hpq-10.1177_13591053231220519 – Supplemental material for Development and Validation of the Cheers Attitudes towards Non-drinkers Scale (CANS) [file sj-omv-5-hpq-10.1177_13591053231220519.omv › 02 descriptives/resources/5d24beb49b8cbc90.png]

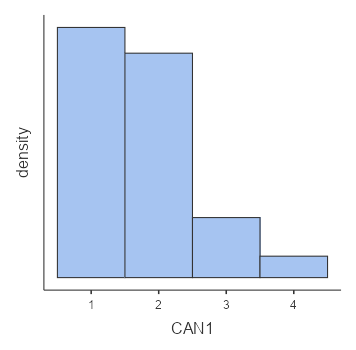

Supplement: sj-omv-5-hpq-10.1177_13591053231220519 – Supplemental material for Development and Validation of the Cheers Attitudes towards Non-drinkers Scale (CANS) [file sj-omv-5-hpq-10.1177_13591053231220519.omv › 50 descriptives/resources/983a65b8213eb30d.png]

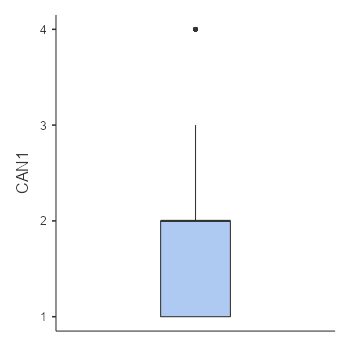

Supplement: sj-omv-5-hpq-10.1177_13591053231220519 – Supplemental material for Development and Validation of the Cheers Attitudes towards Non-drinkers Scale (CANS) [file sj-omv-5-hpq-10.1177_13591053231220519.omv › 50 descriptives/resources/bac5414cd6ceb1ac.png]

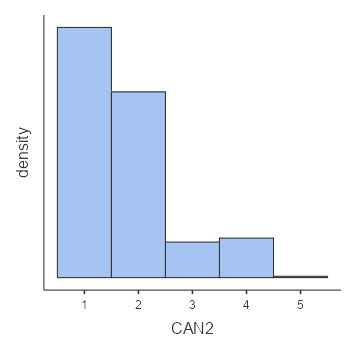

Supplement: sj-omv-5-hpq-10.1177_13591053231220519 – Supplemental material for Development and Validation of the Cheers Attitudes towards Non-drinkers Scale (CANS) [file sj-omv-5-hpq-10.1177_13591053231220519.omv › 50 descriptives/resources/973f81723650c350.png]

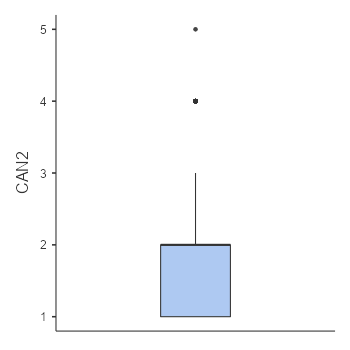

Supplement: sj-omv-5-hpq-10.1177_13591053231220519 – Supplemental material for Development and Validation of the Cheers Attitudes towards Non-drinkers Scale (CANS) [file sj-omv-5-hpq-10.1177_13591053231220519.omv › 50 descriptives/resources/592e0685705670f6.png]

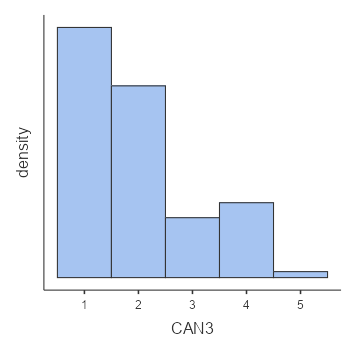

Supplement: sj-omv-5-hpq-10.1177_13591053231220519 – Supplemental material for Development and Validation of the Cheers Attitudes towards Non-drinkers Scale (CANS) [file sj-omv-5-hpq-10.1177_13591053231220519.omv › 50 descriptives/resources/4916506d30da6c9f.png]

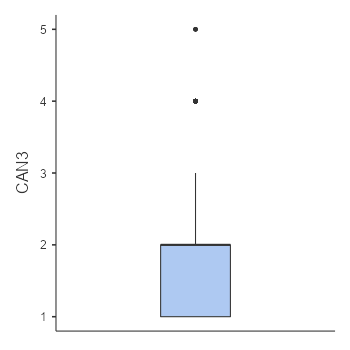

Supplement: sj-omv-5-hpq-10.1177_13591053231220519 – Supplemental material for Development and Validation of the Cheers Attitudes towards Non-drinkers Scale (CANS) [file sj-omv-5-hpq-10.1177_13591053231220519.omv › 50 descriptives/resources/67714c92b0884d10.png]

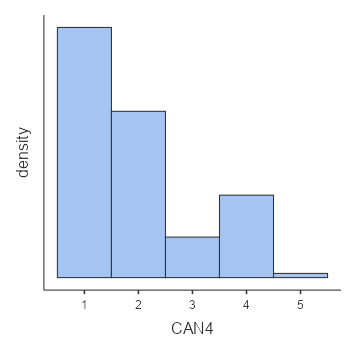

Supplement: sj-omv-5-hpq-10.1177_13591053231220519 – Supplemental material for Development and Validation of the Cheers Attitudes towards Non-drinkers Scale (CANS) [file sj-omv-5-hpq-10.1177_13591053231220519.omv › 50 descriptives/resources/23974d8f51a936a2.png]

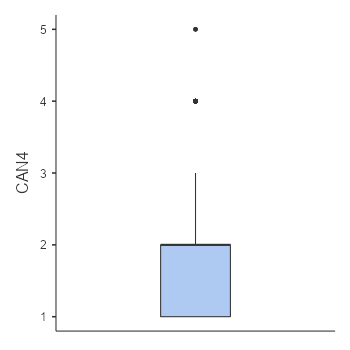

Supplement: sj-omv-5-hpq-10.1177_13591053231220519 – Supplemental material for Development and Validation of the Cheers Attitudes towards Non-drinkers Scale (CANS) [file sj-omv-5-hpq-10.1177_13591053231220519.omv › 50 descriptives/resources/41d1994ea9c22ad5.png]

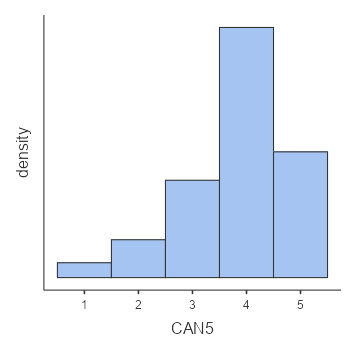

Supplement: sj-omv-5-hpq-10.1177_13591053231220519 – Supplemental material for Development and Validation of the Cheers Attitudes towards Non-drinkers Scale (CANS) [file sj-omv-5-hpq-10.1177_13591053231220519.omv › 50 descriptives/resources/2dafa617dd1edfb5.png]

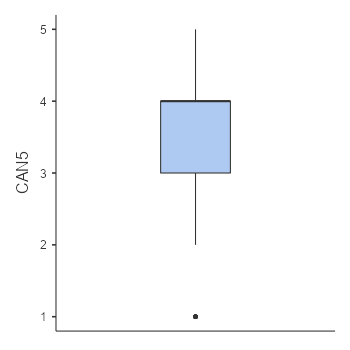

Supplement: sj-omv-5-hpq-10.1177_13591053231220519 – Supplemental material for Development and Validation of the Cheers Attitudes towards Non-drinkers Scale (CANS) [file sj-omv-5-hpq-10.1177_13591053231220519.omv › 50 descriptives/resources/1cdeb0212bc36da5.png]

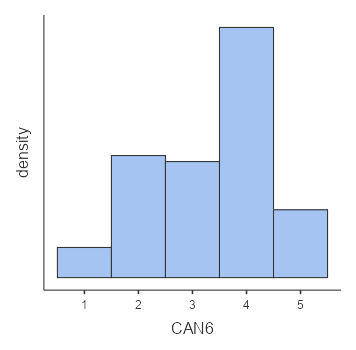

Supplement: sj-omv-5-hpq-10.1177_13591053231220519 – Supplemental material for Development and Validation of the Cheers Attitudes towards Non-drinkers Scale (CANS) [file sj-omv-5-hpq-10.1177_13591053231220519.omv › 50 descriptives/resources/b3f18f193e966312.png]

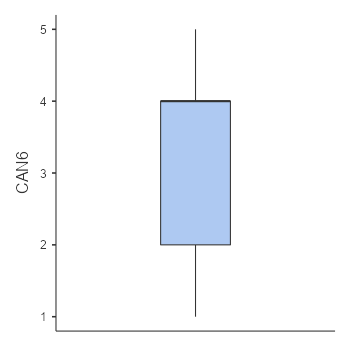

Supplement: sj-omv-5-hpq-10.1177_13591053231220519 – Supplemental material for Development and Validation of the Cheers Attitudes towards Non-drinkers Scale (CANS) [file sj-omv-5-hpq-10.1177_13591053231220519.omv › 50 descriptives/resources/c3982533ea6949cb.png]

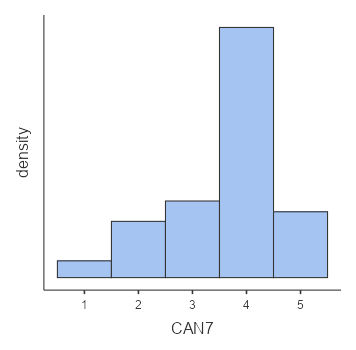

Supplement: sj-omv-5-hpq-10.1177_13591053231220519 – Supplemental material for Development and Validation of the Cheers Attitudes towards Non-drinkers Scale (CANS) [file sj-omv-5-hpq-10.1177_13591053231220519.omv › 50 descriptives/resources/4bacde075f77a34c.png]

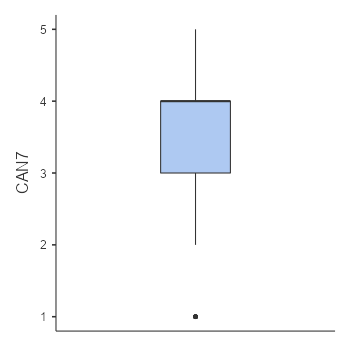

Supplement: sj-omv-5-hpq-10.1177_13591053231220519 – Supplemental material for Development and Validation of the Cheers Attitudes towards Non-drinkers Scale (CANS) [file sj-omv-5-hpq-10.1177_13591053231220519.omv › 50 descriptives/resources/b2a1b095408cd637.png]

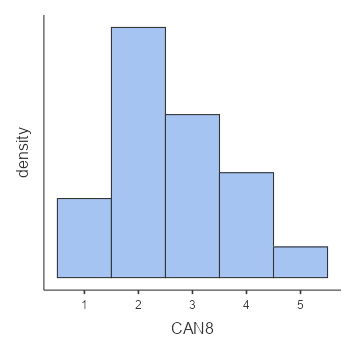

Supplement: sj-omv-5-hpq-10.1177_13591053231220519 – Supplemental material for Development and Validation of the Cheers Attitudes towards Non-drinkers Scale (CANS) [file sj-omv-5-hpq-10.1177_13591053231220519.omv › 50 descriptives/resources/6117829fabdb617f.png]

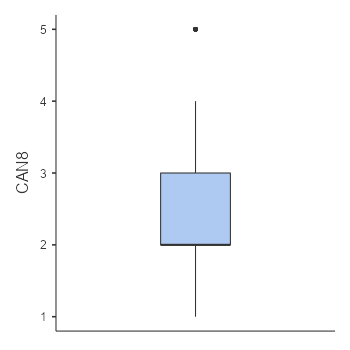

Supplement: sj-omv-5-hpq-10.1177_13591053231220519 – Supplemental material for Development and Validation of the Cheers Attitudes towards Non-drinkers Scale (CANS) [file sj-omv-5-hpq-10.1177_13591053231220519.omv › 50 descriptives/resources/fd7fa5f4c873e58e.png]

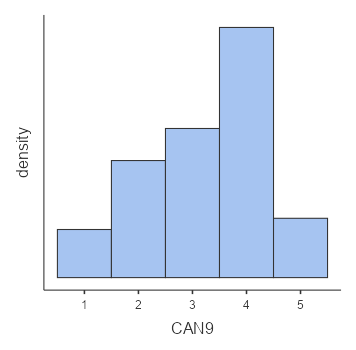

Supplement: sj-omv-5-hpq-10.1177_13591053231220519 – Supplemental material for Development and Validation of the Cheers Attitudes towards Non-drinkers Scale (CANS) [file sj-omv-5-hpq-10.1177_13591053231220519.omv › 50 descriptives/resources/46ab649a699416d5.png]

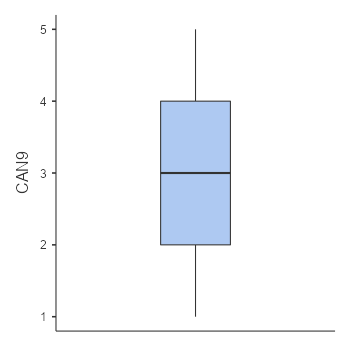

Supplement: sj-omv-5-hpq-10.1177_13591053231220519 – Supplemental material for Development and Validation of the Cheers Attitudes towards Non-drinkers Scale (CANS) [file sj-omv-5-hpq-10.1177_13591053231220519.omv › 50 descriptives/resources/42b1746b8ca9454b.png]

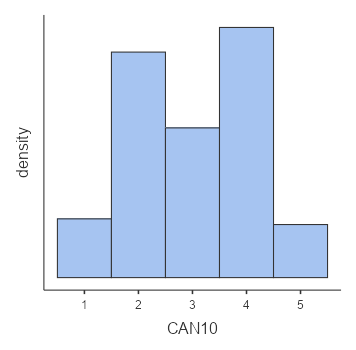

Supplement: sj-omv-5-hpq-10.1177_13591053231220519 – Supplemental material for Development and Validation of the Cheers Attitudes towards Non-drinkers Scale (CANS) [file sj-omv-5-hpq-10.1177_13591053231220519.omv › 50 descriptives/resources/b310c2bae58ea20c.png]

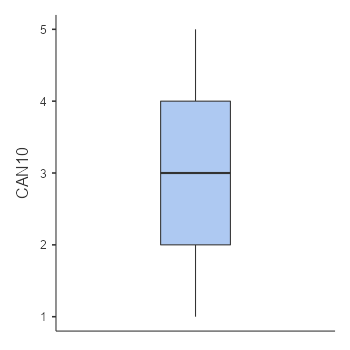

Supplement: sj-omv-5-hpq-10.1177_13591053231220519 – Supplemental material for Development and Validation of the Cheers Attitudes towards Non-drinkers Scale (CANS) [file sj-omv-5-hpq-10.1177_13591053231220519.omv › 50 descriptives/resources/d6415e795d1c9e9b.png]

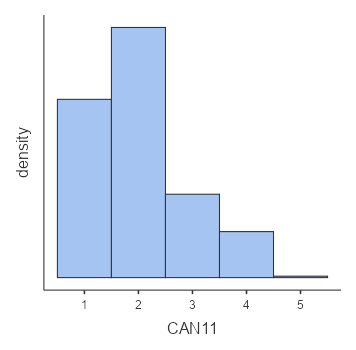

Supplement: sj-omv-5-hpq-10.1177_13591053231220519 – Supplemental material for Development and Validation of the Cheers Attitudes towards Non-drinkers Scale (CANS) [file sj-omv-5-hpq-10.1177_13591053231220519.omv › 50 descriptives/resources/1456f3fb051fa8fc.png]

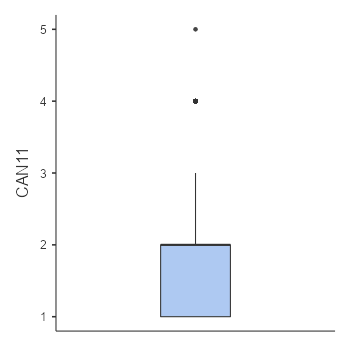

Supplement: sj-omv-5-hpq-10.1177_13591053231220519 – Supplemental material for Development and Validation of the Cheers Attitudes towards Non-drinkers Scale (CANS) [file sj-omv-5-hpq-10.1177_13591053231220519.omv › 50 descriptives/resources/7961a9960aa961c0.png]

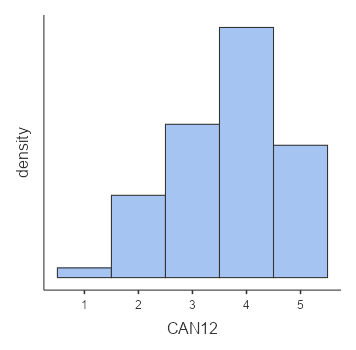

Supplement: sj-omv-5-hpq-10.1177_13591053231220519 – Supplemental material for Development and Validation of the Cheers Attitudes towards Non-drinkers Scale (CANS) [file sj-omv-5-hpq-10.1177_13591053231220519.omv › 50 descriptives/resources/2e9461a72a30576a.png]

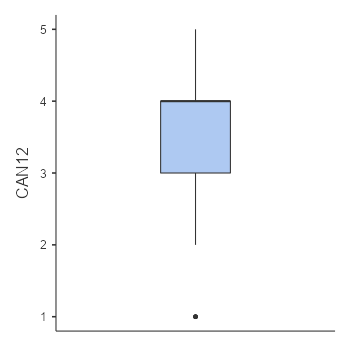

Supplement: sj-omv-5-hpq-10.1177_13591053231220519 – Supplemental material for Development and Validation of the Cheers Attitudes towards Non-drinkers Scale (CANS) [file sj-omv-5-hpq-10.1177_13591053231220519.omv › 50 descriptives/resources/4ff8bc594418842c.png]

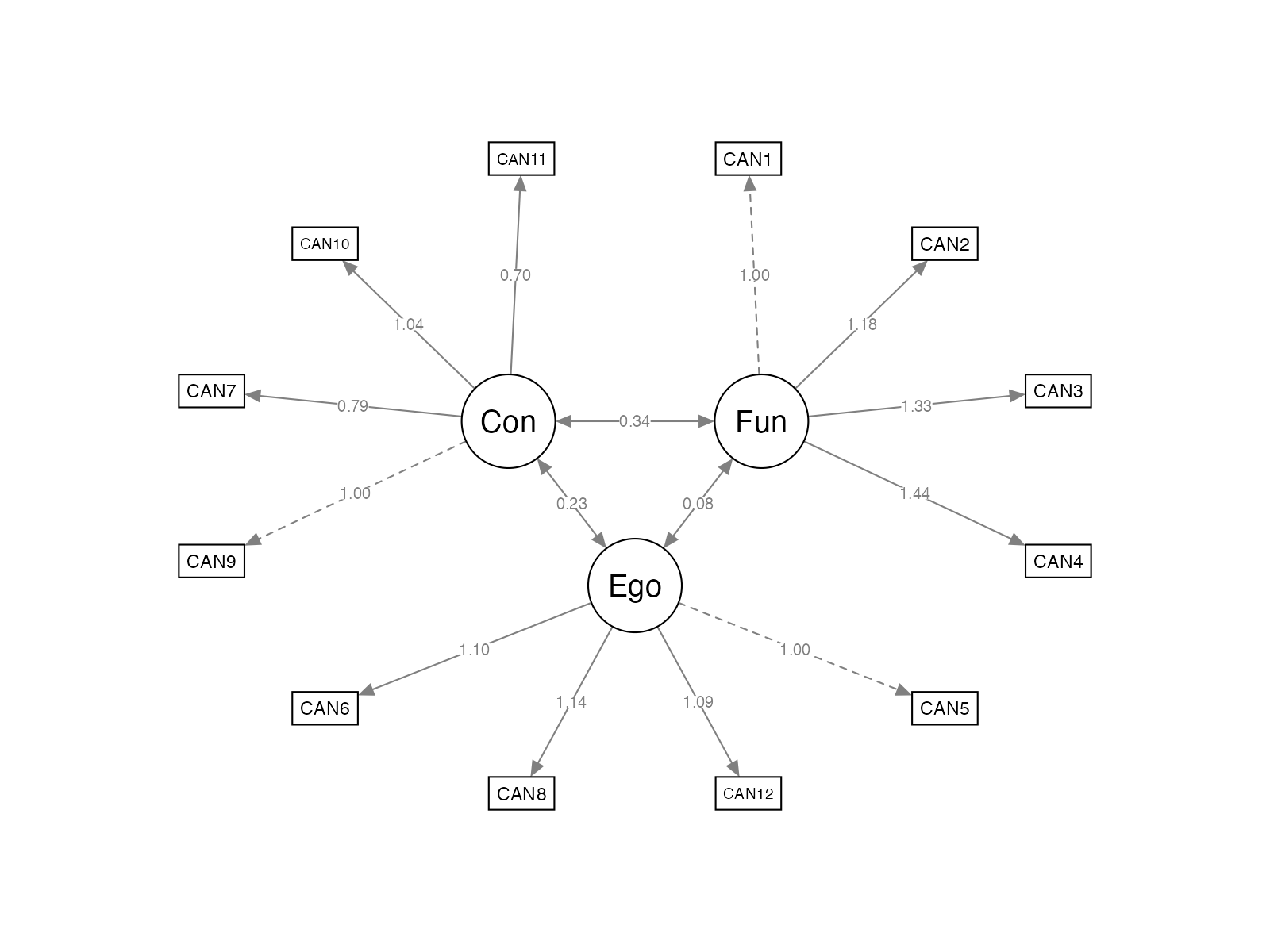

Supplement: sj-omv-5-hpq-10.1177_13591053231220519 – Supplemental material for Development and Validation of the Cheers Attitudes towards Non-drinkers Scale (CANS) [file sj-omv-5-hpq-10.1177_13591053231220519.omv › 56 semljgui/resources/4367e9b03bc55f9e.png]
