# Supplementary material for: Development and Validation of the Cheers Attitudes towards Non-drinkers Scale (CANS)
Source: J Health Psychol. 2024 Jan 29;29(10):1101–14. doi: 10.1177/13591053231220519 (PMC11344955; doi:10.1177/13591053231220519)
Supplement: sj-omv-5-hpq-10.1177_13591053231220519 – Supplemental material for Development and Validation of the Cheers Attitudes towards Non-drinkers Scale (CANS) [file sj-omv-5-hpq-10.1177_13591053231220519.omv › index.html]

Results


# Results

# Descriptives

| Descriptives | | | | | | | | | | | |
| --- | --- | --- | --- | --- | --- | --- | --- | --- | --- | --- | --- |
|  | | Age | | RANDS\_TOTAL | | AUDIT\_TOTAL | | CAN\_TOTAL | | Total\_Volume | |
| N |  | 389 |  | 383 |  | 385 |  | 389 |  | 389 |  |
| Missing |  | 0 |  | 6 |  | 4 |  | 0 |  | 0 |  |
| Mean |  | 39.8 |  | 25.3 |  | 8.21 |  | 32.7 |  | 783 |  |
| Median |  | 36.0 |  | 25.0 |  | 7.00 |  | 33.0 |  | 438 |  |
| Standard deviation |  | 13.5 |  | 8.14 |  | 5.78 |  | 7.35 |  | 1013 |  |
| Variance |  | 181 |  | 66.2 |  | 33.4 |  | 54.0 |  | 1.03e+6 |  |
| Range |  | 52.0 |  | 35.0 |  | 31.0 |  | 41.0 |  | 7656 |  |
| Minimum |  | 18.0 |  | 11.0 |  | 1.00 |  | 14.0 |  | 9.00 |  |
| Maximum |  | 70.0 |  | 46.0 |  | 32.0 |  | 55.0 |  | 7665 |  |
| Skewness |  | 0.580 |  | 0.340 |  | 1.19 |  | 0.127 |  | 2.66 |  |
| Std. error skewness |  | 0.124 |  | 0.125 |  | 0.124 |  | 0.124 |  | 0.124 |  |
| Kurtosis |  | -0.629 |  | -0.621 |  | 1.49 |  | -0.183 |  | 9.72 |  |
| Std. error kurtosis |  | 0.247 |  | 0.249 |  | 0.248 |  | 0.247 |  | 0.247 |  |
| Shapiro-Wilk W |  | 0.939 |  | 0.974 |  | 0.908 |  | 0.994 |  | 0.715 |  |
| Shapiro-Wilk p |  | < .001 |  | < .001 |  | < .001 |  | 0.151 |  | < .001 |  |
|  |  |  |  |  |  |  |  |  |  |  |  |
| --- | --- | --- | --- | --- | --- | --- | --- | --- | --- | --- | --- |
|  | | | | | | | | | | | |
|  | | | | | | | | | | | |

## Plots

### Age

### RANDS\_TOTAL

### AUDIT\_TOTAL

### CAN\_TOTAL

### Total\_Volume

# Descriptives

## Frequencies

| Frequencies of Location | | | | | | | |
| --- | --- | --- | --- | --- | --- | --- | --- |
| Location | | Counts | | % of Total | | Cumulative % | |
| ACT |  | 9 |  | 2.3 % |  | 2.3 % |  |
| NSW |  | 102 |  | 26.2 % |  | 28.5 % |  |
| NT |  | 3 |  | 0.8 % |  | 29.3 % |  |
| QLD |  | 33 |  | 8.5 % |  | 37.8 % |  |
| SA |  | 19 |  | 4.9 % |  | 42.7 % |  |
| TAS |  | 4 |  | 1.0 % |  | 43.7 % |  |
| VIC |  | 194 |  | 49.9 % |  | 93.6 % |  |
| WA |  | 25 |  | 6.4 % |  | 100.0 % |  |
|  |  |  |  |  |  |  |  |
| --- | --- | --- | --- | --- | --- | --- | --- |
|  | | | | | | | |
|  | | | | | | | |

| Frequencies of Gender | | | | | | | |
| --- | --- | --- | --- | --- | --- | --- | --- |
| Gender | | Counts | | % of Total | | Cumulative % | |
| Male (including transgender men) |  | 169 |  | 43.4 % |  | 43.4 % |  |
| Female (including transgender women) |  | 211 |  | 54.2 % |  | 97.7 % |  |
| Prefer not to say |  | 2 |  | 0.5 % |  | 98.2 % |  |
| Prefer to self-describe as \_\_\_\_\_\_\_\_\_ (e.g. non-binary, gende |  | 7 |  | 1.8 % |  | 100.0 % |  |
|  |  |  |  |  |  |  |  |
| --- | --- | --- | --- | --- | --- | --- | --- |
|  | | | | | | | |
|  | | | | | | | |

| Frequencies of Gender\_other | | | | | | | |
| --- | --- | --- | --- | --- | --- | --- | --- |
| Gender\_other | | Counts | | % of Total | | Cumulative % | |
| queer |  | 1 |  | 9.1 % |  | 9.1 % |  |
| Demigirl |  | 2 |  | 18.2 % |  | 27.3 % |  |
| non-binary |  | 1 |  | 9.1 % |  | 36.4 % |  |
| Non-binary |  | 2 |  | 18.2 % |  | 54.5 % |  |
| Non binary |  | 3 |  | 27.3 % |  | 81.8 % |  |
| Biological female |  | 1 |  | 9.1 % |  | 90.9 % |  |
| A person of female sex with non-binary gender |  | 1 |  | 9.1 % |  | 100.0 % |  |
|  |  |  |  |  |  |  |  |
| --- | --- | --- | --- | --- | --- | --- | --- |
|  | | | | | | | |
|  | | | | | | | |

| Frequencies of Nationality | | | | | | | |
| --- | --- | --- | --- | --- | --- | --- | --- |
| Nationality | | Counts | | % of Total | | Cumulative % | |
| Australian |  | 356 |  | 91.5 % |  | 91.5 % |  |
| Other (please click to specify) |  | 33 |  | 8.5 % |  | 100.0 % |  |
|  |  |  |  |  |  |  |  |
| --- | --- | --- | --- | --- | --- | --- | --- |
|  | | | | | | | |
|  | | | | | | | |

# Correlation Matrix

| Correlation Matrix | | | | | | | | | | | | | | | | | | | | | |
| --- | --- | --- | --- | --- | --- | --- | --- | --- | --- | --- | --- | --- | --- | --- | --- | --- | --- | --- | --- | --- | --- |
|  | |  | | CAN\_TOTAL | | Fun\_CAN | | Ego\_CAN | | Connection\_CAN | | RANDS\_TOTAL | | AUDIT\_TOTAL | | Total\_Volume | | Age | | Gender MF | |
| CAN\_TOTAL |  | Spearman's rho |  | — |  |  |  |  |  |  |  |  |  |  |  |  |  |  |  |  |  |
|  |  | p-value |  | — |  |  |  |  |  |  |  |  |  |  |  |  |  |  |  |  |  |
|  |  | N |  | — |  |  |  |  |  |  |  |  |  |  |  |  |  |  |  |  |  |
| Fun\_CAN |  | Spearman's rho |  | 0.748 | \*\*\* | — |  |  |  |  |  |  |  |  |  |  |  |  |  |  |  |
|  |  | p-value |  | < .001 |  | — |  |  |  |  |  |  |  |  |  |  |  |  |  |  |  |
|  |  | N |  | 389 |  | — |  |  |  |  |  |  |  |  |  |  |  |  |  |  |  |
| Ego\_CAN |  | Spearman's rho |  | 0.656 | \*\*\* | 0.185 | \*\*\* | — |  |  |  |  |  |  |  |  |  |  |  |  |  |
|  |  | p-value |  | < .001 |  | < .001 |  | — |  |  |  |  |  |  |  |  |  |  |  |  |  |
|  |  | N |  | 389 |  | 389 |  | — |  |  |  |  |  |  |  |  |  |  |  |  |  |
| Connection\_CAN |  | Spearman's rho |  | 0.828 | \*\*\* | 0.552 | \*\*\* | 0.322 | \*\*\* | — |  |  |  |  |  |  |  |  |  |  |  |
|  |  | p-value |  | < .001 |  | < .001 |  | < .001 |  | — |  |  |  |  |  |  |  |  |  |  |  |
|  |  | N |  | 389 |  | 389 |  | 389 |  | — |  |  |  |  |  |  |  |  |  |  |  |
| RANDS\_TOTAL |  | Spearman's rho |  | 0.677 | \*\*\* | 0.734 | \*\*\* | 0.195 | \*\*\* | 0.607 | \*\*\* | — |  |  |  |  |  |  |  |  |  |
|  |  | p-value |  | < .001 |  | < .001 |  | < .001 |  | < .001 |  | — |  |  |  |  |  |  |  |  |  |
|  |  | N |  | 383 |  | 383 |  | 383 |  | 383 |  | — |  |  |  |  |  |  |  |  |  |
| AUDIT\_TOTAL |  | Spearman's rho |  | 0.542 | \*\*\* | 0.419 | \*\*\* | 0.380 | \*\*\* | 0.429 | \*\*\* | 0.506 | \*\*\* | — |  |  |  |  |  |  |  |
|  |  | p-value |  | < .001 |  | < .001 |  | < .001 |  | < .001 |  | < .001 |  | — |  |  |  |  |  |  |  |
|  |  | N |  | 385 |  | 385 |  | 385 |  | 385 |  | 381 |  | — |  |  |  |  |  |  |  |
| Total\_Volume |  | Spearman's rho |  | 0.386 | \*\*\* | 0.318 | \*\*\* | 0.254 | \*\*\* | 0.288 | \*\*\* | 0.427 | \*\*\* | 0.807 | \*\*\* | — |  |  |  |  |  |
|  |  | p-value |  | < .001 |  | < .001 |  | < .001 |  | < .001 |  | < .001 |  | < .001 |  | — |  |  |  |  |  |
|  |  | N |  | 389 |  | 389 |  | 389 |  | 389 |  | 383 |  | 385 |  | — |  |  |  |  |  |
| Age |  | Spearman's rho |  | -0.251 | \*\*\* | -0.163 | \*\* | -0.079 |  | -0.330 | \*\*\* | -0.194 | \*\*\* | -0.192 | \*\*\* | -0.111 | \* | — |  |  |  |
|  |  | p-value |  | < .001 |  | 0.001 |  | 0.118 |  | < .001 |  | < .001 |  | < .001 |  | 0.028 |  | — |  |  |  |
|  |  | N |  | 389 |  | 389 |  | 389 |  | 389 |  | 383 |  | 385 |  | 389 |  | — |  |  |  |
| Gender MF |  | Spearman's rho |  | -0.129 | \* | -0.123 | \* | -0.087 |  | -0.096 |  | -0.144 | \*\* | -0.251 | \*\*\* | -0.292 | \*\*\* | -0.033 |  | — |  |
|  |  | p-value |  | 0.012 |  | 0.016 |  | 0.091 |  | 0.062 |  | 0.005 |  | < .001 |  | < .001 |  | 0.522 |  | — |  |
|  |  | N |  | 380 |  | 380 |  | 380 |  | 380 |  | 375 |  | 378 |  | 380 |  | 380 |  | — |  |
|  |  |  |  |  |  |  |  |  |  |  |  |  |  |  |  |  |  |  |  |  |  |
| --- | --- | --- | --- | --- | --- | --- | --- | --- | --- | --- | --- | --- | --- | --- | --- | --- | --- | --- | --- | --- | --- |
| Note. \* p < .05, \*\* p < .01, \*\*\* p < .001 | | | | | | | | | | | | | | | | | | | | | |
|  | | | | | | | | | | | | | | | | | | | | | |
|  | | | | | | | | | | | | | | | | | | | | | |

# Reliability Analysis

| Scale Reliability Statistics | | | |
| --- | --- | --- | --- |
|  | | Cronbach's α | |
| scale |  | 0.842 |  |
|  |  |  |  |
| --- | --- | --- | --- |
|  | | | |
| [3] | | | |

| Item Reliability Statistics | | | | | |
| --- | --- | --- | --- | --- | --- |
|  | | | | if item dropped | |
|  | | item-rest correlation | | Cronbach's α | |
| CAN2 |  | 0.537 |  | 0.828 |  |
| CAN3 |  | 0.553 |  | 0.826 |  |
| CAN4 |  | 0.566 |  | 0.825 |  |
| CAN5 |  | 0.368 |  | 0.839 |  |
| CAN6 |  | 0.348 |  | 0.842 |  |
| CAN7 |  | 0.589 |  | 0.824 |  |
| CAN8 |  | 0.424 |  | 0.836 |  |
| CAN9 |  | 0.636 |  | 0.819 |  |
| CAN10 |  | 0.633 |  | 0.819 |  |
| CAN12 |  | 0.406 |  | 0.837 |  |
| CAN1 |  | 0.480 |  | 0.832 |  |
| CAN11 |  | 0.549 |  | 0.827 |  |
|  |  |  |  |  |  |
| --- | --- | --- | --- | --- | --- |
|  | | | | | |
|  | | | | | |

# Reliability Analysis

| Scale Reliability Statistics | | | |
| --- | --- | --- | --- |
|  | | Cronbach's α | |
| scale |  | 0.830 |  |
|  |  |  |  |
| --- | --- | --- | --- |
|  | | | |
| [3] | | | |

| Item Reliability Statistics | | | | | |
| --- | --- | --- | --- | --- | --- |
|  | | | | if item dropped | |
|  | | item-rest correlation | | Cronbach's α | |
| CAN1 |  | 0.693 |  | 0.780 |  |
| CAN2 |  | 0.716 |  | 0.763 |  |
| CAN3 |  | 0.642 |  | 0.796 |  |
| CAN4 |  | 0.625 |  | 0.807 |  |
|  |  |  |  |  |  |
| --- | --- | --- | --- | --- | --- |
|  | | | | | |
|  | | | | | |

# Reliability Analysis

| Scale Reliability Statistics | | | |
| --- | --- | --- | --- |
|  | | Cronbach's α | |
| scale |  | 0.761 |  |
|  |  |  |  |
| --- | --- | --- | --- |
|  | | | |
| [3] | | | |

| Item Reliability Statistics | | | | | |
| --- | --- | --- | --- | --- | --- |
|  | | | | if item dropped | |
|  | | item-rest correlation | | Cronbach's α | |
| CAN5 |  | 0.567 |  | 0.701 |  |
| CAN6 |  | 0.573 |  | 0.697 |  |
| CAN8 |  | 0.537 |  | 0.716 |  |
| CAN12 |  | 0.561 |  | 0.703 |  |
|  |  |  |  |  |  |
| --- | --- | --- | --- | --- | --- |
|  | | | | | |
|  | | | | | |

# Reliability Analysis

| Scale Reliability Statistics | | | |
| --- | --- | --- | --- |
|  | | Cronbach's α | |
| scale |  | 0.835 |  |
|  |  |  |  |
| --- | --- | --- | --- |
|  | | | |
| [3] | | | |

| Item Reliability Statistics | | | | | |
| --- | --- | --- | --- | --- | --- |
|  | | | | if item dropped | |
|  | | item-rest correlation | | Cronbach's α | |
| CAN11 |  | 0.551 |  | 0.838 |  |
| CAN9 |  | 0.748 |  | 0.752 |  |
| CAN10 |  | 0.742 |  | 0.756 |  |
| CAN7 |  | 0.634 |  | 0.805 |  |
|  |  |  |  |  |  |
| --- | --- | --- | --- | --- | --- |
|  | | | | | |
|  | | | | | |

# Correlation Matrix

| Correlation Matrix | | | | | | | | | |
| --- | --- | --- | --- | --- | --- | --- | --- | --- | --- |
|  | |  | | Fun\_CAN | | Ego\_CAN | | Connection\_CAN | |
| Fun\_CAN |  | Pearson's r |  | — |  |  |  |  |  |
|  |  | p-value |  | — |  |  |  |  |  |
| Ego\_CAN |  | Pearson's r |  | 0.183 | \*\*\* | — |  |  |  |
|  |  | p-value |  | < .001 |  | — |  |  |  |
| Connection\_CAN |  | Pearson's r |  | 0.555 | \*\*\* | 0.327 | \*\*\* | — |  |
|  |  | p-value |  | < .001 |  | < .001 |  | — |  |
|  |  |  |  |  |  |  |  |  |  |
| --- | --- | --- | --- | --- | --- | --- | --- | --- | --- |
| Note. \* p < .05, \*\* p < .01, \*\*\* p < .001 | | | | | | | | | |
|  | | | | | | | | | |
|  | | | | | | | | | |

# Descriptives

| Descriptives | | | | | | | | | | | | | | | | | | | | | | | | | |
| --- | --- | --- | --- | --- | --- | --- | --- | --- | --- | --- | --- | --- | --- | --- | --- | --- | --- | --- | --- | --- | --- | --- | --- | --- | --- |
|  | | CAN1 | | CAN2 | | CAN3 | | CAN4 | | CAN5 | | CAN6 | | CAN7 | | CAN8 | | CAN9 | | CAN10 | | CAN11 | | CAN12 | |
| N |  | 389 |  | 389 |  | 389 |  | 389 |  | 389 |  | 389 |  | 389 |  | 389 |  | 389 |  | 389 |  | 389 |  | 389 |  |
| Missing |  | 0 |  | 0 |  | 0 |  | 0 |  | 0 |  | 0 |  | 0 |  | 0 |  | 0 |  | 0 |  | 0 |  | 0 |  |
| Mean |  | 1.74 |  | 1.74 |  | 1.96 |  | 1.94 |  | 3.83 |  | 3.35 |  | 3.63 |  | 2.61 |  | 3.25 |  | 3.02 |  | 2.00 |  | 3.66 |  |
| Median |  | 2 |  | 2 |  | 2 |  | 2 |  | 4 |  | 4 |  | 4 |  | 2 |  | 3 |  | 3 |  | 2 |  | 4 |  |
| Standard deviation |  | 0.802 |  | 0.909 |  | 1.07 |  | 1.10 |  | 0.969 |  | 1.09 |  | 0.988 |  | 1.06 |  | 1.10 |  | 1.12 |  | 0.907 |  | 1.00 |  |
| Minimum |  | 1 |  | 1 |  | 1 |  | 1 |  | 1 |  | 1 |  | 1 |  | 1 |  | 1 |  | 1 |  | 1 |  | 1 |  |
| Maximum |  | 4 |  | 5 |  | 5 |  | 5 |  | 5 |  | 5 |  | 5 |  | 5 |  | 5 |  | 5 |  | 5 |  | 5 |  |
| Skewness |  | 0.966 |  | 1.24 |  | 0.920 |  | 0.946 |  | -0.875 |  | -0.422 |  | -0.856 |  | 0.426 |  | -0.425 |  | -0.0687 |  | 0.746 |  | -0.432 |  |
| Std. error skewness |  | 0.124 |  | 0.124 |  | 0.124 |  | 0.124 |  | 0.124 |  | 0.124 |  | 0.124 |  | 0.124 |  | 0.124 |  | 0.124 |  | 0.124 |  | 0.124 |  |
| Kurtosis |  | 0.477 |  | 0.883 |  | -0.218 |  | -0.343 |  | 0.609 |  | -0.714 |  | 0.285 |  | -0.489 |  | -0.656 |  | -1.03 |  | -0.0309 |  | -0.502 |  |
| Std. error kurtosis |  | 0.247 |  | 0.247 |  | 0.247 |  | 0.247 |  | 0.247 |  | 0.247 |  | 0.247 |  | 0.247 |  | 0.247 |  | 0.247 |  | 0.247 |  | 0.247 |  |
|  |  |  |  |  |  |  |  |  |  |  |  |  |  |  |  |  |  |  |  |  |  |  |  |  |  |
| --- | --- | --- | --- | --- | --- | --- | --- | --- | --- | --- | --- | --- | --- | --- | --- | --- | --- | --- | --- | --- | --- | --- | --- | --- | --- |
|  | | | | | | | | | | | | | | | | | | | | | | | | | |
|  | | | | | | | | | | | | | | | | | | | | | | | | | |

## Plots

### CAN1

### CAN2

### CAN3

### CAN4

### CAN5

### CAN6

### CAN7

### CAN8

### CAN9

### CAN10

### CAN11

### CAN12

# Confirmatory Factor Analysis

| Models Info | | | | | |
| --- | --- | --- | --- | --- | --- |
|  | |  | |  | |
| Estimation Method |  | DWLS |  | . |  |
| Optimization Method |  | NLMINB |  |  |  |
| Number of observations |  | 389 |  |  |  |
| Free parameters |  | 39 |  |  |  |
| Standard errors |  | Robust |  |  |  |
| Scaled test |  | Satorra-Bentler mean adjusted |  |  |  |
| Converged |  | TRUE |  |  |  |
| Iterations |  | 39 |  |  |  |
|  |  |  |  |  |  |
| Model |  | Fun=~CAN1+CAN2+CAN3+CAN4 |  |  |  |
|  |  | Ego=~CAN5+CAN12+CAN8+CAN6 |  |  |  |
|  |  | Con=~CAN9+CAN7+CAN10+CAN11 |  |  |  |
|  |  |  |  |  |  |
|  |  |  |  |  |  |
|  |  |  |  |  |  |
| --- | --- | --- | --- | --- | --- |
|  | | | | | |
| [4] [5] | | | | | |

## Overall Tests

| Model tests | | | | | | | |
| --- | --- | --- | --- | --- | --- | --- | --- |
| Label | | X² | | df | | p | |
| User Model |  | 85.4 |  | 51 |  | 0.002 |  |
| Baseline Model |  | 2718.9 |  | 66 |  | < .001 |  |
| Scaled User |  | 201.6 |  | 51 |  | < .001 |  |
| Scaled Baseline |  | 2718.9 |  | 66 |  | < .001 |  |
|  |  |  |  |  |  |  |  |
| --- | --- | --- | --- | --- | --- | --- | --- |
|  | | | | | | | |
|  | | | | | | | |

| Fit indices | | | | | | | | | | | |
| --- | --- | --- | --- | --- | --- | --- | --- | --- | --- | --- | --- |
|  | | | | | | 95% Confidence Intervals | | | |  | |
| Type | | SRMR | | RMSEA | | Lower | | Upper | | RMSEA p | |
| Classical |  | 0.054 |  | 0.042 |  | 0.025 |  | 0.057 |  | 0.805 |  |
| Robust |  | 0.054 |  | 0.057 |  | 0.049 |  | 0.065 |  |  |  |
| Scaled |  | 0.054 |  | 0.087 |  | 0.068 |  | 0.107 |  | < .001 |  |
|  |  |  |  |  |  |  |  |  |  |  |  |
| --- | --- | --- | --- | --- | --- | --- | --- | --- | --- | --- | --- |
|  | | | | | | | | | | | |
|  | | | | | | | | | | | |

| User model versus baseline model | | | |
| --- | --- | --- | --- |
|  | | Model | |
| Comparative Fit Index (CFI) |  | 0.987 |  |
| Tucker-Lewis Index (TLI) |  | 0.983 |  |
| Bentler-Bonett Non-normed Fit Index (NNFI) |  | 0.983 |  |
| Bentler-Bonett Normed Fit Index (NFI) |  | 0.969 |  |
| Parsimony Normed Fit Index (PNFI) |  | 0.748 |  |
| Bollen's Relative Fit Index (RFI) |  | 0.959 |  |
| Bollen's Incremental Fit Index (IFI) |  | 0.987 |  |
| Relative Noncentrality Index (RNI) |  | 0.987 |  |
|  |  |  |  |
| --- | --- | --- | --- |
|  | | | |
|  | | | |

| Additional fit indices | | | |
| --- | --- | --- | --- |
|  | | Model | |
| Hoelter Critical N (CN), a=0.05 |  | 313.006 |  |
| Hoelter Critical N (CN), a=0.01 |  | 352.611 |  |
| Goodness of Fit Index (GFI) |  | 0.998 |  |
| Parsimony Goodness of Fit Index (GFI) |  | 0.566 |  |
| McDonald Fit Index (MFI) |  | 0.957 |  |
|  |  |  |  |
| --- | --- | --- | --- |
|  | | | |
|  | | | |

## Estimates

| Measurement model | | | | | | | | | | | | | | | | | |
| --- | --- | --- | --- | --- | --- | --- | --- | --- | --- | --- | --- | --- | --- | --- | --- | --- | --- |
|  | | | | | | | | 95% Confidence Intervals | | | |  | | | | | |
| Latent | | Observed | | Estimate | | SE | | Lower | | Upper | | β | | z | | p | |
| Fun |  | CAN1 |  | 1.000 |  | 0.0000 |  | 1.000 |  | 1.000 |  | 0.733 |  |  |  |  |  |
|  |  | CAN2 |  | 1.178 |  | 0.0968 |  | 0.988 |  | 1.368 |  | 0.763 |  | 12.17 |  | < .001 |  |
|  |  | CAN3 |  | 1.336 |  | 0.1324 |  | 1.077 |  | 1.596 |  | 0.736 |  | 10.10 |  | < .001 |  |
|  |  | CAN4 |  | 1.426 |  | 0.1098 |  | 1.210 |  | 1.641 |  | 0.762 |  | 12.98 |  | < .001 |  |
| Ego |  | CAN5 |  | 1.000 |  | 0.0000 |  | 1.000 |  | 1.000 |  | 0.629 |  |  |  |  |  |
|  |  | CAN12 |  | 1.121 |  | 0.1358 |  | 0.855 |  | 1.387 |  | 0.682 |  | 8.26 |  | < .001 |  |
|  |  | CAN8 |  | 1.221 |  | 0.1687 |  | 0.891 |  | 1.552 |  | 0.703 |  | 7.24 |  | < .001 |  |
|  |  | CAN6 |  | 1.115 |  | 0.1449 |  | 0.831 |  | 1.399 |  | 0.624 |  | 7.69 |  | < .001 |  |
| Con |  | CAN9 |  | 1.000 |  | 0.0000 |  | 1.000 |  | 1.000 |  | 0.790 |  |  |  |  |  |
|  |  | CAN7 |  | 0.790 |  | 0.0547 |  | 0.683 |  | 0.898 |  | 0.697 |  | 14.44 |  | < .001 |  |
|  |  | CAN10 |  | 1.043 |  | 0.0550 |  | 0.935 |  | 1.151 |  | 0.812 |  | 18.97 |  | < .001 |  |
|  |  | CAN11 |  | 0.704 |  | 0.0598 |  | 0.587 |  | 0.821 |  | 0.677 |  | 11.77 |  | < .001 |  |
|  |  |  |  |  |  |  |  |  |  |  |  |  |  |  |  |  |  |
| --- | --- | --- | --- | --- | --- | --- | --- | --- | --- | --- | --- | --- | --- | --- | --- | --- | --- |
|  | | | | | | | | | | | | | | | | | |
|  | | | | | | | | | | | | | | | | | |

| Variances and Covariances | | | | | | | | | | | | | | | | | |
| --- | --- | --- | --- | --- | --- | --- | --- | --- | --- | --- | --- | --- | --- | --- | --- | --- | --- |
|  | | | | | | | | 95% Confidence Intervals | | | |  | | | | | |
| Variable 1 | | Variable 2 | | Estimate | | SE | | Lower | | Upper | | β | | z | | p | |
| CAN1 |  | CAN1 |  | 0.2976 |  | 0.0366 |  | 0.2258 |  | 0.369 |  | 0.462 |  | 8.13 |  | < .001 |  |
| CAN2 |  | CAN2 |  | 0.3451 |  | 0.0487 |  | 0.2496 |  | 0.441 |  | 0.418 |  | 7.08 |  | < .001 |  |
| CAN3 |  | CAN3 |  | 0.5222 |  | 0.0640 |  | 0.3968 |  | 0.648 |  | 0.458 |  | 8.16 |  | < .001 |  |
| CAN4 |  | CAN4 |  | 0.5070 |  | 0.0630 |  | 0.3836 |  | 0.630 |  | 0.419 |  | 8.05 |  | < .001 |  |
| CAN5 |  | CAN5 |  | 0.5672 |  | 0.0673 |  | 0.4354 |  | 0.699 |  | 0.604 |  | 8.43 |  | < .001 |  |
| CAN12 |  | CAN12 |  | 0.5373 |  | 0.0673 |  | 0.4054 |  | 0.669 |  | 0.535 |  | 7.98 |  | < .001 |  |
| CAN8 |  | CAN8 |  | 0.5653 |  | 0.0675 |  | 0.4330 |  | 0.698 |  | 0.505 |  | 8.38 |  | < .001 |  |
| CAN6 |  | CAN6 |  | 0.7247 |  | 0.0714 |  | 0.5848 |  | 0.865 |  | 0.611 |  | 10.16 |  | < .001 |  |
| CAN9 |  | CAN9 |  | 0.4580 |  | 0.0479 |  | 0.3642 |  | 0.552 |  | 0.376 |  | 9.56 |  | < .001 |  |
| CAN7 |  | CAN7 |  | 0.5016 |  | 0.0448 |  | 0.4139 |  | 0.589 |  | 0.514 |  | 11.21 |  | < .001 |  |
| CAN10 |  | CAN10 |  | 0.4277 |  | 0.0510 |  | 0.3277 |  | 0.528 |  | 0.341 |  | 8.38 |  | < .001 |  |
| CAN11 |  | CAN11 |  | 0.4450 |  | 0.0423 |  | 0.3621 |  | 0.528 |  | 0.541 |  | 10.52 |  | < .001 |  |
| Fun |  | Fun |  | 0.3461 |  | 0.0501 |  | 0.2480 |  | 0.444 |  | 1.000 |  | 6.91 |  | < .001 |  |
| Ego |  | Ego |  | 0.3712 |  | 0.0736 |  | 0.2270 |  | 0.515 |  | 1.000 |  | 5.04 |  | < .001 |  |
| Con |  | Con |  | 0.7606 |  | 0.0806 |  | 0.6027 |  | 0.918 |  | 1.000 |  | 9.44 |  | < .001 |  |
| Fun |  | Ego |  | 0.0811 |  | 0.0255 |  | 0.0311 |  | 0.131 |  | 0.226 |  | 3.18 |  | 0.001 |  |
| Fun |  | Con |  | 0.3368 |  | 0.0392 |  | 0.2600 |  | 0.414 |  | 0.657 |  | 8.59 |  | < .001 |  |
| Ego |  | Con |  | 0.2216 |  | 0.0404 |  | 0.1424 |  | 0.301 |  | 0.417 |  | 5.49 |  | < .001 |  |
|  |  |  |  |  |  |  |  |  |  |  |  |  |  |  |  |  |  |
| --- | --- | --- | --- | --- | --- | --- | --- | --- | --- | --- | --- | --- | --- | --- | --- | --- | --- |
|  | | | | | | | | | | | | | | | | | |
|  | | | | | | | | | | | | | | | | | |

| Intercepts | | | | | | | | | | | | | |
| --- | --- | --- | --- | --- | --- | --- | --- | --- | --- | --- | --- | --- | --- |
|  | | | | | | 95% Confidence Intervals | | | |  | | | |
| Variable | | Intercept | | SE | | Lower | | Upper | | z | | p | |
| CAN1 |  | 1.735 |  | 0.041 |  | 1.655 |  | 1.815 |  | 42.659 |  | < .001 |  |
| CAN2 |  | 1.743 |  | 0.046 |  | 1.653 |  | 1.833 |  | 37.835 |  | < .001 |  |
| CAN3 |  | 1.961 |  | 0.054 |  | 1.855 |  | 2.068 |  | 36.228 |  | < .001 |  |
| CAN4 |  | 1.941 |  | 0.056 |  | 1.832 |  | 2.050 |  | 34.794 |  | < .001 |  |
| CAN5 |  | 3.825 |  | 0.049 |  | 3.729 |  | 3.921 |  | 77.880 |  | < .001 |  |
| CAN12 |  | 3.658 |  | 0.051 |  | 3.559 |  | 3.758 |  | 72.007 |  | < .001 |  |
| CAN8 |  | 2.614 |  | 0.054 |  | 2.509 |  | 2.720 |  | 48.746 |  | < .001 |  |
| CAN6 |  | 3.347 |  | 0.055 |  | 3.239 |  | 3.455 |  | 60.618 |  | < .001 |  |
| CAN9 |  | 3.249 |  | 0.056 |  | 3.140 |  | 3.359 |  | 58.056 |  | < .001 |  |
| CAN7 |  | 3.627 |  | 0.050 |  | 3.529 |  | 3.725 |  | 72.390 |  | < .001 |  |
| CAN10 |  | 3.018 |  | 0.057 |  | 2.907 |  | 3.129 |  | 53.137 |  | < .001 |  |
| CAN11 |  | 2.003 |  | 0.046 |  | 1.912 |  | 2.093 |  | 43.560 |  | < .001 |  |
| Fun |  | 0.000 |  | 0.000 |  | 0.000 |  | 0.000 |  |  |  |  |  |
| Ego |  | 0.000 |  | 0.000 |  | 0.000 |  | 0.000 |  |  |  |  |  |
| Con |  | 0.000 |  | 0.000 |  | 0.000 |  | 0.000 |  |  |  |  |  |
|  |  |  |  |  |  |  |  |  |  |  |  |  |  |
| --- | --- | --- | --- | --- | --- | --- | --- | --- | --- | --- | --- | --- | --- |
|  | | | | | | | | | | | | | |
|  | | | | | | | | | | | | | |

## Additional outputs

| Reliability indices | | | | | | | | | | | |
| --- | --- | --- | --- | --- | --- | --- | --- | --- | --- | --- | --- |
| Variable | | α | | ω₁ | | ω₂ | | ω₃ | | AVE | |
| Fun |  | 0.830 |  | 0.835 |  | 0.835 |  | 0.834 |  | 0.562 |  |
| Ego |  | 0.761 |  | 0.755 |  | 0.755 |  | 0.746 |  | 0.436 |  |
| Con |  | 0.835 |  | 0.839 |  | 0.839 |  | 0.832 |  | 0.571 |  |
|  |  |  |  |  |  |  |  |  |  |  |  |
| --- | --- | --- | --- | --- | --- | --- | --- | --- | --- | --- | --- |
|  | | | | | | | | | | | |
| [6] | | | | | | | | | | | |

## Covariances and correlations

| Residual covariances (lower triangle) and correlations (upper triangle) | | | | | | | | | | | | | | | | | | | | | | | | | |
| --- | --- | --- | --- | --- | --- | --- | --- | --- | --- | --- | --- | --- | --- | --- | --- | --- | --- | --- | --- | --- | --- | --- | --- | --- | --- |
|  | | CAN1 | | CAN2 | | CAN3 | | CAN4 | | CAN5 | | CAN12 | | CAN8 | | CAN6 | | CAN9 | | CAN7 | | CAN10 | | CAN11 | |
| CAN1 |  | -1.75e−7 |  | 0.10367 |  | -0.02232 |  | 0.0131 |  | -0.08783 |  | -0.05614 |  | -0.0795 |  | -0.18687 |  | -0.04658 |  | -0.01848 |  | -0.02134 |  | 0.14253 |  |
| CAN2 |  | 0.0756 |  | -1.50e−7 |  | 0.04974 |  | -0.0527 |  | -0.08073 |  | -0.03050 |  | 0.0138 |  | -0.00427 |  | -0.04379 |  | -0.07455 |  | 0.00815 |  | 0.10268 |  |
| CAN3 |  | -0.0191 |  | 0.04826 |  | -1.07e−6 |  | -0.0498 |  | 0.00326 |  | 0.05951 |  | 0.0886 |  | 0.06939 |  | -0.01730 |  | -0.01619 |  | -0.02773 |  | 0.06932 |  |
| CAN4 |  | 0.0115 |  | -0.05273 |  | -0.05855 |  | -5.73e−7 |  | 0.04135 |  | 0.09076 |  | 0.0140 |  | 0.06013 |  | -0.02883 |  | 0.00988 |  | 0.01276 |  | 0.08486 |  |
| CAN5 |  | -0.0683 |  | -0.07105 |  | 0.00338 |  | 0.0441 |  | 9.83e-7 |  | 0.07239 |  | -0.0808 |  | 0.09293 |  | 0.01677 |  | 0.07453 |  | -0.02960 |  | -0.05396 |  |
| CAN12 |  | -0.0451 |  | -0.02777 |  | 0.06367 |  | 0.1000 |  | 0.07026 |  | 3.02e-7 |  | -0.0353 |  | -0.03038 |  | -0.00759 |  | 0.06824 |  | -0.01186 |  | -0.05550 |  |
| CAN8 |  | -0.0674 |  | 0.01326 |  | 0.10010 |  | 0.0163 |  | -0.08281 |  | -0.03741 |  | 3.79e-7 |  | 0.03795 |  | 0.10456 |  | 0.07168 |  | -0.01052 |  | -0.01769 |  |
| CAN6 |  | -0.1633 |  | -0.00423 |  | 0.08070 |  | 0.0720 |  | 0.09804 |  | -0.03315 |  | 0.0437 |  | 1.14e-8 |  | -0.04616 |  | 0.01809 |  | -0.04311 |  | -0.09882 |  |
| CAN9 |  | -0.0413 |  | -0.04392 |  | -0.02039 |  | -0.0350 |  | 0.01793 |  | -0.00840 |  | 0.1221 |  | -0.05549 |  | 9.72e-9 |  | 0.08960 |  | 0.04482 |  | -0.03879 |  |
| CAN7 |  | -0.0147 |  | -0.06694 |  | -0.01708 |  | 0.0107 |  | 0.07135 |  | 0.06757 |  | 0.0749 |  | 0.01946 |  | 0.09775 |  | 3.84e-7 |  | 0.00786 |  | -0.09743 |  |
| CAN10 |  | -0.0192 |  | 0.00830 |  | -0.03317 |  | 0.0157 |  | -0.03212 |  | -0.01331 |  | -0.0125 |  | -0.05260 |  | 0.05543 |  | 0.00871 |  | -1.83e−8 |  | 0.00572 |  |
| CAN11 |  | 0.1037 |  | 0.08459 |  | 0.06712 |  | 0.0847 |  | -0.04739 |  | -0.05042 |  | -0.0170 |  | -0.09758 |  | -0.03883 |  | -0.08731 |  | 0.00581 |  | -1.59e−7 |  |
|  |  |  |  |  |  |  |  |  |  |  |  |  |  |  |  |  |  |  |  |  |  |  |  |  |  |
| --- | --- | --- | --- | --- | --- | --- | --- | --- | --- | --- | --- | --- | --- | --- | --- | --- | --- | --- | --- | --- | --- | --- | --- | --- | --- |
|  | | | | | | | | | | | | | | | | | | | | | | | | | |
|  | | | | | | | | | | | | | | | | | | | | | | | | | |

## Path Model

### Path diagrams

[7]Note: Circle layout requires rotation to be `Exogenous Top` or `Exogenous Bottom`. Rotation has been set to `Exogenous Top` Note: Circle layout requires rotation to be `Exogenous Top` or `Exogenous Bottom`. Rotation has been set to `Exogenous Top` Note: Circle layout requires rotation to be `Exogenous Top` or `Exogenous Bottom`. Rotation has been set to `Exogenous Top` Note: Circle layout requires rotation to be `Exogenous Top` or `Exogenous Bottom`. Rotation has been set to `Exogenous Top` Note: Circle layout requires rotation to be `Exogenous Top` or `Exogenous Bottom`. Rotation has been set to `Exogenous Top` Note: Circle layout requires rotation to be `Exogenous Top` or `Exogenous Bottom`. Rotation has been set to `Exogenous Top` Note: Circle layout requires rotation to be `Exogenous Top` or `Exogenous Bottom`. Rotation has been set to `Exogenous Top` Note: Circle layout requires rotation to be `Exogenous Top` or `Exogenous Bottom`. Rotation has been set to `Exogenous Top` Note: Circle layout requires rotation to be `Exogenous Top` or `Exogenous Bottom`. Rotation has been set to `Exogenous Top` Note: Circle layout requires rotation to be `Exogenous Top` or `Exogenous Bottom`. Rotation has been set to `Exogenous Top`

# References

[1]
The jamovi project (2022). *jamovi*. (Version 2.3) [Computer Software]. Retrieved from https://www.jamovi.org.

[2]
R Core Team (2021). *R: A Language and environment for statistical computing*. (Version 4.1) [Computer software]. Retrieved from https://cran.r-project.org. (R packages retrieved from MRAN snapshot 2022-01-01).

[3]
Revelle, W. (2019). *psych: Procedures for Psychological, Psychometric, and Personality Research*. [R package]. Retrieved from https://cran.r-project.org/package=psych.

[4]
Gallucci, M., Jentschke, S. (2021). *SEMLj: jamovi SEM Analysis*. [jamovi module]. For help please visit https://semlj.github.io/.

[5]
Rosseel, Y. (2019). lavaan: An R Package for Structural Equation Modeling. *Journal of Statistical Software, 48(2), 1-36. link*.

[6]
Jorgensen, T. D., Pornprasertmanit, S., Schoemann, A. M., Rosseel, Y., Miller, P., Quick, C., Garnier-Villarreal, M., Selig, J., Boulton, A., Preacher, K., Coffman, D., Rhemtulla, M., Robitzsch, A., Enders, C., Arslan, R., Clinton, B., Panko, P., Merkle, E., Chesnut, S., Byrnes, J., Rights, J. D., Longo, Y., Mansolf, M., Ben-Shachar, M. S., Rönkkö, M. (2019). *semTools: Useful Tools for Structural Equation Modeling*. [R Package]. Retrieved from https://CRAN.R-project.org/package=semTools.

[7]
Epskamp S. , Stuber S., Nak J., Veenman M,, Jorgensen T.D. (2019). *semPlot: Path Diagrams and Visual Analysis of Various SEM Packages' Output*. [R Package]. Retrieved from https://CRAN.R-project.org/package=semPlot.
