# Supplementary material for: Development and Validation of the Cheers Attitudes towards Non-drinkers Scale (CANS)
Source: J Health Psychol. 2024 Jan 29;29(10):1101–14. doi: 10.1177/13591053231220519 (PMC11344955; doi:10.1177/13591053231220519)
Supplement: sj-pdf-4-hpq-10.1177_13591053231220519 – Supplemental material for Development and Validation of the Cheers Attitudes towards Non-drinkers Scale (CANS) [file sj-pdf-4-hpq-10.1177_13591053231220519.pdf]

Results

Exploratory Factor Analysis Phase 1

```
jmv::efa(
    data = data,
    vars = vars(CAN29, CAN28, CAN27, CAN26, CAN25, CAN24, CAN23, CAN22, CAN21, CAN20, CAN19, CAN18, CAN17, CAN16, CAN15, CAN14, CAN13, CAN12, CAN11
    nFactorMethod = "fixed",
    nFactors = 3,
    minEigen = 1,
    extraction = "pa",
    screePlot = TRUE,
    eigen = TRUE,
    factorCor = TRUE,
    factorSummary = TRUE,
    modelFit = TRUE,
    kmo = TRUE,
    bartlett = TRUE)
```

Factor Loadings

	Factor			
	1	2	3	Uniqueness
CAN29	0.334		0.570	0.463
CAN28			0.660	0.574
CAN27			0.704	0.483
CAN26	0.542		0.451	0.389
CAN25	0.623			0.514
CAN24			0.735	0.445
CAN23	0.628			0.506
CAN22			0.679	0.501
CAN21	0.562		0.454	0.433
CAN20			0.664	0.475
CAN19	0.781			0.387
CAN18	0.373	0.471		0.405
CAN17	0.614			0.399
CAN16		0.587		0.385
CAN15	0.463	0.385		0.426
CAN14	0.581			0.568
CAN13	0.752			0.366
CAN12	0.641			0.441
CAN11	0.646			0.589
CAN10	0.355			0.789
CAN9		0.707		0.502
CAN8		0.780		0.378
CAN7		0.520		0.487
CAN6		0.753		0.484
CAN5	0.343	0.312		0.654
CAN4	0.345	0.506		0.440
CAN3	0.590			0.622
CAN2		0.561		0.466
CAN1		0.550		0.650

Note. 'Principal axis factoring' extraction method was used in combination with a 'oblimin' rotation

[3]

Factor Statistics

Summary

Factor	SS Loadings	% of Variance	Cumulative %
1	6.55	22.6	22.6
2	4.77	16.4	39.0
3	3.45	11.9	50.9

Inter-Factor Correlations

	1	2	3
1	—	0.591	0.199
2		—	0.317
3			—

Model Fit

Model Fit Measures

	RMSEA 90% CI				Model Test		
RMSEA	Lower	Upper	TLI	BIC	χ^2	df	p
0.0703	0.0655	0.0754	0.872	-949	1000	322	<.001

Assumption Checks

Bartlett's Test of Sphericity

χ^2	df	p
7125	406	< .001

KMO Measure of Sampling Adequacy

	MSA
Overall	0.940
CAN29	0.946
CAN28	0.830
CAN27	0.808
CAN26	0.949
CAN25	0.946
CAN24	0.800
CAN23	0.952
CAN22	0.894
CAN21	0.934
CAN20	0.885
CAN19	0.960
CAN18	0.956
CAN17	0.962
CAN16	0.954
CAN15	0.956
CAN14	0.957
CAN13	0.952
CAN12	0.960
CAN11	0.941
CAN10	0.923
CAN9	0.942
CAN8	0.958
CAN7	0.955
CAN6	0.919
CAN5	0.954
CAN4	0.944
CAN3	0.920
CAN2	0.960
CAN1	0.943

Eigenvalues

Initial Eigenvalues

Factor	Eigenvalue
1	10.67933
2	2.45138
3	1.09252
4	0.49968
5	0.23940
6	0.21252
7	0.17041
8	0.11018
9	0.09669
10	-0.00140
11	-0.03693
12	-0.07776
13	-0.09772
14	-0.10408
15	-0.11670
16	-0.15498
17	-0.17520
18	-0.19987
19	-0.21620
20	-0.22655
21	-0.23652
22	-0.25894
23	-0.27381
24	-0.32900
25	-0.35436
26	-0.36852
27	-0.43229
28	-0.54945
29	-0.66238

Scree Plot

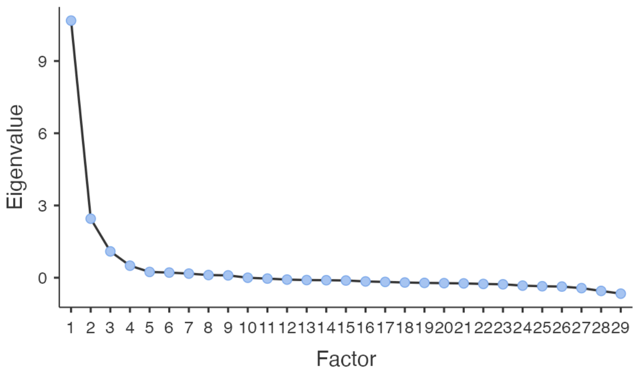

Exploratory Factor Analysis Phase 2

```
jmv::efa(
    data = data,
    vars = vars(CAN28, CAN27, CAN24, CAN23, CAN22, CAN20, CAN19, CAN17, CAN14, CAN13, CAN12, CAN9, CAN8, CAN7, CAN6, CAN3, CAN2, CAN1, CAN25, CAN11
    nFactorMethod = "fixed",
    nFactors = 3,
    minEigen = 1,
    extraction = "pa",
    screePlot = TRUE,
    eigen = TRUE,
    factorCor = TRUE,
    factorSummary = TRUE,
    modelFit = TRUE,
    kmo = TRUE,
    bartlett = TRUE,
    duplicate = 2)
```

Factor Loadings

	Factor			
	1	2	3	Uniqueness
CAN28			0.674	0.565
CAN27			0.758	0.424
CAN24			0.782	0.392
CAN23	0.580			0.558
CAN22			0.606	0.591
CAN20			0.672	0.463
CAN19	0.828			0.366
CAN17	0.698			0.382
CAN14	0.603			0.586
CAN13	0.848			0.311
CAN12	0.692			0.430
CAN9		0.737		0.458
CAN8		0.808		0.351
CAN7	0.317	0.473		0.496
CAN6		0.758		0.470
CAN3	0.594			0.635
CAN2	0.301	0.504		0.482
CAN1		0.511		0.657
CAN25	0.632			0.538
CAN11	0.660			0.583
CAN16		0.531		0.416

Note. 'Principal axis factoring' extraction method was used in combination with a 'oblimin' rotation

[3]

Factor Statistics

Summary

Factor	SS Loadings	% of Variance	Cumulative %
1	5.00	23.8	23.8
2	3.23	15.4	39.2
3	2.62	12.5	51.6

Inter-Factor Correlations

	1	2	3
1	—	0.592	0.119
2		—	0.343
3			—

Model Fit

Model Fit Measures

	RMSEA 90% CI				Model Test		
RMSEA	Lower	Upper	TLI	BIC	χ^2	df	p
0.0616	0.0543	0.0692	0.920	-515	393	150	< .001

Assumption Checks

Bartlett's Test of Sphericity

χ^2	df	p
4470	210	< .001

KMO Measure of Sampling Adequacy

	MSA
Overall	0.922
CAN28	0.829
CAN27	0.789
CAN24	0.770
CAN23	0.960
CAN22	0.872
CAN20	0.853
CAN19	0.938
CAN17	0.942
CAN14	0.949
CAN13	0.936
CAN12	0.940
CAN9	0.925
CAN8	0.931
CAN7	0.954
CAN6	0.903
CAN3	0.909
CAN2	0.952
CAN1	0.927
CAN25	0.941
CAN11	0.935
CAN16	0.955

Eigenvalues

Initial Eigenvalues

Factor	Eigenvalue
1	7.2415
2	2.0994
3	0.8446
4	0.2290
5	0.1252
6	0.1081
7	0.0576
8	0.0143
9	-0.0526
10	-0.1101
11	-0.1267
12	-0.1440
13	-0.1784
14	-0.1952
15	-0.2104
16	-0.2803
17	-0.2933
18	-0.3016
19	-0.3964
20	-0.5274
21	-0.6618

Scree Plot

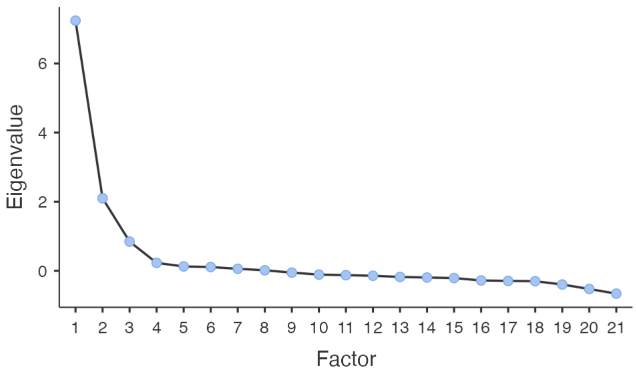

Exploratory Factor Analysis Phase 3

```
jmv::efa(
    data = data,
    vars = vars(CAN28, CAN27, CAN24, CAN23, CAN22, CAN20, CAN19, CAN17, CAN14, CAN13, CAN12, CAN9, CAN8, CAN6, CAN1, CAN25, CAN11, CAN3, CAN16),
    nFactorMethod = "fixed",
    nFactors = 3,
    minEigen = 1,
    extraction = "pa",
    sortLoadings = TRUE,
    screePlot = TRUE,
    eigen = TRUE,
    factorCor = TRUE,
    factorSummary = TRUE,
    modelFit = TRUE,
    kmo = TRUE,
    bartlett = TRUE,
    duplicate = 2)
```

Factor Loadings

	Factor			
	1	2	3	Uniqueness
CAN13	0.849			0.311
CAN19	0.827			0.364
CAN17	0.704			0.377
CAN12	0.700			0.439
CAN11	0.656			0.576
CAN25	0.641			0.546
CAN14	0.599			0.584
CAN3	0.598			0.633
CAN23	0.588			0.559
CAN24		0.779		0.394
CAN27		0.753		0.426
CAN28		0.683		0.559
CAN20		0.675		0.463
CAN22		0.596		0.592
CAN8			0.781	0.357
CAN6			0.761	0.452
CAN9			0.738	0.442
CAN16	0.315		0.527	0.406
CAN1			0.456	0.684

Note. 'Principal axis factoring' extraction method was used in combination with a 'oblimin' rotation

[3]

Factor Statistics

Summary

Factor	SS Loadings	% of Variance	Cumulative %
1	4.72	24.9	24.9
2	2.60	13.7	38.6
3	2.51	13.2	51.8

Inter-Factor Correlations

	1	2	3
1	—	0.123	0.561
2		—	0.356
3			—

Model Fit

Model Fit Measures

	RMSEA 90% CI				Model Test		
RMSEA	Lower	Upper	TLI	BIC	χ^2	df	p
0.0643	0.0561	0.0728	0.918	-385	323	117	< .001

Assumption Checks

Bartlett's Test of Sphericity

χ^2	df	p
3886	171	< .001

KMO Measure of Sampling Adequacy

	MSA
Overall	0.908
CAN28	0.822
CAN27	0.791
CAN24	0.771
CAN23	0.952
CAN22	0.875
CAN20	0.843
CAN19	0.929
CAN17	0.938
CAN14	0.942
CAN13	0.927
CAN12	0.931
CAN9	0.910
CAN8	0.910
CAN6	0.878
CAN1	0.932
CAN25	0.940
CAN11	0.935
CAN3	0.897
CAN16	0.944

Eigenvalues

Initial Eigenvalues

Factor	Eigenvalue
1	6.28051
2	2.10399
3	0.75427
4	0.20675
5	0.09922
6	0.08268
7	0.00130
8	-0.04661
9	-0.11745
10	-0.13659
11	-0.14195
12	-0.17862
13	-0.19513
14	-0.27295
15	-0.28587
16	-0.31271
17	-0.38102
18	-0.52334
19	-0.65606

Scree Plot

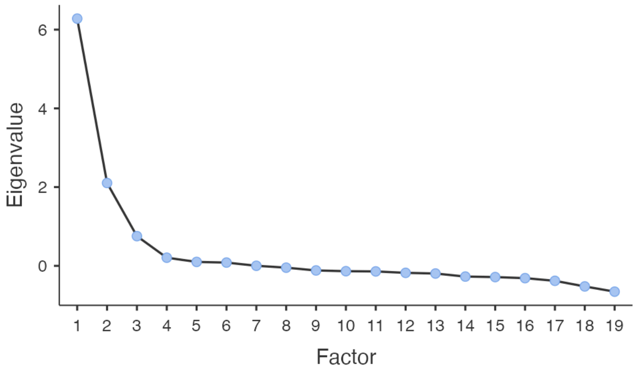

Exploratory Factor Analysis Final Solution

```
jmv::efa(
    data = data,
    vars = vars(CAN28, CAN27, CAN24, CAN22, CAN14, CAN13, CAN12, CAN9, CAN8, CAN6, CAN1, CAN25),
    minEigen = 1,
    extraction = "pa",
    sortLoadings = TRUE,
    screePlot = TRUE,
    eigen = TRUE,
    kmo = TRUE,
    duplicate = 2)
```

Factor Loadings

	Factor			
	**1**	**2**	**3**	**Uniqueness**
CAN13	0.841			0.316
CAN12	0.799			0.338
CAN25	0.612			0.571
CAN14	0.599			0.592
CAN24		0.829		0.322
CAN27		0.792		0.360
CAN28		0.590		0.648
CAN22		0.531		0.636
CAN8			0.771	0.367
CAN9			0.755	0.423
CAN6			0.747	0.443
CAN1			0.463	0.700

Note. 'Principal axis factoring' extraction method was used in combination with a 'oblimin' rotation

[3]

Assumption Checks

KMO Measure of Sampling Adequacy

	MSA
Overall	0.826
CAN28	0.836
CAN27	0.722
CAN24	0.708
CAN22	0.854
CAN14	0.857
CAN13	0.812
CAN12	0.844
CAN9	0.846
CAN8	0.846
CAN6	0.831
CAN1	0.885
CAN25	0.893

Eigenvalues

Initial Eigenvalues

Factor	Eigenvalue
1	3.5790
2	1.3236
3	0.6248
4	0.0587
5	-0.0817
6	-0.1261
7	-0.1716
8	-0.1893
9	-0.2318
10	-0.2721
11	-0.3607
12	-0.5740

Scree Plot

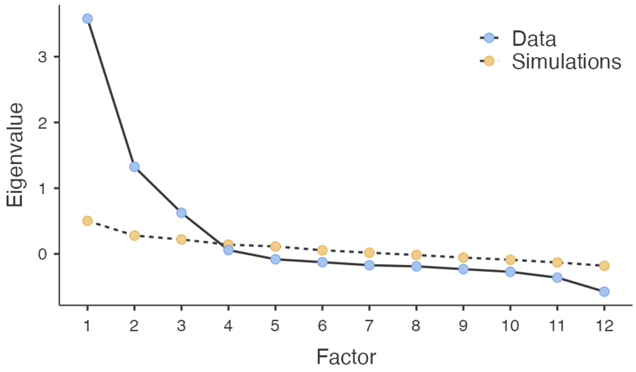

Descriptives

```
jmv::descriptives(
    data = data,
    vars = vars(Age, Gender, Education, Student, Location),
    freq = TRUE,
    nFactorMethod = "parallel",
    nFactors = 1,
    minEigen = 1,
    extraction = "pa",
    rotation = "oblimin",
    hideLoadings = 0.3,
    sortLoadings = FALSE,
    screePlot = TRUE,
    eigen = TRUE,
    factorCor = TRUE,
    factorSummary = TRUE,
    modelFit = TRUE,
    kmo = TRUE,
    bartlett = TRUE,
    factorScoresOV = list(
        synced=list()))
```

Descriptives

	Age	Gender	Education	Student	Location
N	426	426	426	426	426
Missing	0	0	0	0	0
Mean	37.3	1.52	3.70	2.56	2.70
Median	39.0	1.00	4.00	3.00	2.00
Standard deviation	9.37	0.626	1.19	0.753	1.68
Minimum	18.0	1	1	1	1
Maximum	50.0	4	6	3	8

Frequencies

Frequencies of Gender

Levels	Counts	% of Total	Cumulative %
Male (including transgender men)	225	52.8 %	52.8 %
Female (including transgender women)	189	44.4 %	97.2 %
Prefer not to say	3	0.7 %	97.9 %
Prefer to self-describe as _________ (e.g. non-binary, gende	9	2.1 %	100.0 %

Frequencies of Education

Levels	Counts	% of Total	Cumulative %
Year 11 or below	12	2.8%	2.8%
Year 12	65	15.3%	18.1%
Graduate Certificate, Diploma (including TAFE)	87	20.4%	38.5%
Bachelor's degree	163	38.3%	76.8%
Master's degree	72	16.9%	93.7%
PhD/Doctorate	27	6.3%	100.0%

Frequencies of Student

Levels	Counts	% of Total	Cumulative %
Yes, undergraduate	68	16.0%	16.0%
Yes, postgraduate	50	11.7%	27.7%
No	308	72.3%	100.0%

Frequencies of Location

Levels	Counts	% of Total	Cumulative %
NSW	89	20.9%	20.9%
VIC	182	42.7%	63.6%
QLD	53	12.4%	76.1%
SA	35	8.2%	84.3%
WA	33	7.7%	92.0%
TAS	17	4.0%	96.0%
NT	5	1.2%	97.2%
ACT	12	2.8%	100.0%

Descriptives

```
jmv::descriptives(
    data = data,
    vars = vars(CAN13, CAN14, CAN12, CAN25, CAN22, CAN24, CAN27, CAN28, CAN6, CAN8, CAN9, CAN1),
    skew = TRUE,
    kurt = TRUE,
    sw = TRUE)
```

Descriptives

	CAN13	CAN14	CAN12	CAN25	CAN22	CAN24	CAN27	CAN28	CAN6	CAN8	CAN9	CAN1
N	426	426	426	426	426	426	426	426	426	426	426	426
Missing	0	0	0	0	0	0	0	0	0	0	0	0
Mean	1.62	1.83	1.75	1.87	3.01	3.74	3.54	2.60	3.57	2.91	3.10	2.33
Median	1.00	2.00	2.00	2.00	3.00	4.00	4.00	2.00	4.00	3.00	3.00	2.00
Standard deviation	0.792	1.01	0.921	0.965	1.26	1.05	1.14	1.14	0.975	1.14	1.14	0.993
Minimum	1	1	1	1	1	1	1	1	1	1	1	1
Maximum	5	5	5	5	5	5	5	5	5	5	5	5
Skewness	1.39	1.17	1.27	1.03	-0.299	-0.802	-0.540	0.395	-0.924	-0.272	-0.453	0.424
Std. error skewness	0.118	0.118	0.118	0.118	0.118	0.118	0.118	0.118	0.118	0.118	0.118	0.118
Kurtosis	2.03	0.570	1.08	0.417	-1.20	0.210	-0.475	-0.651	0.475	-1.07	-0.859	-0.553
Std. error kurtosis	0.236	0.236	0.236	0.236	0.236	0.236	0.236	0.236	0.236	0.236	0.236	0.236
Shapiro-Wilk W	0.734	0.769	0.758	0.799	0.864	0.861	0.889	0.901	0.825	0.879	0.870	0.881
Shapiro-Wilk p	<.001	<.001	<.001	<.001	<.001	<.001	<.001	<.001	<.001	<.001	<.001	<.001

Correlation Matrix

```
jmv::corrMatrix(
    data = data,
    vars = vars(CAN_12item_TOTAL, Fun Threat Factor, Self Threat Factor, Connection Threat Factor, RAND_Total, AUDIT_Total, Total_Volume),
    pearson = FALSE,
    spearman = TRUE,
    sig = FALSE,
    n = TRUE)
```

Correlation Matrix

		CAN_12item_TOTAL	Fun Threat Factor	Self Threat Factor	Connection Threat Factor	RAND_Total	AUDIT_Total	Total_Volume
CAN_12item_TOTAL	Spearman's rho	—						
	N	—						
Fun Threat Factor	Spearman's rho	0.727	—					
	N	426	—					
Self Threat Factor	Spearman's rho	0.698	0.228	—				
	N	426	426	—				
Connection Threat Factor	Spearman's rho	0.782	0.506	0.281	—			
	N	426	426	426	—			
RAND_Total	Spearman's rho	0.634	0.661	0.193	0.624	—		
	N	426	426	426	426	—		
AUDIT_Total	Spearman's rho	0.637	0.332	0.518	0.543	0.458	—	
	N	404	404	404	404	404	—	
Total_Volume	Spearman's rho	0.564	0.348	0.398	0.512	0.468	0.880	—
	N	418	418	418	418	418	403	—

Reliability Analysis CAN 12-items

```
jmv::reliability(
    data = data,
    vars = vars(CAN13, CAN14, CAN12, CAN25, CAN22, CAN24, CAN27, CAN28, CAN1, CAN6, CAN8, CAN9),
    meanScale = TRUE,
    sdScale = TRUE,
    alphaItems = TRUE,
    meanItems = TRUE,
    sdItems = TRUE,
    itemRestCor = TRUE)
```

Scale Reliability Statistics

	Mean	SD	Cronbach's α
scale	2.66	0.614	0.824

[3]

Item Reliability Statistics

				If item dropped
	Mean	SD	Item-rest correlation	Cronbach's α
CAN13	1.62	0.792	0.486	0.812
CAN14	1.83	1.008	0.470	0.812
CAN12	1.75	0.921	0.547	0.806
CAN25	1.87	0.965	0.495	0.810
CAN22	3.01	1.260	0.474	0.812
CAN24	3.74	1.054	0.410	0.817
CAN27	3.54	1.144	0.440	0.814
CAN28	2.60	1.144	0.370	0.821
CAN1	2.33	0.993	0.424	0.815
CAN6	3.57	0.975	0.562	0.805
CAN8	2.91	1.142	0.552	0.804
CAN9	3.10	1.139	0.561	0.804

Reliability Analysis Fun

```
jmv::reliability(
    data = data,
    vars = vars(CAN13, CAN14, CAN12, CAN25),
    meanScale = TRUE,
    sdScale = TRUE,
    alphaItems = TRUE,
    meanItems = TRUE,
    sdItems = TRUE,
    itemRestCor = TRUE)
```

Scale Reliability Statistics

	Mean	SD	Cronbach's α
scale	1.77	0.738	0.810

[3]

Item Reliability Statistics

	Mean	SD	Item-rest correlation	If item dropped Cronbach's α
CAN13	1.62	0.792	0.712	0.732
CAN14	1.83	1.008	0.562	0.797
CAN12	1.75	0.921	0.699	0.727
CAN25	1.87	0.965	0.568	0.791

Reliability Analysis Self

```
jmv::reliability(
    data = data,
    vars = vars(CAN22, CAN24, CAN27, CAN28),
    meanScale = TRUE,
    sdScale = TRUE,
    alphaItems = TRUE,
    meanItems = TRUE,
    sdItems = TRUE,
    itemRestCor = TRUE)
```

Scale Reliability Statistics

	Mean	SD	Cronbach's α
scale	3.22	0.895	0.780

[3]

Item Reliability Statistics

	Mean	SD	Item-rest correlation	If item dropped Cronbach's α
CAN22	3.01	1.26	0.499	0.776
CAN24	3.74	1.05	0.663	0.691
CAN27	3.54	1.14	0.659	0.688
CAN28	2.60	1.14	0.539	0.750

Reliability Analysis Connection

```
jmv::reliability(
    data = data,
    vars = vars(CAN1, CAN6, CAN8, CAN9),
    meanScale = TRUE,
    sdScale = TRUE,
    alphaItems = TRUE,
    meanItems = TRUE,
    sdItems = TRUE,
    itemRestCor = TRUE)
```

Scale Reliability Statistics

	mean	sd	Cronbach's α
scale	2.98	0.837	0.793

[3]

Item Reliability Statistics

	mean	sd	item-rest correlation	if item dropped Cronbach's α
CAN1	2.33	0.993	0.481	0.798
CAN6	3.57	0.975	0.619	0.736
CAN8	2.91	1.142	0.671	0.706
CAN9	3.10	1.139	0.653	0.716

References

[1] The jamovi project (2022). *jamovi*. (Version 2.3) [Computer Software]. Retrieved from https://www.jamovi.org.

[2] R Core Team (2021). *R: A Language and environment for statistical computing*. (Version 4.1) [Computer software]. Retrieved from https://cran.r-project.org. (R packages retrieved from MRAN snapshot 2022-01-01).

[3] Revelle, W. (2019). *psych: Procedures for Psychological, Psychometric, and Personality Research*. [R package]. Retrieved from https://cran.r-project.org/package=psych.
