# Supplementary material for: Development and Validation of the Cheers Attitudes towards Non-drinkers Scale (CANS)
Source: J Health Psychol. 2024 Jan 29;29(10):1101–14. doi: 10.1177/13591053231220519 (PMC11344955; doi:10.1177/13591053231220519)
Supplement: sj-pdf-6-hpq-10.1177_13591053231220519 – Supplemental material for Development and Validation of the Cheers Attitudes towards Non-drinkers Scale (CANS) [file sj-pdf-6-hpq-10.1177_13591053231220519.pdf]

Results

Descriptives

Descriptives

	Age	RANDS_TOTAL	AUDIT_TOTAL	CAN_TOTAL	Total_Volume
N	389	383	385	389	389
Missing	0	6	4	0	0
Mean	39.8	25.3	8.21	32.7	783
Median	36.0	25.0	7.00	33.0	438
Standard deviation	13.5	8.14	5.78	7.35	1013
Variance	181	66.2	33.4	54.0	1.03e+6
Range	52.0	35.0	31.0	41.0	7656
Minimum	18.0	11.0	1.00	14.0	9.00
Maximum	70.0	46.0	32.0	55.0	7665
Skewness	0.580	0.340	1.19	0.127	2.66
Std. error skewness	0.124	0.125	0.124	0.124	0.124
Kurtosis	-0.629	-0.621	1.49	-0.183	9.72
Std. error kurtosis	0.247	0.249	0.248	0.247	0.247
Shapiro-Wilk W	0.939	0.974	0.908	0.994	0.715
Shapiro-Wilk p	< .001	< .001	< .001	0.151	< .001

Plots

Age

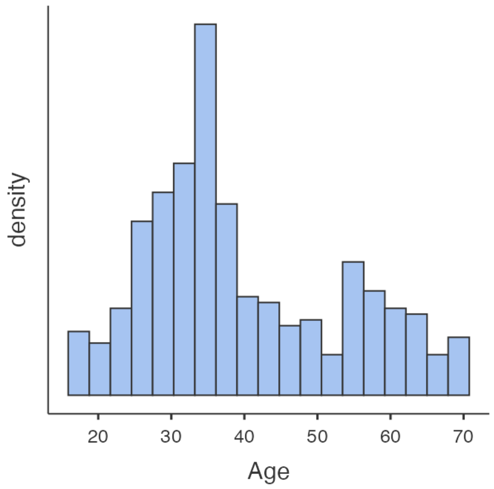

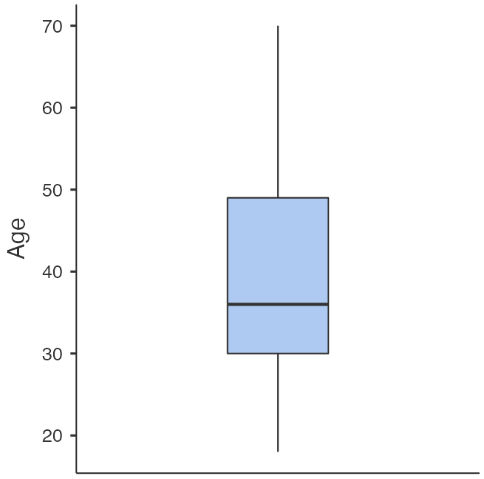

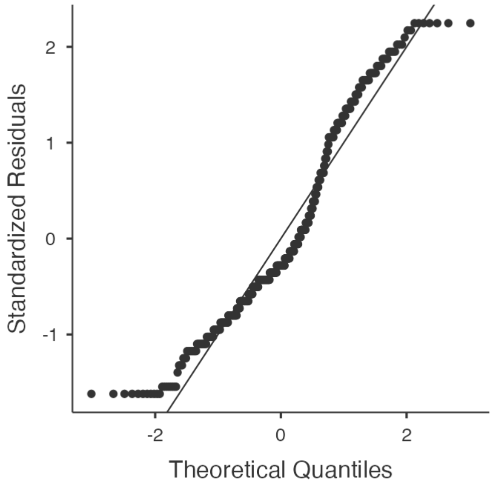
Standardized Residuals
2
1
0
-1
-2
0
2
Theoretical Quantiles

RANDS_TOTAL

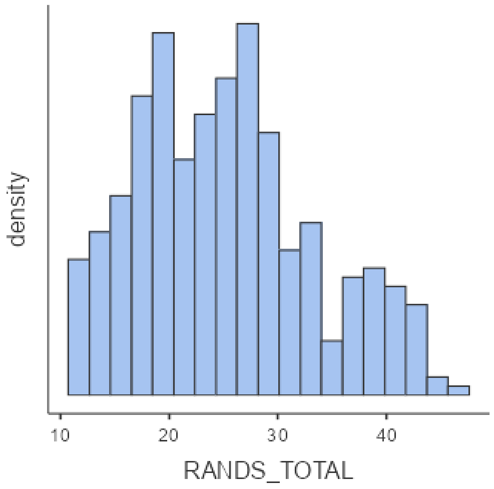
density
10
20
30
40
RANDS_TOTAL

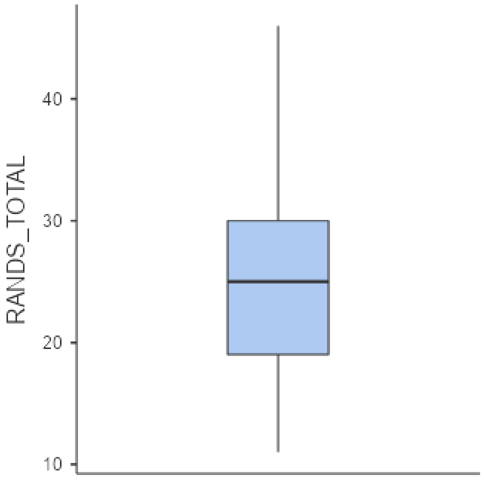
RANDS_TOTAL
40
30
20
10

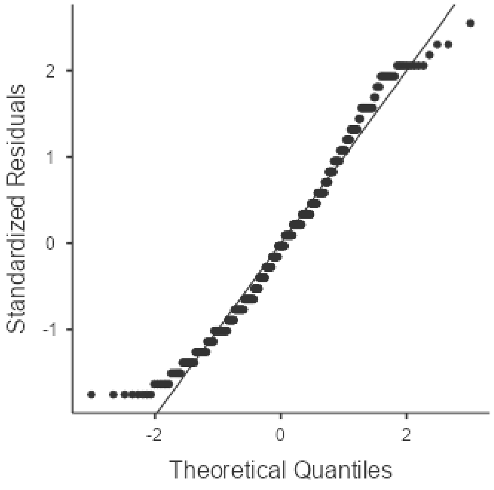
Standardized Residuals
Theoretical Quantiles
2
1
0
-1
-2
0
2

AUDIT_TOTAL

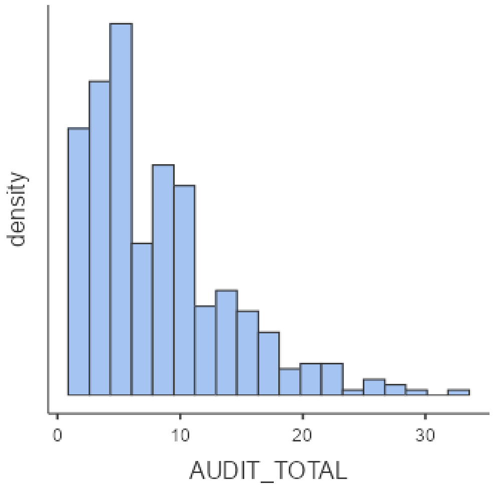
density
AUDIT_TOTAL
0
10
20
30

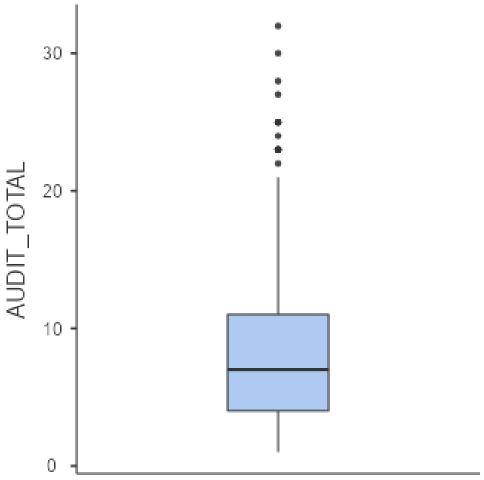
AUDIT_TOTAL
30
20
10
0

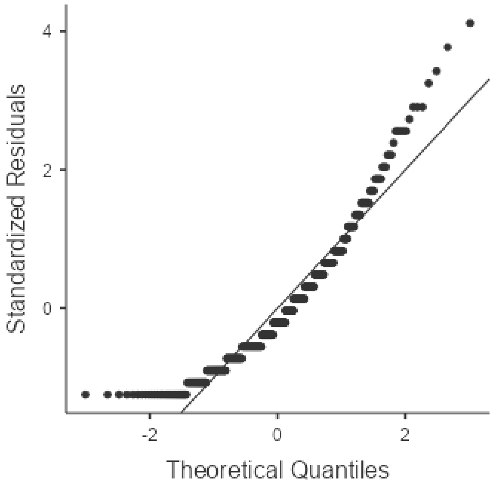
Standardized Residuals
Theoretical Quantiles

CAN_TOTAL

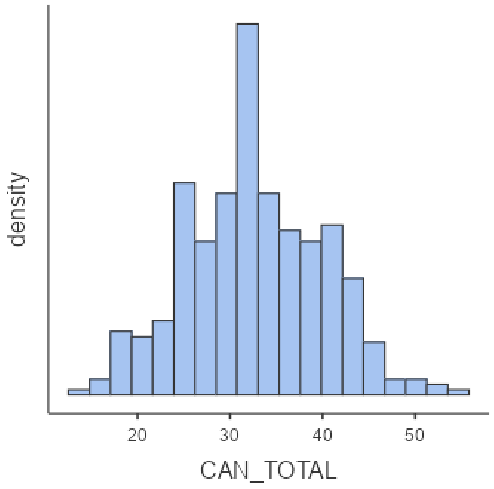
density
CAN_TOTAL

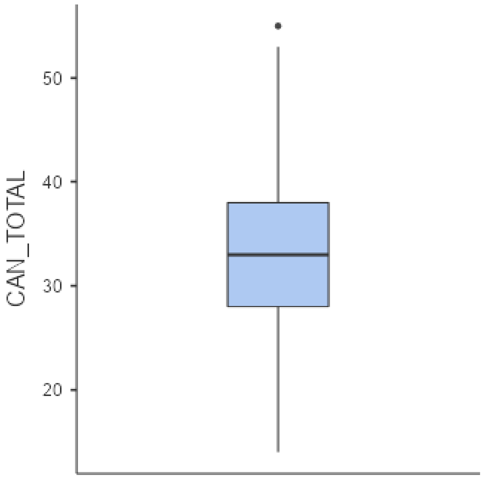
CAN_TOTAL

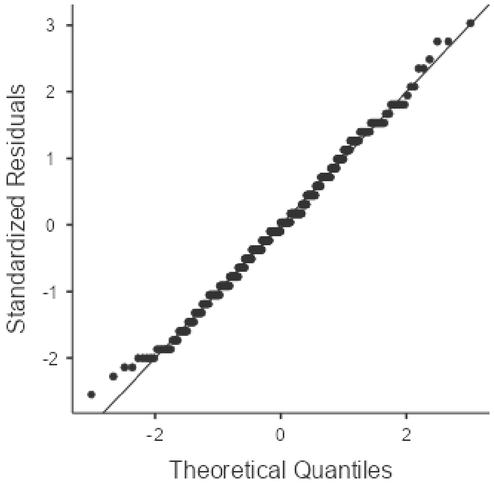

Total_Volume

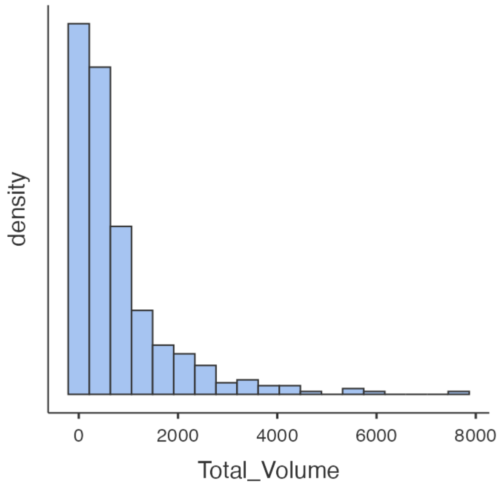

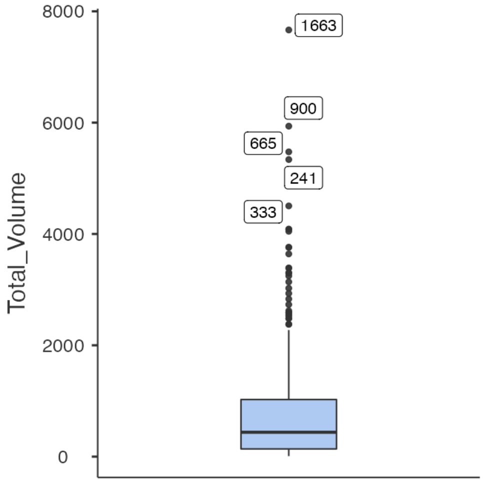

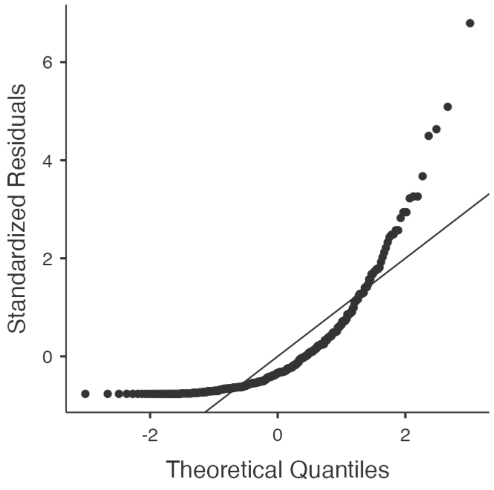

Descriptives

Frequencies

Frequencies of Location

Location	Counts	% of Total	Cumulative %
ACT	9	2.3 %	2.3 %
NSW	102	26.2 %	28.5 %
NT	3	0.8 %	29.3 %
QLD	33	8.5 %	37.8 %
SA	19	4.9 %	42.7 %
TAS	4	1.0 %	43.7 %
VIC	194	49.9 %	93.6 %
WA	25	6.4 %	100.0 %

Frequencies of Gender

Gender	Counts	% of Total	Cumulative %
Male (including transgender men)	169	43.4 %	43.4 %
Female (including transgender women)	211	54.2 %	97.7 %
Prefer not to say	2	0.5 %	98.2 %
Prefer to self-describe as __________ (e.g. non-binary, gende	7	1.8 %	100.0 %

Frequencies of Gender_other

Gender_other	Counts	% of Total	Cumulative %
queer	1	9.1 %	9.1 %
Demigirl	2	18.2 %	27.3 %
non-binary	1	9.1 %	36.4 %
Non-binary	2	18.2 %	54.5 %
Non binary	3	27.3 %	81.8 %
Biological female	1	9.1 %	90.9 %
A person of female sex with non-binary gender	1	9.1 %	100.0 %

Frequencies of Nationality

Nationality	Counts	% of Total	Cumulative %
Australian	356	91.5 %	91.5 %
Other (please click to specify)	33	8.5 %	100.0 %

Correlation Matrix

Correlation Matrix

		CAN_TOTAL	Fun_CAN	Ego_CAN	Connection_CAN	RANDS_TOTAL	AUDIT_TOTAL	Total_Volume	Age	Gender MF
CAN_TOTAL	Spearman's rho	—								
	p-value	—								
	N	—								
Fun_CAN	Spearman's rho	0.748 ***	—							
	p-value	< .001	—							
	N	389	—							
Ego_CAN	Spearman's rho	0.656 ***	0.185 ***	—						
	p-value	< .001	< .001	—						
	N	389	389	—						
Connection_CAN	Spearman's rho	0.828 ***	0.552 ***	0.322 ***	—					
	p-value	< .001	< .001	< .001	—					
	N	389	389	389	—					
RANDS_TOTAL	Spearman's rho	0.677 ***	0.734 ***	0.195 ***	0.607 ***	—				
	p-value	< .001	< .001	< .001	< .001	—				
	N	383	383	383	383	—				
AUDIT_TOTAL	Spearman's rho	0.542 ***	0.419 ***	0.380 ***	0.429 ***	0.506 ***	—			
	p-value	< .001	< .001	< .001	< .001	< .001	—			
	N	385	385	385	385	381	—			
Total_Volume	Spearman's rho	0.386 ***	0.318 ***	0.254 ***	0.288 ***	0.427 ***	0.807 ***	—		
	p-value	< .001	< .001	< .001	< .001	< .001	< .001	—		
	N	389	389	389	389	383	385	—		
Age	Spearman's rho	-0.251 ***	-0.163 **	-0.079	-0.330 ***	-0.194 ***	-0.192 ***	-0.111 *	—	
	p-value	< .001	0.001	0.118	< .001	< .001	< .001	0.028	—	
	N	389	389	389	389	383	385	389	—	
Gender MF	Spearman's rho	-0.129 *	-0.123 *	-0.087	-0.096	-0.144 **	-0.251 ***	-0.292 ***	-0.033	—
	p-value	0.012	0.016	0.091	0.062	0.005	< .001	< .001	0.522	—
	N	380	380	380	380	375	378	380	380	—

Note. * p < .05, ** p < .01, *** p < .001

Reliability Analysis

Scale Reliability Statistics

	Cronbach's α
scale	0.842

[3]

Item Reliability Statistics

		if item dropped
	item-rest correlation	Cronbach's α
CAN2	0.537	0.828
CAN3	0.553	0.826
CAN4	0.566	0.825
CAN5	0.368	0.839
CAN6	0.348	0.842
CAN7	0.589	0.824
CAN8	0.424	0.836
CAN9	0.636	0.819
CAN10	0.633	0.819
CAN12	0.406	0.837
CAN1	0.480	0.832
CAN11	0.549	0.827

Reliability Analysis

Scale Reliability Statistics

	Cronbach's α
scale	0.830

[3]

Item Reliability Statistics

		if item dropped
	item-rest correlation	Cronbach's α
CAN1	0.693	0.780
CAN2	0.716	0.763
CAN3	0.642	0.796
CAN4	0.625	0.807

Reliability Analysis

Scale Reliability Statistics

	Cronbach's α
scale	0.761

[3]

Item Reliability Statistics

		if item dropped
	item-rest correlation	Cronbach's α
CAN5	0.567	0.701
CAN6	0.573	0.697
CAN8	0.537	0.716
CAN12	0.561	0.703

Reliability Analysis

Scale Reliability Statistics

	Cronbach's α
scale	0.835

[3]

Item Reliability Statistics

		if item dropped
	item-rest correlation	Cronbach's α
CAN11	0.551	0.838
CAN9	0.748	0.752
CAN10	0.742	0.756
CAN7	0.634	0.805

Correlation Matrix

Correlation Matrix

		Fun_CAN	Ego_CAN	Connection_CAN
Fun_CAN	Pearson's r	—		
	p-value	—		
Ego_CAN	Pearson's r	0.183 ***	—	
	p-value	< .001	—	
Connection_CAN	Pearson's r	0.555 ***	0.327 ***	—
	p-value	< .001	< .001	—

Note. * p < .05, ** p < .01, *** p < .001

Descriptives

Descriptives

	CAN1	CAN2	CAN3	CAN4	CAN5	CAN6	CAN7	CAN8	CAN9	CAN10	CAN11	CAN12
N	389	389	389	389	389	389	389	389	389	389	389	389
Missing	0	0	0	0	0	0	0	0	0	0	0	0
Mean	1.74	1.74	1.96	1.94	3.83	3.35	3.63	2.61	3.25	3.02	2.00	3.66
Median	2	2	2	2	4	4	4	2	3	3	2	4
Standard deviation	0.802	0.909	1.07	1.10	0.969	1.09	0.988	1.06	1.10	1.12	0.907	1.00
Minimum	1	1	1	1	1	1	1	1	1	1	1	1
Maximum	4	5	5	5	5	5	5	5	5	5	5	5
Skewness	0.966	1.24	0.920	0.946	-0.875	-0.422	-0.856	0.426	-0.425	-0.0687	0.746	-0.432
Std. error skewness	0.124	0.124	0.124	0.124	0.124	0.124	0.124	0.124	0.124	0.124	0.124	0.124
Kurtosis	0.477	0.883	-0.218	-0.343	0.609	-0.714	0.285	-0.489	-0.656	-1.03	-0.0309	-0.502
Std. error kurtosis	0.247	0.247	0.247	0.247	0.247	0.247	0.247	0.247	0.247	0.247	0.247	0.247

Plots

CAN1

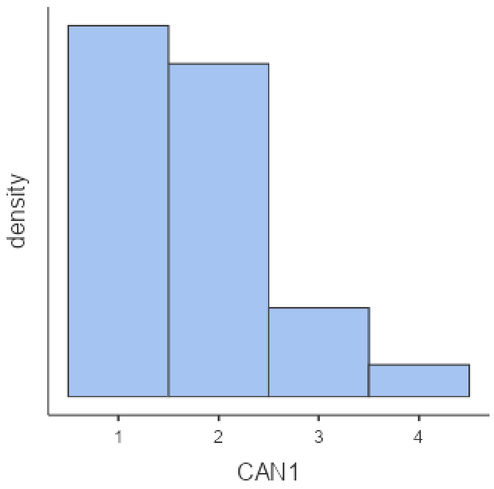

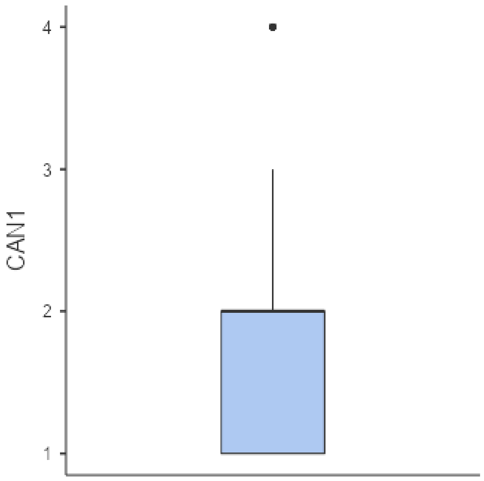

CAN2

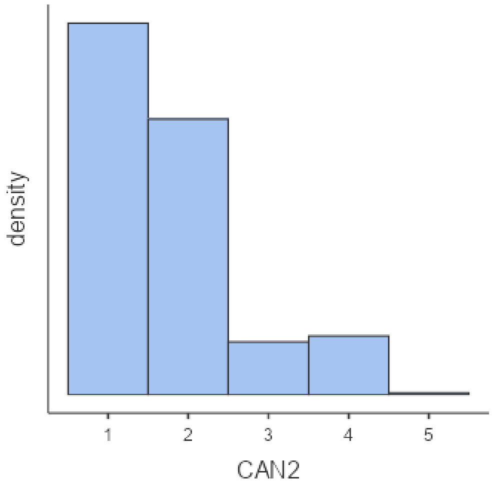

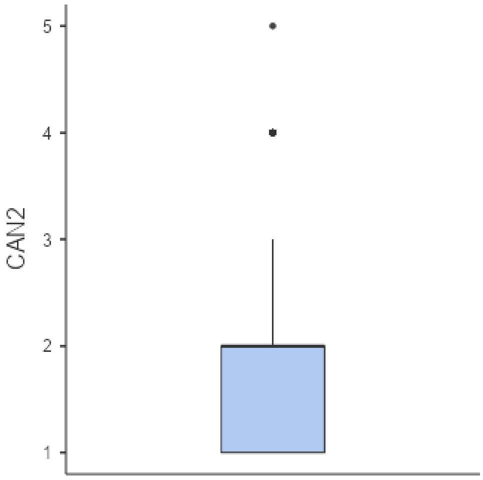

CAN3

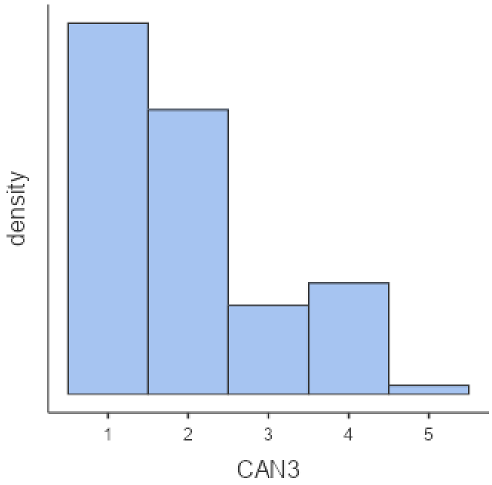

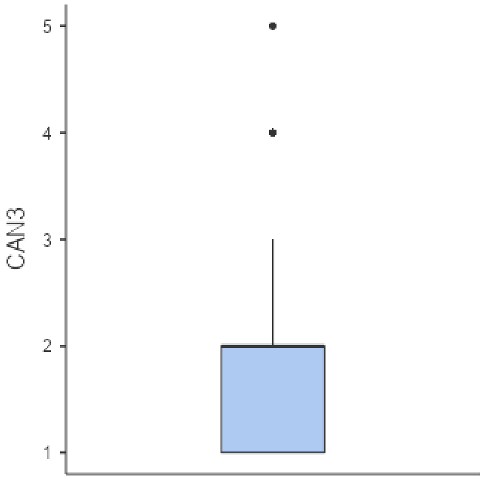

CAN4

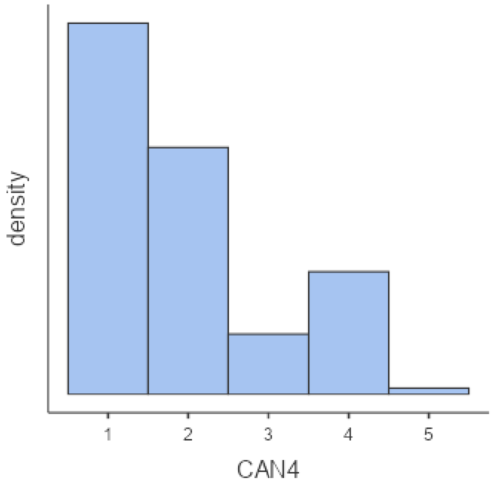

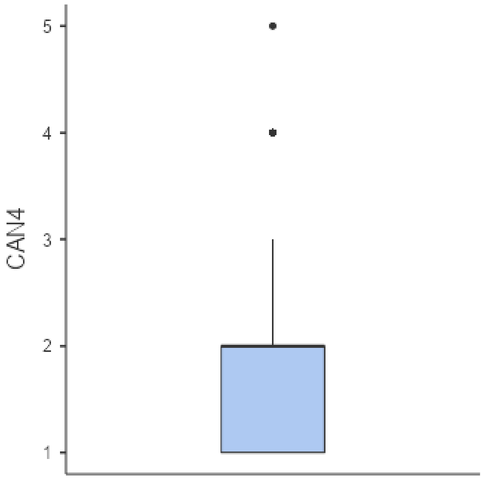

CAN5

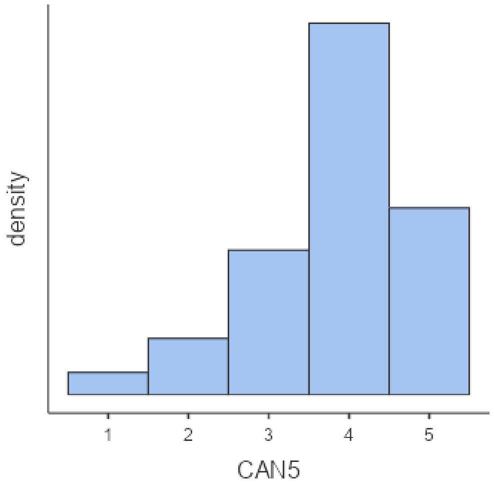

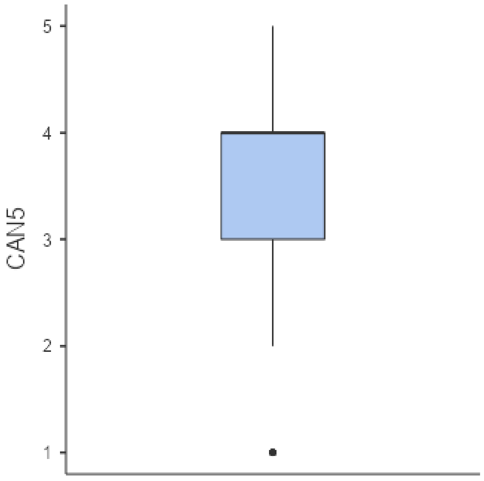

CAN6

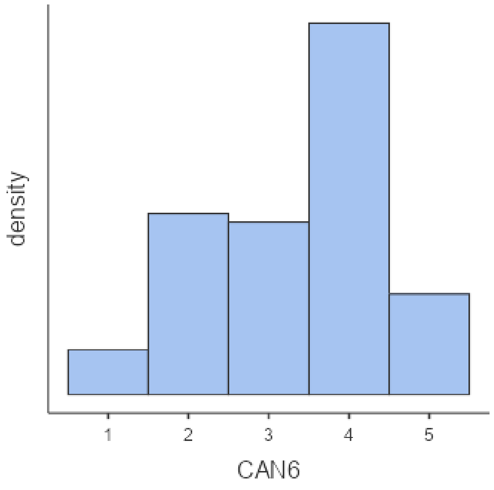

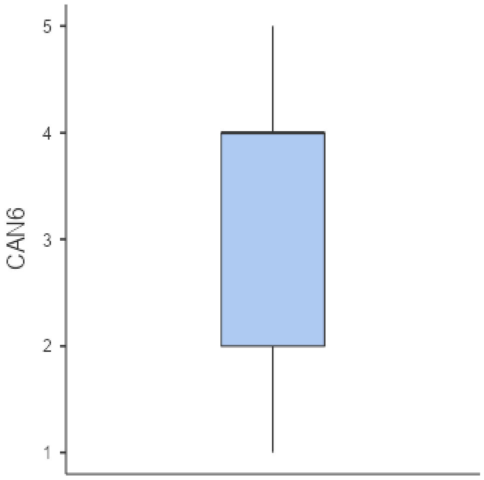

CAN7

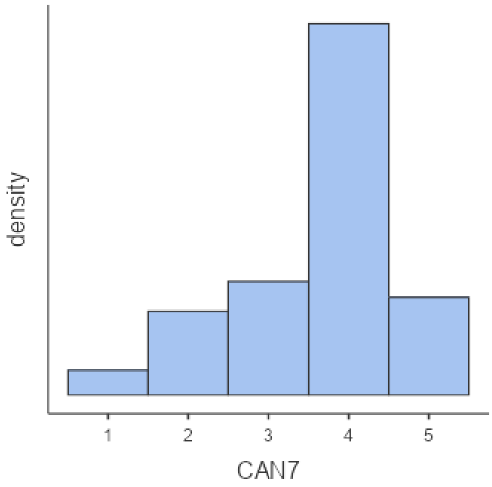

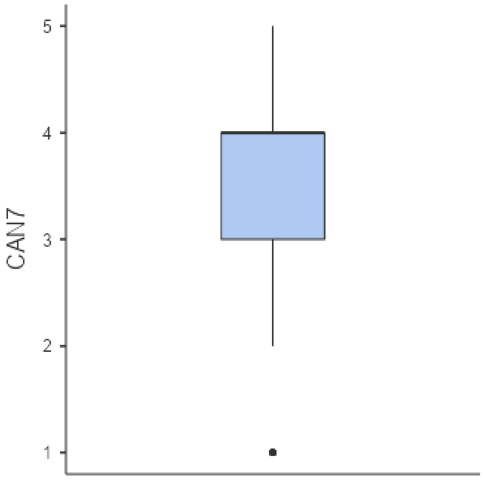

CAN8

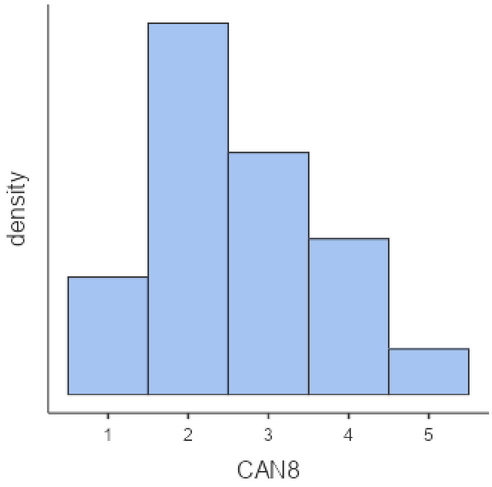

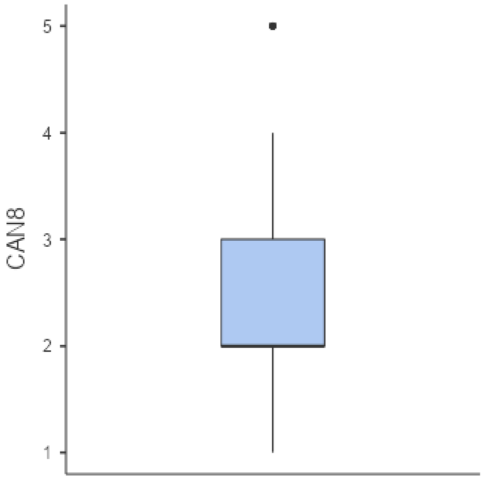

CAN9

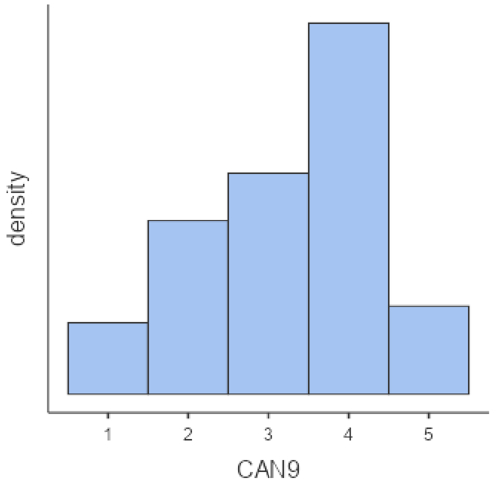

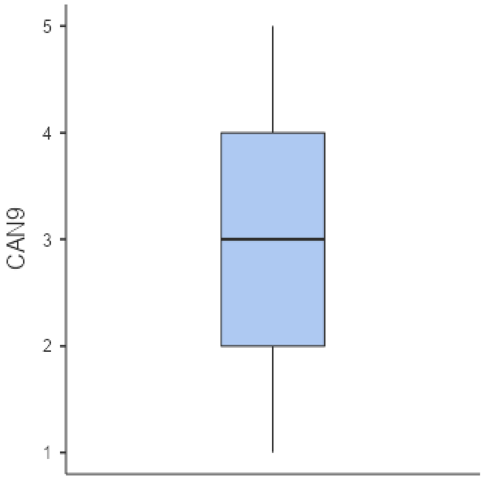

CAN10

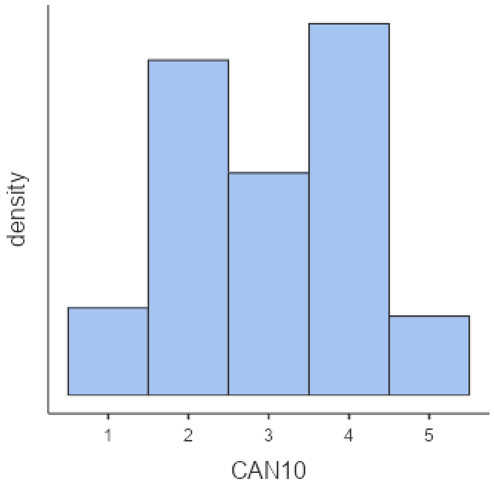

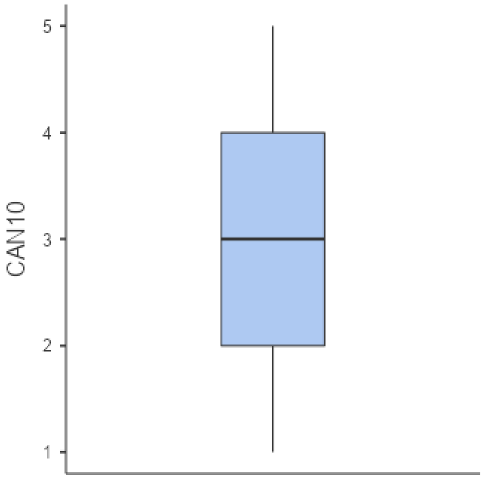

CAN11

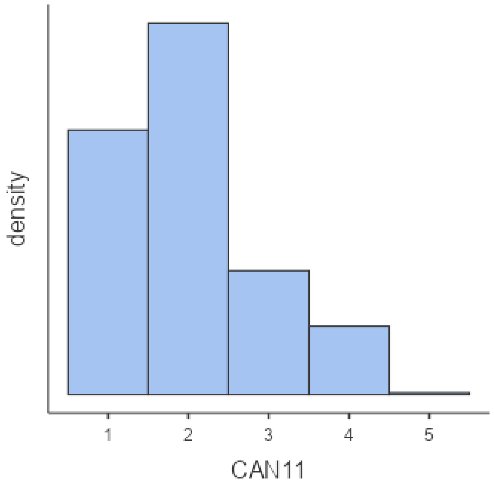

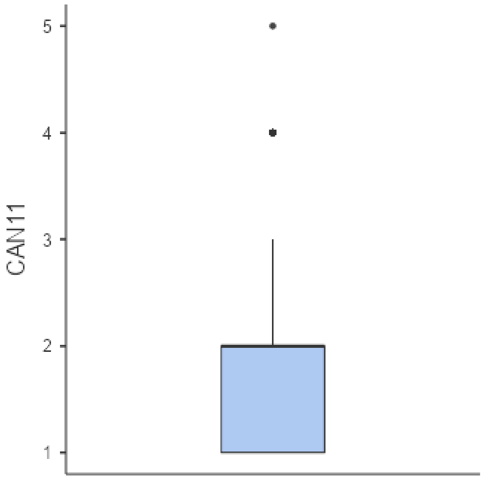

CAN12

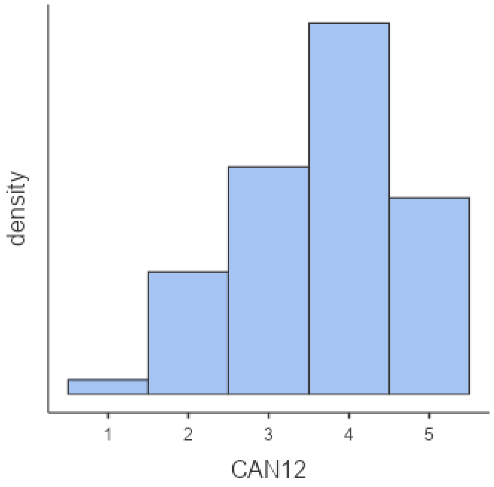

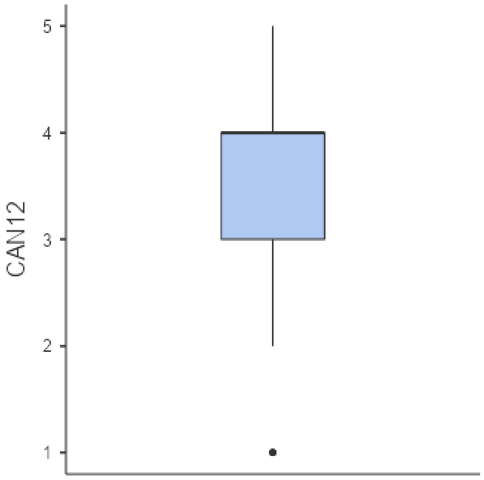

Confirmatory Factor Analysis

Models Info

Estimation Method	DWLS
Optimization Method	NLMINB
Number of observations	389
Free parameters	39
Standard errors	Robust
Scaled test	Satorra-Bentler mean adjusted
Converged	TRUE
Iterations	39
Model	Fun=~CAN1+CAN2+CAN3+CAN4
	Ego=~CAN5+CAN12+CAN8+CAN6
	Con=~CAN9+CAN7+CAN10+CAN11

[4] [5]

Overall Tests

Model tests

Label	X²	df	p
User Model	85.4	51	0.002
Baseline Model	2718.9	66	<.001
Scaled User	201.6	51	<.001
Scaled Baseline	2718.9	66	<.001

Fit indices

			95% Confidence Intervals		
Type	SRMR	RMSEA	Lower	Upper	RMSEA p
Classical	0.054	0.042	0.025	0.057	0.805
Robust	0.054	0.057	0.049	0.065	
Scaled	0.054	0.087	0.068	0.107	<.001

User model versus baseline model

	Model
Comparative Fit Index (CFI)	0.987
Tucker-Lewis Index (TLI)	0.983
Bentler-Bonett Non-normed Fit Index (NNFI)	0.983
Bentler-Bonett Normed Fit Index (NFI)	0.969
Parsimony Normed Fit Index (PNFI)	0.748
Bollen's Relative Fit Index (RFI)	0.959
Bollen's Incremental Fit Index (IFI)	0.987
Relative Noncentrality Index (RNI)	0.987

Additional fit indices

	Model
Hoelter Critical N (CN), a=0.05	313.006
Hoelter Critical N (CN), a=0.01	352.611
Goodness of Fit Index (GFI)	0.998
Parsimony Goodness of Fit Index (GFI)	0.566
McDonald Fit Index (MFI)	0.957

Estimates

Measurement model

				95% Confidence Intervals				
Latent	Observed	Estimate	SE	Lower	Upper	β	z	p
Fun	CAN1	1.000	0.0000	1.000	1.000	0.733		
	CAN2	1.178	0.0968	0.988	1.368	0.763	12.17	<.001
	CAN3	1.336	0.1324	1.077	1.596	0.736	10.10	<.001
	CAN4	1.426	0.1098	1.210	1.641	0.762	12.98	<.001
Ego	CAN5	1.000	0.0000	1.000	1.000	0.629		
	CAN12	1.121	0.1358	0.855	1.387	0.682	8.26	<.001
	CAN8	1.221	0.1687	0.891	1.552	0.703	7.24	<.001
	CAN6	1.115	0.1449	0.831	1.399	0.624	7.69	<.001
Con	CAN9	1.000	0.0000	1.000	1.000	0.790		
	CAN7	0.790	0.0547	0.683	0.898	0.697	14.44	<.001
	CAN10	1.043	0.0550	0.935	1.151	0.812	18.97	<.001
	CAN11	0.704	0.0598	0.587	0.821	0.677	11.77	<.001

Variances and Covariances

				95% Confidence Intervals				
Variable 1	Variable 2	Estimate	SE	Lower	Upper	β	z	p
CAN1	CAN1	0.2976	0.0366	0.2258	0.369	0.462	8.13	<.001
CAN2	CAN2	0.3451	0.0487	0.2496	0.441	0.418	7.08	<.001
CAN3	CAN3	0.5222	0.0640	0.3968	0.648	0.458	8.16	<.001
CAN4	CAN4	0.5070	0.0630	0.3836	0.630	0.419	8.05	<.001
CAN5	CAN5	0.5672	0.0673	0.4354	0.699	0.604	8.43	<.001
CAN12	CAN12	0.5373	0.0673	0.4054	0.669	0.535	7.98	<.001
CAN8	CAN8	0.5653	0.0675	0.4330	0.698	0.505	8.38	<.001
CAN6	CAN6	0.7247	0.0714	0.5848	0.865	0.611	10.16	<.001
CAN9	CAN9	0.4580	0.0479	0.3642	0.552	0.376	9.56	<.001
CAN7	CAN7	0.5016	0.0448	0.4139	0.589	0.514	11.21	<.001
CAN10	CAN10	0.4277	0.0510	0.3277	0.528	0.341	8.38	<.001
CAN11	CAN11	0.4450	0.0423	0.3621	0.528	0.541	10.52	<.001
Fun	Fun	0.3461	0.0501	0.2480	0.444	1.000	6.91	<.001
Ego	Ego	0.3712	0.0736	0.2270	0.515	1.000	5.04	<.001
Con	Con	0.7606	0.0806	0.6027	0.918	1.000	9.44	<.001
Fun	Ego	0.0811	0.0255	0.0311	0.131	0.226	3.18	0.001
Fun	Con	0.3368	0.0392	0.2600	0.414	0.657	8.59	<.001
Ego	Con	0.2216	0.0404	0.1424	0.301	0.417	5.49	<.001

Intercepts

			95% Confidence Intervals			
Variable	Intercept	SE	Lower	Upper	z	p
CAN1	1.735	0.041	1.655	1.815	42.659	< .001
CAN2	1.743	0.046	1.653	1.833	37.835	< .001
CAN3	1.961	0.054	1.855	2.068	36.228	< .001
CAN4	1.941	0.056	1.832	2.050	34.794	< .001
CAN5	3.825	0.049	3.729	3.921	77.880	< .001
CAN12	3.658	0.051	3.559	3.758	72.007	< .001
CAN8	2.614	0.054	2.509	2.720	48.746	< .001
CAN6	3.347	0.055	3.239	3.455	60.618	< .001
CAN9	3.249	0.056	3.140	3.359	58.056	< .001
CAN7	3.627	0.050	3.529	3.725	72.390	< .001
CAN10	3.018	0.057	2.907	3.129	53.137	< .001
CAN11	2.003	0.046	1.912	2.093	43.560	< .001
Fun	0.000	0.000	0.000	0.000		
Ego	0.000	0.000	0.000	0.000		
Con	0.000	0.000	0.000	0.000		

Additional outputs

Reliability indices

Variable	α	ω_1	ω_2	ω_3	AVE
Fun	0.830	0.835	0.835	0.834	0.562
Ego	0.761	0.755	0.755	0.746	0.436
Con	0.835	0.839	0.839	0.832	0.571

[6]

Covariances and correlations

Residual covariances (lower triangle) and correlations (upper triangle)

	CAN1	CAN2	CAN3	CAN4	CAN5	CAN12	CAN8	CAN6	CAN9	CAN7	CAN10	CAN11
CAN1	-1.75e–7	0.10367	-0.02232	0.0131	-0.08783	-0.05614	-0.0795	-0.18687	-0.04658	-0.01848	-0.02134	0.14253
CAN2	0.0756	-1.50e–7	0.04974	-0.0527	-0.08073	-0.03050	0.0138	-0.00427	-0.04379	-0.07455	0.00815	0.10268
CAN3	-0.0191	0.04826	-1.07e–6	-0.0498	0.00326	0.05951	0.0886	0.06939	-0.01730	-0.01619	-0.02773	0.06932
CAN4	0.0115	-0.05273	-0.05855	-5.73e–7	0.04135	0.09076	0.0140	0.06013	-0.02883	0.00988	0.01276	0.08486
CAN5	-0.0683	-0.07105	0.00338	0.0441	9.83e-7	0.07239	-0.0808	0.09293	0.01677	0.07453	-0.02960	-0.05396
CAN12	-0.0451	-0.02777	0.06367	0.1000	0.07026	3.02e-7	-0.0353	-0.03038	-0.00759	0.06824	-0.01186	-0.05550
CAN8	-0.0674	0.01326	0.10010	0.0163	-0.08281	-0.03741	3.79e-7	0.03795	0.10456	0.07168	-0.01052	-0.01769
CAN6	-0.1633	-0.00423	0.08070	0.0720	0.09804	-0.03315	0.0437	1.14e-8	-0.04616	0.01809	-0.04311	-0.09882
CAN9	-0.0413	-0.04392	-0.02039	-0.0350	0.01793	-0.00840	0.1221	-0.05549	9.72e-9	0.08960	0.04482	-0.03879
CAN7	-0.0147	-0.06694	-0.01708	0.0107	0.07135	0.06757	0.0749	0.01946	0.09775	3.84e-7	0.00786	-0.09743
CAN10	-0.0192	0.00830	-0.03317	0.0157	-0.03212	-0.01331	-0.0125	-0.05260	0.05543	0.00871	-1.83e–8	0.00572
CAN11	0.1037	0.08459	0.06712	0.0847	-0.04739	-0.05042	-0.0170	-0.09758	-0.03883	-0.08731	0.00581	-1.59e–7

Path Model

Path diagrams

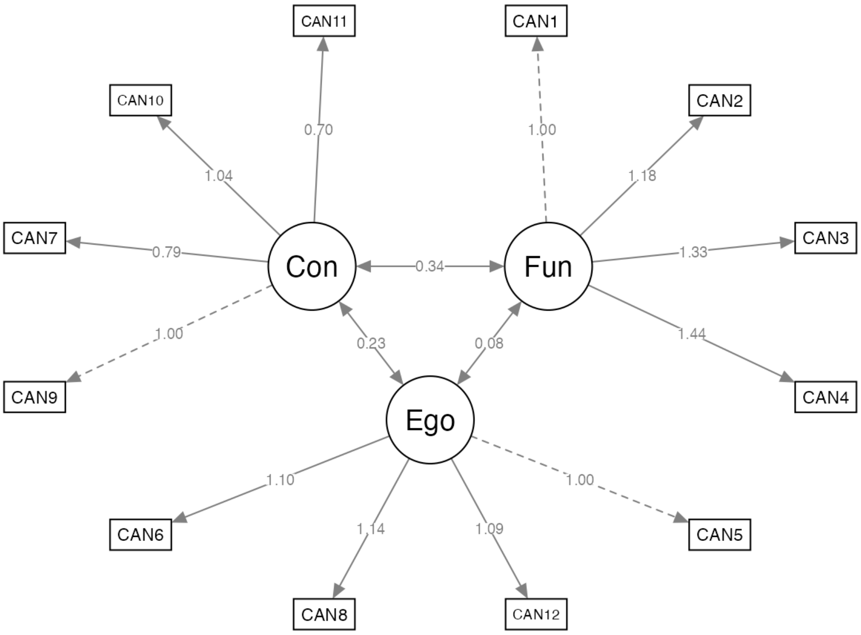

[7]

Note: Circle layout requires rotation to be `Exogenous Top` or `Exogenous Bottom`. Rotation has been set to `Exogenous Top` Note: Circle layout requires rotation to be `Exogenous Top` or `Exogenous Bottom`. Rotation has been set to `Exogenous Top` Note: Circle layout requires rotation to be `Exogenous Top` or `Exogenous Bottom`. Rotation has been set to `Exogenous Top` Note: Circle layout requires rotation to be `Exogenous Top` or `Exogenous Bottom`. Rotation has been set to `Exogenous Top` Note: Circle layout requires rotation to be `Exogenous Top` or `Exogenous Bottom`. Rotation has been set to `Exogenous Top` Note: Circle layout requires rotation to be `Exogenous Top` or `Exogenous Bottom`. Rotation has been set to `Exogenous Top` Note: Circle layout requires rotation to be `Exogenous Top` or `Exogenous Bottom`. Rotation has been set to `Exogenous Top` Note: Circle layout requires rotation to be `Exogenous Top` or `Exogenous Bottom`. Rotation has been set to `Exogenous Top` Note: Circle layout requires rotation to be `Exogenous Top` or `Exogenous Bottom`. Rotation has been set to `Exogenous Top`

References

[1] The jamovi project (2022). *jamovi*. (Version 2.3) [Computer Software]. Retrieved from https://www.jamovi.org.

[2] R Core Team (2021). *R: A Language and environment for statistical computing.* (Version 4.1) [Computer software]. Retrieved from https://cran.r-project.org. (R packages retrieved from MRAN snapshot 2022-01-01).

[3] Revelle, W. (2019). *psych: Procedures for Psychological, Psychometric, and Personality Research*. [R package]. Retrieved from https://cran.r-project.org/package=psych.

[4] Gallucci, M., Jentschke, S. (2021). *SEMLj: jamovi SEM Analysis*. [jamovi module]. For help please visit https://semlj.github.io/.

[5] Rosseel, Y. (2019). lavaan: An R Package for Structural Equation Modeling. *Journal of Statistical Software, 48(2), 1-36*. *link*.

[6] Jorgensen, T. D., Pornprasertmanit, S., Schoemann, A. M., Rosseel, Y., Miller, P., Quick, C., Garnier-Villarreal, M., Selig, J., Boulton, A., Preacher, K., Coffman, D., Rhemtulla, M., Robitzsch, A., Enders, C., Arslan, R., Clinton, B., Panko, P., Merkle, E., Chesnut, S., Byrnes, J., Rights, J. D., Longo, Y., Mansolf, M., Ben-Shachar, M. S., Rönkkö, M. (2019). *semTools: Useful Tools for Structural Equation Modeling.* [R Package]. Retrieved from https://CRAN.R-project.org/package=semTools.

[7] Epskamp S. , Stuber S., Nak J., Veenman M,, Jorgensen T.D. (2019). *semPlot: Path Diagrams and Visual Analysis of Various SEM Packages' Output.* [R Package]. Retrieved from https://CRAN.R-project.org/package=semPlot.
